# Neonatal inflammation disrupts a temporally restricted postnatal *Numb*-enriched microglial state in mice

**DOI:** 10.1038/s41467-026-76341-6

**Published:** 2026-08-04

**Authors:** Jinjin Zhu, Yiran Xu, Liubo Sun, Ziwei Huang, Wenkai Yu, Shan Zhang, Xiaoli Zhang, Tiantian He, Yiwen Chen, Yanan Wu, Bingbing Li, Huifang Dong, Xiaoyang Wang, Changlian Zhu

**Affiliations:** 1 Henan Key Laboratory of Child Brain Injury, The Third Affiliated Hospital of Zhengzhou University, Zhengzhou, China; 2 Institute of Neuroscience, Zhengzhou University, Zhengzhou, China; 3 Centre of Perinatal Medicine and Health, Institute of Clinical Science, University of Gothenburg, Gothenburg, Sweden; 4 Department of Women’s and Children’s Health, Karolinska Institutet, Stockholm, Sweden; 5 Center for Brain Repair and Rehabilitation, Institute of Neuroscience and Physiology, University of Gothenburg, Gothenburg, Sweden

**Keywords:** Development of the nervous system, Microglia, Glial development

## Abstract

Early-life microglia are diverse and support brain development beyond immune surveillance, but the transient states associated with postnatal maturation remain poorly understood. Here we show, using a time-resolved single-cell atlas of neonatal mouse brain immune cells, that a postnatal *Numb*-enriched microglial state emerges during early postnatal development, expands during the second postnatal week and subsequently declines. This state is characterized by neurodevelopment-related gene expression programs and distinct metabolic features. Trajectory inference, cross-atlas mapping and RNAscope validation support its temporal pattern. During the period when this state expands, microglial depletion preserves gross myelination but alters synaptic protein composition and disrupts dendritic and cortical layer maturation, particularly in the primary somatosensory cortex. Neonatal lipopolysaccharide challenge impairs the establishment of the *Numb*-enriched state and induces an early glycolytic response followed by recovery-phase inflammatory states. These findings identify a developmentally timed microglial state associated with cortical maturation and vulnerable to neonatal inflammation in mice.

## Abstract (web summary)

Microglia help shape the developing brain, but how their early-life states change over time remains unclear. Here, the authors show that a transient Numb-enriched state marks postnatal cortical maturation in mice and is disrupted by neonatal inflammation.

## Introduction

Microglia are integral components of the developing brain, where they support neuronal survival, synaptic refinement, myelination, and circuit assembly during early life^1,2^. These developmental functions are particularly vulnerable to perinatal immune perturbation. In preterm infants, infection and systemic inflammation are frequent clinical complications and are associated with an increased risk of long-term neurodevelopmental impairment^3^. Although microglia have been increasingly implicated in preterm brain injury, it remains unclear how inflammatory exposure disrupts their normal developmental roles. This knowledge gap reflects a broader limitation in the field: early-life microglial maturation remains incompletely resolved, and the developmental windows during which microglial states become susceptible to inflammatory perturbation remain poorly defined^4–6^.

Single-cell RNA sequencing (scRNA-seq) studies have revealed substantial microglial heterogeneity in the developing human brain^7–14^ and the early postnatal mouse brain, identifying proliferative, white matter-associated, immune-responsive, and neuron-associated microglial states^15–20^. Yet, how these states are organized across early postnatal maturation remains incompletely defined. This gap is particularly important during periods of rapid synaptogenesis, dendritic growth, and circuit assembly, when microglia are expected to coordinate with multiple developmental processes. In mice, these events are especially active during the second postnatal week, a developmental interval that has relevance to the term-equivalent human brain^21–23^. Although microglia are thought to support this phase of brain maturation^24^, the specific transcriptional programs that enable their developmental functions—and that may render them vulnerable to inflammatory disruption—remain poorly understood^16^.

Here, we show, using a time-resolved single-cell atlas of neonatal mouse brain immune cells under physiological conditions and following neonatal inflammation, that a temporally restricted postnatal *Numb*-enriched microglial state expands during the second postnatal week and exhibits neurodevelopment-related and metabolic transcriptional programs. Microglial depletion during the period in which this state expands alters synaptic protein composition, dendritic architecture, and cortical laminar maturation. Neonatal inflammation reduces the representation of the *Numb*-enriched state and is associated with an early acute post-inflammatory microglial response followed by later recovery-phase inflammatory states. Together, these findings identify a developmentally timed microglial state associated with cortical maturation and vulnerable to early-life inflammation.

## Results

### A time-resolved single-cell atlas maps the physiological remodeling of microglial states during early postnatal development

**Fig. 1:**
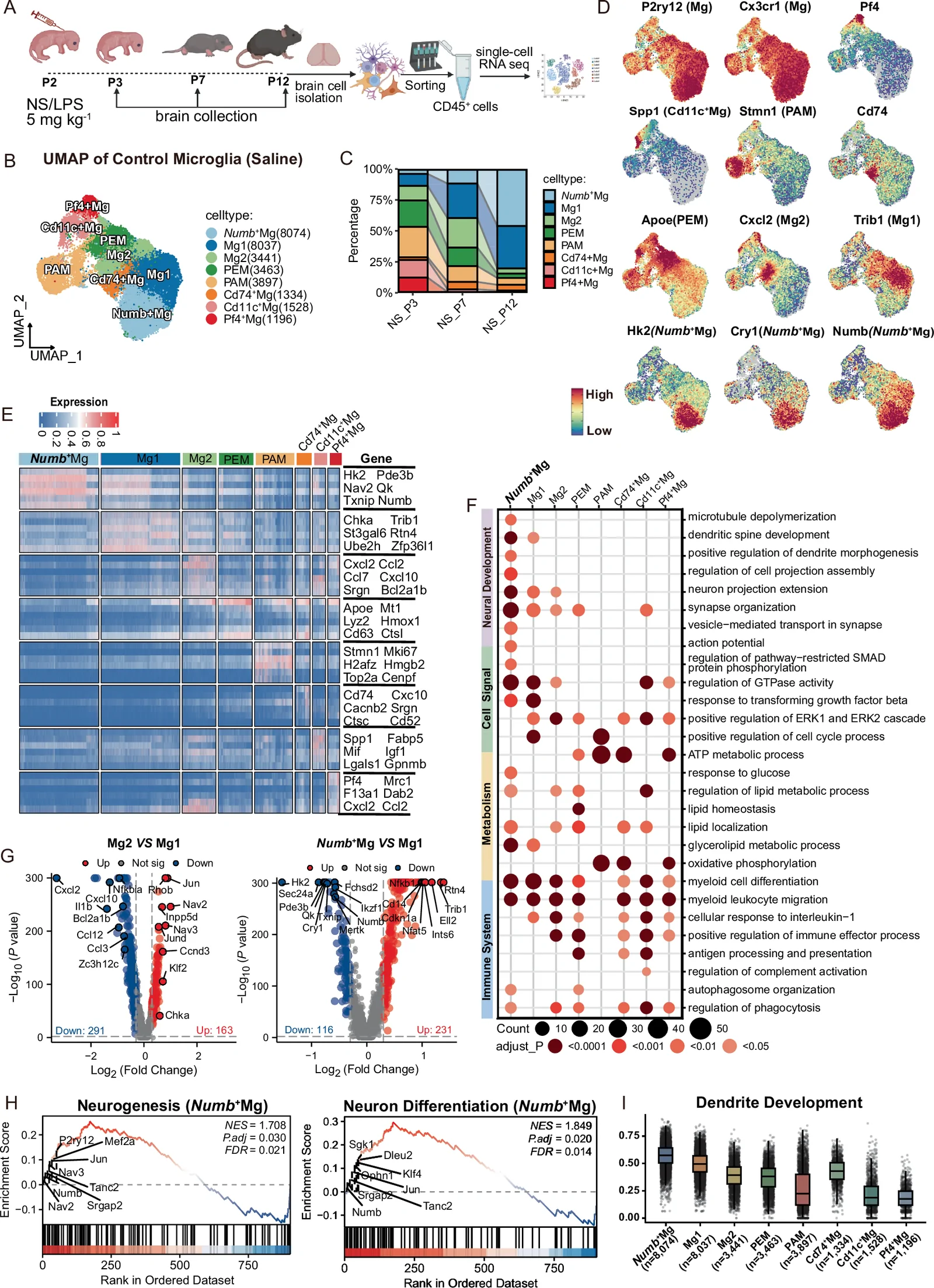
A time-resolved scRNA-seq atlas defines the physiological microglial landscape during early postnatal development. **A** Experimental workflow. Male mice received normal saline (NS) or lipopolysaccharide (LPS; 5 mg kg⁻¹) at P2. Brains were collected at P3, P7, and P12, and enriched CD45⁺ brain immune cells underwent scRNA-seq. Created in BioRender. Iu (2026) https://BioRender.com/1hxnvvdm. **B** UMAP of 30,970 microglia from seven independent male mice (P3, *n*  =  3; P7, *n*  =  2; P12, *n*  =  2), colored by microglial state. **C** Stacked bar plots showing the relative abundance of each state across postnatal stages. **D** Feature plots of representative marker genes; color indicates normalized expression. **E** Heatmap showing scaled expression of selected marker genes across microglial states. Columns represent cells and rows represent genes; expression was min–max normalized per gene to 0–1. **F** Bubble plot showing selected GO-BP terms enriched across microglial states. Dot size indicates gene count, and color indicates Benjamini–Hochberg-adjusted *p* value. Enrichment was assessed using a one-sided hypergeometric test. **G** Volcano plots showing differential expression between Mg2 and Mg1 and between *Numb*⁺Mg and Mg1. Two-sided Wilcoxon rank-sum tests with Bonferroni correction were used. Red and blue indicate significantly upregulated and downregulated genes, respectively (adjusted *p*  <  0.01 and |log₂ fold change| > 0.3). **H** GSEA based on differential-expression analysis, showing enrichment of neurogenesis and neuron-differentiation gene sets in *Numb*⁺Mg. *p* values were adjusted using the Benjamini–Hochberg method; red-to-blue shading indicates the ranked gene metric, black vertical lines indicate gene-set members, and the curve shows the running enrichment score. **I** UCell-derived dendrite-development scores across microglial states using the GO-BP gene set listed in Supplementary Data 2. Box plots show the median (central line), 25th and 75th percentiles (box limits), and whiskers extending to the smallest and largest values within 1.5 times the interquartile range. Gray points represent individual cells. Exact cell numbers are indicated in the panel; *n* denotes individual cells. *Numb*⁺Mg postnatal *Numb*-enriched microglial state, Mg microglia, PAM proliferation-associated microglia, PEM postnatal early microglia, UMAP uniform manifold approximation and projection, GO-BP Gene Ontology biological process, GSEA gene set enrichment analysis, NES normalized enrichment score, FDR false discovery rate.

To establish a physiological reference for interpreting neonatal inflammatory remodeling, we generated a time-resolved single-cell atlas of CD45⁺ brain immune cells from neonatal mice injected with normal saline (NS) or lipopolysaccharide (LPS) at P2 and profiled at P3, P7, and P12 using the SeekOne® platform. After quality control, the integrated dataset comprised 92,906 high-quality cells from 13 biologically independent male mice (Figs. 1A and S1A–C). We first analyzed the NS cohort to define the unperturbed trajectory of postnatal microglial maturation. Across seven NS samples, we identified 30,970 microglia based on canonical marker expression, including *P2ry12*, *Cx3cr1*, and *Hexb*^25^. Unsupervised clustering resolved eight transcriptionally distinct microglial clusters whose relative abundance changed markedly across P3, P7, and P12 (Fig. 1B–E), indicating that early postnatal microglia undergo substantial state remodeling rather than following a uniform maturation trajectory.

We annotated these microglia states by comparing their marker profiles with previously described developmental microglial populations^17,19^. This analysis identified Pf4⁺ microglia (*Pf4*, *Mrc1*, *Dab2*)^17^, Cd11c⁺Mg (*Spp1*, *Igf1*, *Gpnmb*)^20^, Cd74⁺Mg (*Cd74*, *Arg1*), and proliferation-associated microglia (PAM; *Mki67*, *Stmn1*, *H2afz*) under physiological conditions (Fig. 1B–E). These specialized and proliferative states showed distinct temporal distributions. Pf4⁺Mg and Cd11c⁺Mg were most abundant at P3 and declined sharply thereafter, Cd74⁺Mg remained rare throughout the period examined, and PAM progressively decreased from P3 to P12 (Supplementary Data 1). Thus, early postnatal development is accompanied by the contraction of several neonatal-enriched specialized and proliferative microglial states.

Among the P3-enriched clusters, we identified a distinct microglial state that we termed postnatal early microglia (PEM). PEM accounted for 21.4% of microglia at P3 but decreased to 3.4% by P12 (Fig. 1C and Supplementary Data 1). Although PEM shared lysosomal features with Cd11c⁺Mg, it showed limited expression of canonical Cd11c⁺Mg markers, including *Igf1*, *Gpnmb*, and *Spp1*, supporting its separation from this previously described state (Fig. 1D, E). Instead, PEM was characterized by genes involved in lipid handling (*Apoe*, *Lpl*, *Apoc1*), lysosomal function (*Ctsb*, *Ctsd*, *Cd63*), and oxidative-stress responses (*Mt1*, *Hmox1*, *Selenop*) (Fig. 1E, F). These features characterize PEM as a transient early postnatal microglial state enriched for lipid-handling, lysosomal, and oxidative-stress-response transcriptional programs, which is most abundant at P3 and largely diminishes by P12.

Mg2 showed a different temporal pattern, with a peak at P7, when it represented 23.8% of microglia, before declining to 4.2% by P12 (Supplementary Data 1). Mg2 expressed chemokine and cytokine genes, including *Cxcl2*, *Il1b*, *Ccl2*, *Ccl3*, *Ccl4*, and *Ccl7*, together with regulators linked to NF-κB, NFAT, and HIF-1α signaling, including *Zc3h12c*, *Rcan1*, *Nfat5*, *Bcl2a1b*, and *Cited2*^26^. GO analysis further showed enrichment of innate immune-response and leukocyte-migration pathways in Mg2 (Fig. 1F). Direct comparison with Mg1 confirmed preferential expression of inflammatory and immune-regulatory transcripts in Mg2 (Fig. 1G), consistent with a transient physiological microglial state characterized by immune-responsive transcriptional features during early postnatal development.

The final major trend was the progressive expansion of Mg1, which increased from 9.7% of microglia at P3 to 34.3% at P12 (Supplementary Data 1). Mg1 expressed canonical homeostatic microglial markers, including *P2ry12*, *Tmem119*, and *Fcrls*, together with genes associated with lipid and glycan processing (*Chka*, *Man1a1*, *St3gal6*)^27^ and protein quality control (*Edem1*). Compared with Mg2, Mg1 showed lower expression of inflammatory transcripts and higher expression of homeostasis-associated genes (Fig. 1G), consistent with the progressive acquisition of homeostatic features during early postnatal maturation.

Together, this time-resolved single-cell atlas provides a physiological reference for early postnatal microglial maturation and shows that this process involves dynamic changes in microglial state composition rather than a simple progression toward homeostasis. This framework provides the basis for identifying developmentally timed microglial states and their associated transcriptional programs, and for assessing how neonatal inflammation reshapes this organization.

### **A postnatal*Numb*-enriched microglial state is characterized by neurodevelopment-associated and metabolic transcriptional programs**

The physiological atlas further revealed that postnatal microglial maturation was not simply a linear progression toward homeostatic Mg1. Alongside the expansion of Mg1, we identified a second major state that increased from 4.0% of microglia at P3 to 11.8% at P7 and became the most abundant microglial state by P12, accounting for 46.0% of microglia (Fig. 1C and Supplementary Data 1). Although this state expanded over a developmental interval similar to that of Mg1, it remained transcriptionally distinct, indicating that postnatal microglial maturation involves parallel, molecularly distinguishable states rather than a uniform transition toward homeostasis. Following marker-based and context-specific nomenclature recommendations, we designated this state the postnatal *Numb*-enriched microglial state (*Numb*^+^Mg), based on its enrichment for *Numb* and its postnatal temporal profile^28^.

*Numb*^+^Mg displayed coordinated expression of genes linked to neurodevelopmental processes. Among its 30 most enriched genes, 16 (53.3%) were associated with developmental processes (Table 1), including oligodendrogenesis and myelin maintenance (*Qk*, *Mertk*)^29,30^, neural progenitor division (*Plxdc2*)^31^, axon guidance and neurite extension (*Numb*, *Nav2*, *Nav3*)^32–35^, and synaptic structure and engulfment (*Tanc2*, *Fchsd2*, *Elmo1*)^36,37^. Pathway-level analyses reinforced this transcriptional profile. GO terms were enriched for dendritic spine development and synaptic physiology (Fig. 1F), while gene set enrichment analysis identified neuronal differentiation (NES = 1.849, adjusted *p* = 0.020) and neurogenesis (NES = 1.708, adjusted *p* = 0.030) as programs associated with *Numb*^+^Mg (Fig. 1H). UCell scoring ranked *Numb*^+^Mg highest for dendritic-development signatures (Fig. 1I and Supplementary Data 2). Thus, although *Numb*^+^Mg is designated based on *Numb* enrichment, it is characterized by a broader neurodevelopment-related transcriptional signature associated with neuronal maturation, synaptic organization, and early circuit development.

**Table 1:** Functional annotation of the top 30 genes in the postnatal *Numb*-enriched microglial state

Category	Gene	Function/Description
Brain development	Mertk	A regulator of oligodendrogenesis and myelin ultrastructure^28^
	Numb	Regulates neurogenesis and neuronal differentiation^31,54^
	Nav3	Neuronal morphogenesis and neuromuscular responses^34^
	Nav2	Coordinated program of neuronal process outgrowth and migration resulting in a foliated structure^32,33^
	Qk	A regulator of oligodendrogenesis and myelin ultrastructure^29^
	Plxdc2	Regulates the division of neural progenitor cells^30^
	Zfhx3	Regulates neuronal development^56^
	Tanc2	Regulates synaptic structure and function^35,57^
	Srgap2	Regulates neuronal migration and differentiation^58,59^
	Epb41l2	Involved in regulating interactions within the axonal cytoskeleton and maintaining myelin structure^60,61^
	Elmo1	Regulates neuronal engulfment activity^62–64^
	Ikzf1(TF)	Regulates synaptic plasticity^65^
	Frmd4a	Involved in cell structure, transport, and signaling^66^
	Fchsd2	Regulates synaptic function^36,64^
	Cry1	Regulates circadian rhythm^67^
TF	Maml3	One of the elements of Notch signaling^68^
	Jun	Involved in cell proliferation and differentiation^69^
	Fos	Involved in cell proliferation and differentiation
Energy metabolism	Hk2	Rate-limiting enzyme in the glycolytic pathway
	Pde3b	Regulates the levels of cAMP and cGMP
	Txnip	Regulator of glucose and lipid metabolism^70,71^
	Inpp5d	Regulator of lipid metabolism
	Sec24a	Regulator of lipid metabolism
	Ldlrad4	Regulator of lipid metabolism^72,73^

*Numb*^+^Mg was also distinguished by a coordinated metabolic transcriptional program. It showed enrichment of genes associated with glycolysis (*Hk2*), cyclic nucleotide signaling (*Pde3b*)^38^, *redox and glucose regulation (Txnip)*^39^, and lipid trafficking or membrane-associated transport (*Inpp5d*, *Sec24a*, *Ldlrad4*). Direct comparison with Mg1 identified 231 genes upregulated in *Numb*^+^Mg, including both neurodevelopment-associated genes, such as *Tanc2* and *Numb*, and metabolic regulators, such as *Hk2* and *Txnip* (Fig. 1G). These transcriptional differences further distinguish Numb^+^Mg from Mg1 and characterize *Numb*^+^Mg as a postnatal microglial state enriched for both neurodevelopment-related and metabolic transcriptional programs. The negligible expression of neuronal and oligodendroglial lineage markers in *Numb*^+^Mg further argues against non-microglial contamination as the basis for its transcriptional profile (Fig. S1D).

Together, these analyses characterize *Numb*^+^Mg as a developmentally timed postnatal microglial state that expands in parallel with, but remains molecularly distinct from, homeostatic Mg1. Its combined neurodevelopment-related and metabolic transcriptional programs further indicate that postnatal microglial maturation involves parallel molecular states rather than a simple linear transition toward homeostasis.

### *Numb*^+^Mg is associated with a late pseudotemporal branch and is temporally restricted during postnatal development

**Fig. 2:**
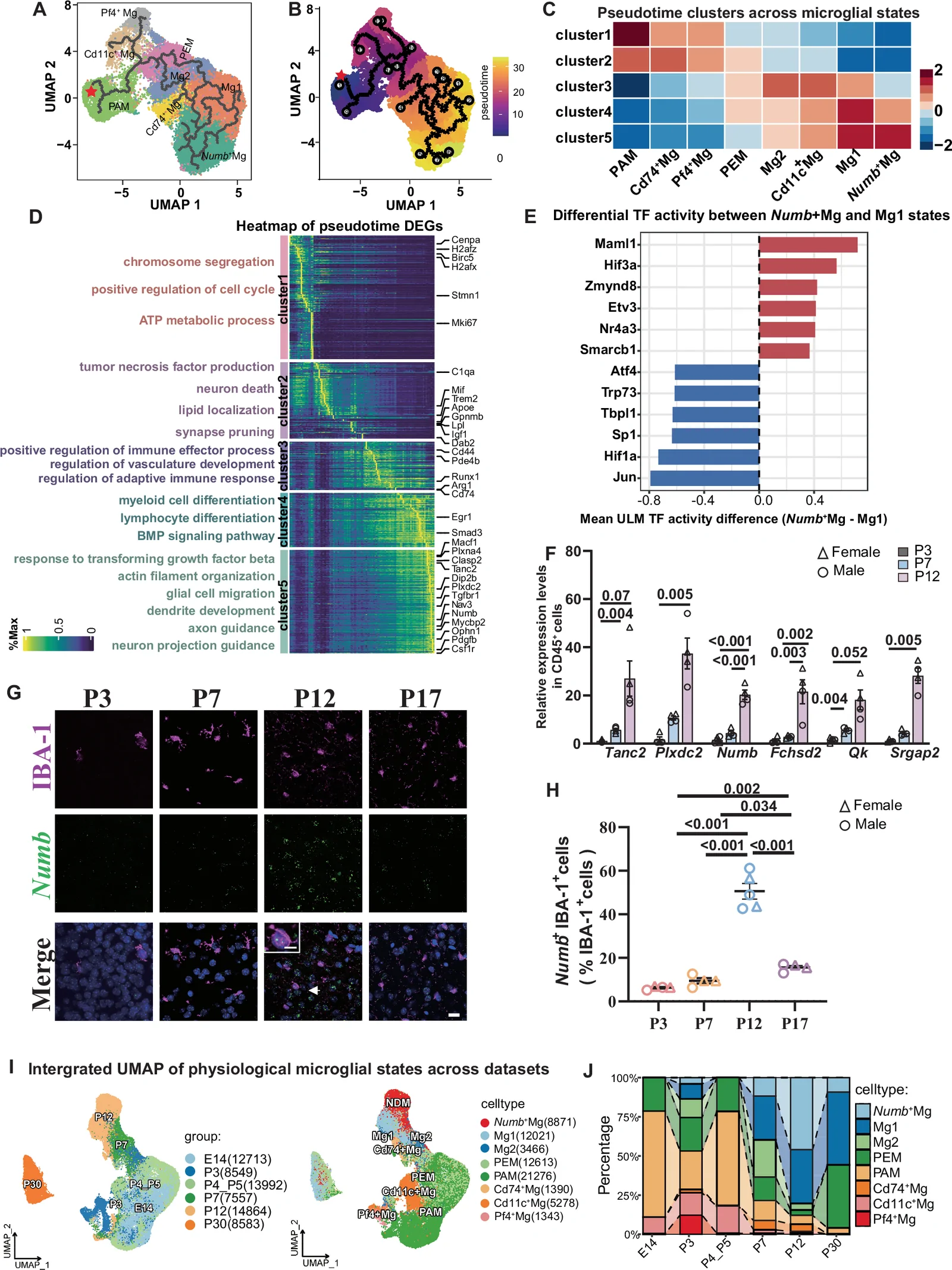
The postnatal *Numb*-enriched microglial state emerges along a late branch of early postnatal microglial maturation and is temporally restricted. **A**, **B** Pseudotemporal trajectory of NS microglia across P3–P12, rooted in proliferation-associated microglia (PAM). UMAPs show microglial states (**A**) or pseudotime (**B**; purple, early; yellow, late). Black lines indicate the principal graph, and the red star marks the root node. **C** Mean-scaled expression of pseudotime gene modules; Color indicates *z*-score-scaled expression. **D** Pseudotime-varying genes ordered along the trajectory (right) and enriched GO-BP terms for each co-expression module (left). Color indicates expression relative to each gene’s maximum. **E** ULM-Inferred TF activity difference between *Numb*⁺Mg and Mg1 (*Numb*⁺Mg − Mg1). Red and blue indicate higher activity in *Numb*⁺Mg and Mg1, respectively. **F** RT–qPCR validation of *Numb*⁺Mg-enriched markers in CD45⁺ cells from NS-treated mice. *n*  =  4 mice per stage. Data are mean ± SEM. *Numb* was analyzed by one-way ANOVA followed by Tukey’s post hoc test; *Tanc2*, *Plxdc2*, *Fchsd2*, *Qk*, and *Srgap2* were analyzed by Kruskal–Wallis *H* tests followed by Dunn’s post hoc tests. RNAscope images of *Numb* mRNA (green) and IBA-1 immunostaining (magenta) across stages (**G**), with quantification of *Numb*⁺IBA-1⁺ cells among IBA-1⁺ cells (**H**). Arrowheads indicate double-positive cells. Scale bars, 20 μm for main images and 5 μm for the magnified inset. P3, *n*  =  4; P7, *n*  =  4; P12, *n*  =  5; P17, *n*  =  4 mice. Data are mean ± SEM. Welch’s one-way ANOVA followed by Games–Howell’s post hoc test was used. **I** Integrated UMAP of microglia from this study and an external atlas containing E14, P4/P5, and P30 samples, colored by stage/source (left) or transferred state annotation (right). **J** Relative abundance of transferred states across stages. Triangles and circles indicate female and male mice, respectively. *Numb*⁺Mg postnatal *Numb*-enriched microglial state, GO-BP Gene Ontology biological process, Mg microglia, NS normal saline, RT–qPCR reverse-transcription quantitative PCR, TF transcription factor, ULM univariate linear model, UMAP uniform manifold approximation and projection. Source data are provided as a Source Data file.

Because *Numb*^+^Mg expanded in parallel with Mg1 while retaining a distinct transcriptional identity, we used Monocle 3 to reconstruct the pseudotemporal organization of NS microglia across P3–P12 and examine the relationship between these two states. We operationally rooted the trajectory in P3-enriched PAM and resolved two late transcriptional branches preferentially populated by Mg1 and *Numb*^+^Mg, respectively (Fig. 2A, B). Analysis of pseudotime-dependent genes identified five co-expression modules distributed along this inferred trajectory (Fig. 2C, D). Early pseudotime was enriched for a cell-cycle and ATP metabolism-associated module (Cluster 1), which was highest in PAM and Pf4⁺Mg and declined along the trajectory. Intermediate pseudotime was characterized by immune-related programs enriched across PEM, Mg2, Cd74⁺Mg, and Cd11c⁺Mg, including modules associated with tumor necrosis factor production and synapse-pruning-related signatures (Cluster 2), regulation of immune effector responses (Cluster 3), and myeloid/lymphocyte differentiation and BMP signaling (Cluster 4). By contrast, late pseudotime was marked by preferential enrichment of a cytoskeletal, morphogenetic, and synapse-related module (Cluster 5), with the strongest signal along the branch associated with *Numb*^+^Mg.

Inferred transcription-factor activity further distinguished the branches associated with Mg1 and *Numb*^+^Mg. *Maml1, Hif3a*, and *Zmynd8* activity was higher in *Numb*^+^Mg, whereas *Jun, Hif1a*, and *Sp1* activity was higher in Mg1 (Figs. 2E and S2A–C), identifying candidate regulatory configurations associated with the transcriptional divergence between *Numb*^+^Mg and homeostatic Mg1.

We next examined whether the neurodevelopment-related transcriptional features of *Numb*^+^Mg increased across chronological postnatal development. Across the sampled P3–P12 stages, dendrite-development UCell scores increased in parallel with the stage-dependent expression of representative genes enriched in *Numb*^+^Mg, with the highest levels observed at P12 among the stages examined (Fig. S2D, E). RT–qPCR analysis of the predominantly microglial CD45⁺ brain immune compartment further supported higher expression at P12 of representative *Numb*^+^Mg-associated genes, including *Tanc2, Plxdc2, Numb, Fchsd2, Qk*, and *Srgap2*, relative to earlier postnatal stages (Figs. 2F and S1C).

To assess the temporal pattern of a representative *Numb*^+^Mg-associated marker in situ, we performed RNAscope for *Numb* transcripts in IBA-1⁺ cells across P3, P7, P12, and P17. The proportion of *Numb-*expressing IBA-1*⁺*cells remained low at P3 and P7 (6.3 ± 0.4% and 9.5 ± 1.3%, respectively), increased sharply at P12 (50.6 ± 3.6%), and declined by P17 (15.6 ± 0.9%; Fig. 2G, H). This temporal pattern supports a restricted postnatal increase in *Numb*^*+*^ microglia and is consistent with the dynamics of *Numb*^+^Mg observed in the single-cell RNA-seq atlas.

We further assessed whether this postnatal enrichment persisted at later developmental stages by projecting our microglial states onto a published developmental atlas spanning E14.5, P4/P5, and P30 (Figs. 2I, J and S2F, G)^17^. Cells assigned to *Numb*^+^Mg preferentially aligned with the early postnatal P4/P5 compartment, showed minimal alignment with embryonic E14.5 microglia, and were only sparsely represented at P30. By contrast, P30 microglia mapped predominantly to Mg1-like or homeostatic states. These cross-atlas projections further support the preferential association of *Numb*^+^Mg with postnatal development and its marked reduction by P30.

Together, these analyses identify *Numb*^+^Mg as a temporally restricted postnatal microglial state associated with a late pseudotemporal branch, supporting a branched transcriptional organization of postnatal microglial maturation, rather than a simple linear progression toward homeostatic identity.

### Early postnatal microglial depletion disrupts cortical maturation and later neurobehavioral outcomes

**Fig. 3:**
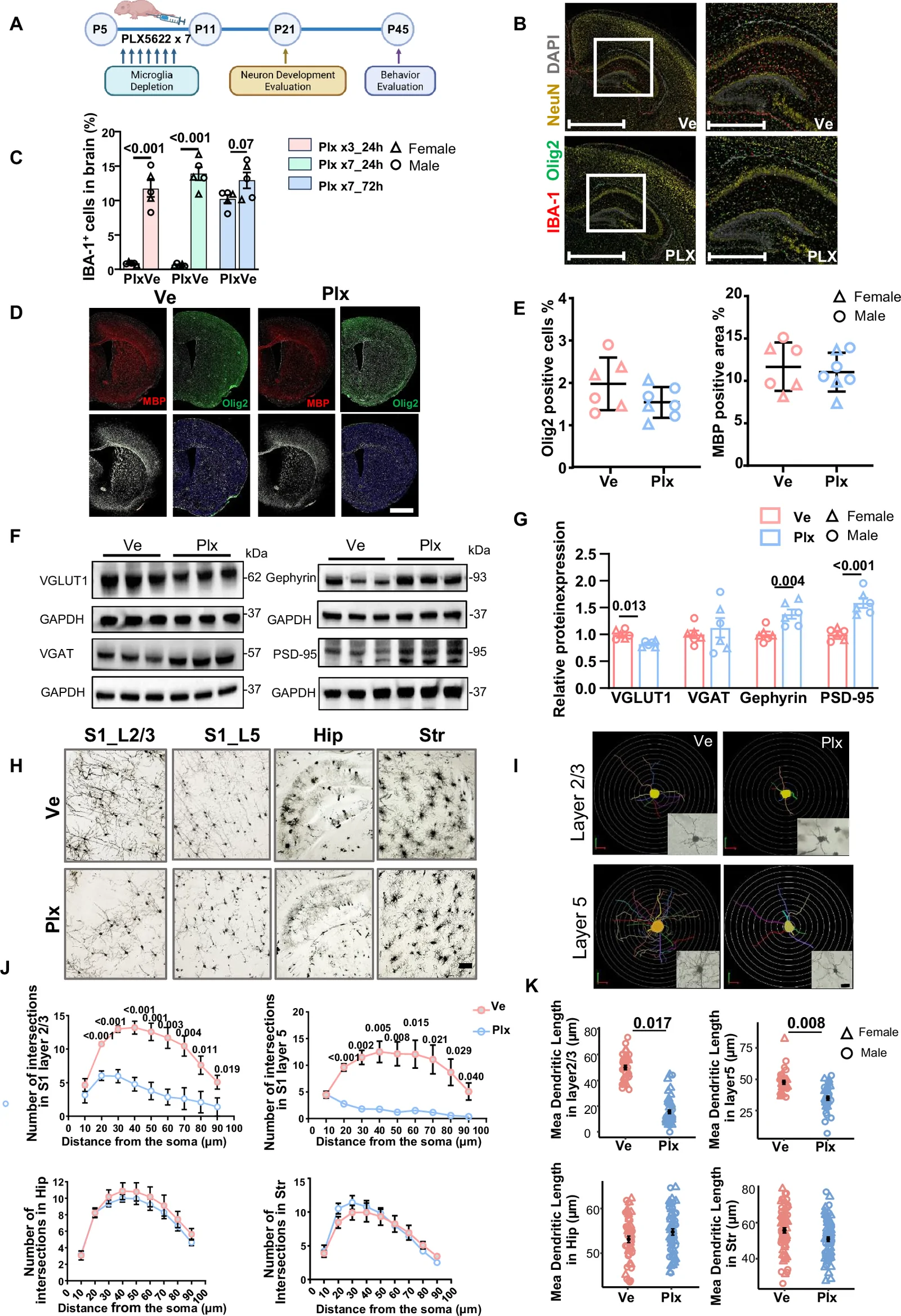
Early postnatal microglial depletion spares gross myelination but alters synaptic protein composition and disrupts S1 dendritic maturation. **A** Experimental design. PLX5622 was administered from P5 to P11, followed by assessment at P21 and P45. Created in BioRender. Iu (2026) https://BioRender.com/12qf67u. **B** Representative immunofluorescence images IBA-1, Olig2, NeuN, and DAPI in vehicle-treated (Ve) and PLX5622-treated (Plx) mice 24 h after the third injection. Scale bars, 1 mm and 500 μm for main and magnified images, respectively. **C** Quantification of IBA-1⁺ cells after 3 or 7 days of treatment or 72 h after withdrawal. *n*  =  5 mice per group. Two-sided Welch’s *t*-tests were used for Plx×3_24h and Plx×7_24h, and a two-sided unpaired Student’s *t* test for Plx×7_72h. Representative images of MBP, Olig2, and DAPI at P21 (**D**) and quantification of Olig2⁺ cells and MBP⁺ area (**E**). Scale bars, 1 mm. Ve, *n*  =  6 mice; Plx, *n*  =  7 mice. Two-sided unpaired Student’s *t* tests were used. Western blot analysis (**F**) and quantification (**G**) of synaptic proteins in lysates at P21. *n*  =  6 mice per group. Two-sided unpaired Student’s *t* tests were used for VGLUT1, Gephyrin, and PSD-95, and a two-sided Welch’s *t*-test for VGAT. **H** Representative Golgi–Cox-stained images of neurons in the primary somatosensory cortex (S1) layers 2/3 and 5, hippocampus (Hip), and striatum (Str) at P21. Scale bars, 200 μm. Representative neuronal reconstructions (**I**), Sholl analysis (**J**), and dendritic-length quantification (**K**). Scale bars, 25 μm. *n*  =  5 mice per group. Measurements from multiple neurons were averaged within each mouse. Mouse-level averages were used to calculate mean ± SEM and for statistical analysis; individual neuron measurements are overlaid in **K** for visualization only. Sholl data were analyzed using two-sided Welch’s *t*-tests at each distance. Dendritic length was analyzed using a two-sided Wilcoxon rank-sum test for S1 layer 5 and two-sided unpaired Student’s *t* tests for S1 layer 2/3, hippocampus, and striatum. Data in **C**, **E**, G are mean ± SEM. Triangles and circles indicate female and male mice, respectively. Uncropped and unprocessed blot scans and source data are provided in the Source Data file.

The neurodevelopment-associated transcriptional programs and temporally restricted expansion of *Numb*^+^Mg suggested that microglia during this developmental period may contribute to early postnatal neural maturation. We therefore administered the CSF1R inhibitor PLX5622 from P5 to P11, initiating treatment before the rapid expansion of *Numb*^+^Mg and Mg1 and achieving robust microglial depletion during the interval leading to prominent *Numb*^+^Mg enrichment at P12 (Fig. 3A). Quantification of IBA-1^+^ cells confirmed efficient depletion after 3 days of treatment, which was maintained through 7 days of treatment. Following PLX5622 withdrawal, microglia repopulated rapidly and were largely restored within 72 h (Fig. 3B, C).

We first examined myelination, a major developmental process occurring during this early postnatal interval and one previously linked to specialized neonatal microglial states. At P21, PLX5622 treatment did not significantly alter MBP-positive area or Olig2^+^ oligodendrocyte-lineage cell density, indicating that gross myelin development was not measurably impaired under these conditions (Fig. 3D, E). Given that this depletion window overlapped with the period of *Numb*^+^Mg enrichment and that this state was characterized by synapse- and dendrite-related transcriptional programs, we next performed an exploratory whole-brain protein-level screen for synapse-associated markers. PLX5622-treated mice showed increased Gephyrin and PSD-95 protein levels, reduced levels of the excitatory presynaptic marker VGLUT1, and unchanged levels of the inhibitory presynaptic marker VGAT (Fig. 3F, G).

**Fig. 4:**
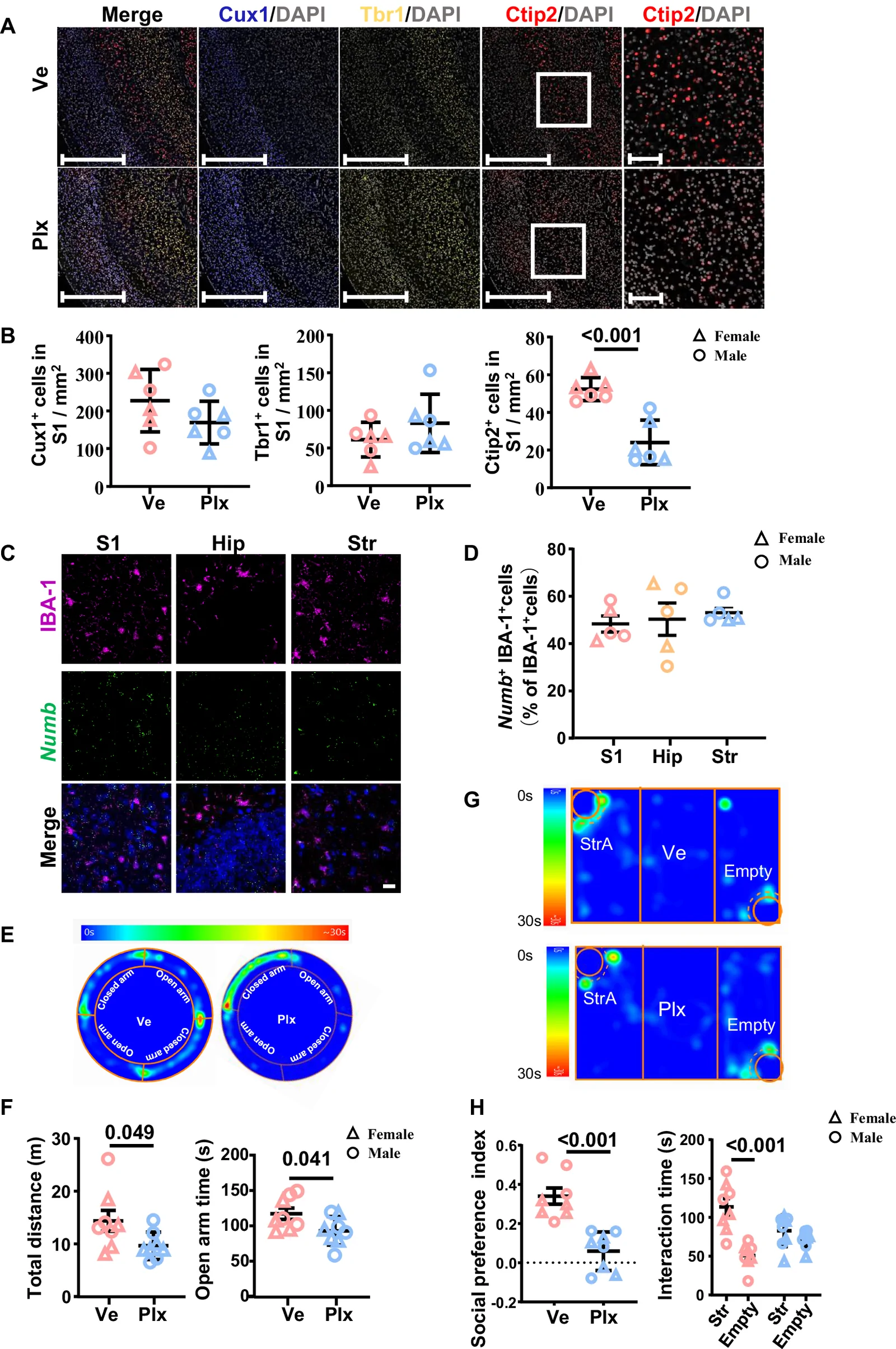
Early postnatal microglial depletion reduces Ctip2⁺ layer 5 neurons in S1 and is associated with later behavioral alterations. Representative immunofluorescence images (**A**) and quantification (**B**) of Cux1⁺, Tbr1⁺, and Ctip2⁺ neurons in the primary somatosensory cortex (S1) at P21. Scale bars, 500 μm for the main images and 100 μm for the magnified images. *n*  =  6 mice per group. Data were analyzed using two-sided unpaired Student’s *t* tests. Representative RNAscope images of *Numb* mRNA (green) and IBA-1 immunostaining (magenta) in S1, hippocampus (Hip), and striatum (Str) at P12 (**C**), with quantification of *Numb*⁺IBA-1⁺ cells among total IBA-1⁺ cells in each region (**D**). Scale bars, 20 μm. *n*  =  5 mice. Data were analyzed using a Kruskal–Wallis *H* test. Representative elevated zero-maze tracking heatmaps (**E**) and quantification of total distance traveled and open-arm time (**F**) at P43. *n*  =  8 mice per group. Data were analyzed using two-sided unpaired Student’s *t* tests. Representative three-chamber tracking heatmaps (**G**) and quantification of social preference index and interaction time with the unfamiliar mouse or empty target (**H**) at P45. *n*  =  8 mice per group. Data were analyzed using two-sided unpaired Student’s *t* tests. In **E** and **G**, color indicates occupancy time. All quantitative data are presented as mean ± SEM. Triangles and circles indicate female and male mice, respectively. Ve vehicle, Plx PLX5622; Source data are provided as a Source Data file.

To determine whether these synapse-associated changes were accompanied by neuronal structural alterations, we examined neuronal architecture across selected brain regions. Golgi–Cox staining, neuronal reconstruction, and Sholl analysis revealed reduced dendritic complexity and mean dendritic length in the primary somatosensory cortex (S1), affecting both layer 2/3 and layer 5 neurons. In contrast, hippocampal and striatal neurons showed no detectable dendritic alterations under the same conditions (Fig. 3H–K), indicating that S1 neuronal architecture was particularly sensitive to early postnatal microglial depletion among the regions examined. Consistent with this S1-predominant structural phenotype, layer-specific immunofluorescence analysis showed a significant reduction in Ctip2⁺ layer 5 projection neurons, whereas Cux1⁺ layer 2/3 and Tbr1⁺ layer 6 neuronal populations were not significantly changed (Fig. 4A, B).

We next asked whether this preferential S1 phenotype was associated with regional enrichment of *Numb*-expressing microglia, a representative in situ feature of *Numb*^+^Mg. RNAscope analysis at P12 showed that *Numb*⁺IBA-1⁺ cells were present across S1, hippocampus, and striatum, with no significant regional differences (Fig. 4C, D). Thus, the S1-predominant structural vulnerability was not accompanied by regional accumulation of *Numb*⁺IBA-1⁺ microglia, arguing against regional enrichment of *Numb*-expressing microglia as a simple explanation for this phenotype.

We further assessed whether microglial depletion during this early postnatal window was associated with later behavioral alterations. At P45, PLX5622-treated mice showed reduced total distance traveled and decreased open-arm time in the elevated zero maze (Fig. 4E, F), indicating altered exploratory performance in the context of reduced locomotor activity. In the three-chamber test, PLX5622-treated mice spent less time interacting with the unfamiliar mouse and showed a reduced social preference index compared with controls (Fig. 4G, H). Together, these findings indicate that depletion of microglia during this early postnatal window spares gross myelination but disrupts S1 neuronal maturation and is associated with subsequent neurobehavioral alterations.

### Neonatal inflammation remodels the microglial landscape at P12 and reduces *Numb*^+^Mg representation

**Fig. 5:**
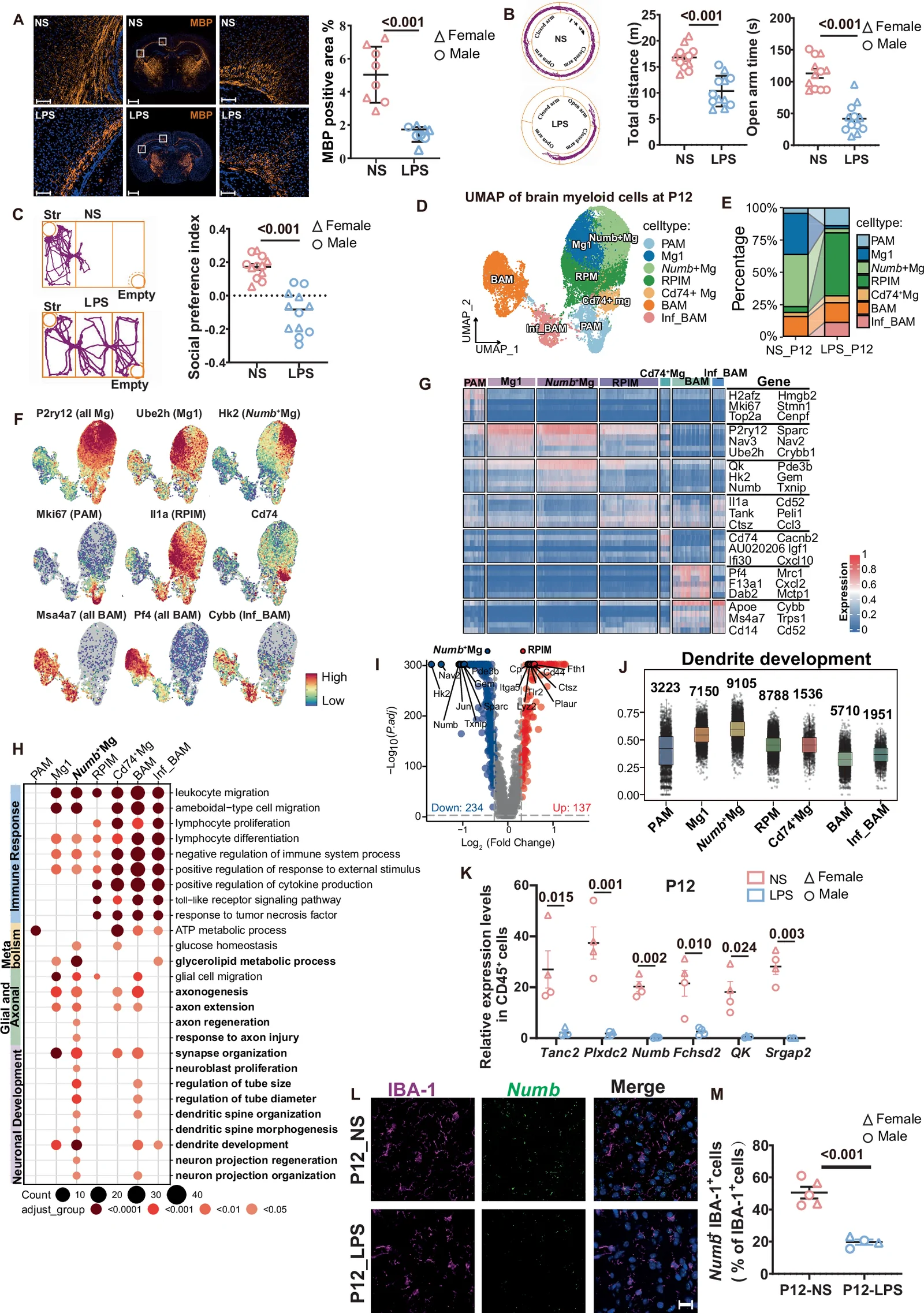
Neonatal LPS suppresses developmental programs of the postnatal *Numb*-enriched microglial state and promotes inflammatory microglial states at P12. **A** Representative MBP immunofluorescence image and corresponding quantification in NS- and LPS-treated mice at P12. Scale bars, 1 mm and 100 μm for main and magnified images; *n*  =  8 mice per group. Two-sided Welch’s *t*-test. **B** Elevated zero-maze tracking, total distance, and open-arm time at P43; *n*  =  12 mice per group. Color indicates occupancy time. Total distance was analyzed using a two-sided unpaired Student’s *t* test, and open-arm time was analyzed using a two-sided Welch’s *t*-test. **C** Three-chamber tracking and social preference at P45; *n*  =  12 mice per group. Two-sided Welch’s *t*-test. **D** UMAP of 37,463 microglia and BAMs from two male mice per group. **E** Relative abundance of annotated states. **F** Marker-gene feature plots. **G** Marker-gene heatmap; columns are cells and rows are genes, with expression min–max normalized per gene to 0–1. **H** GO-BP enrichment. Dot size indicates gene count, and color indicates Benjamini–Hochberg-adjusted *p* value (one-sided hypergeometric test). **I** Differential expression between *Numb*⁺ Mg and RPIM. Two-sided Wilcoxon rank-sum tests with Bonferroni correction; red and blue indicate upregulated and downregulated genes (adjusted *p*  <  0.01; |log₂ fold change| > 0.3). **J** UCell dendrite-development scores. Box plots show the median, 25th and 75th percentiles, and whiskers extending to the most extreme values within 1.5 times the interquartile range. Gray points are cells. Numbers above boxes indicate cells analyzed; *n* denotes cells. **K** RT–qPCR of *Numb*⁺Mg-associated genes in CD45⁺ cells; *n*  =  4 mice per group. Two-sided unpaired Student’s *t* tests or Welch’s *t*-tests were used as appropriate. RNAscope images of *Numb* mRNA and IBA-1 immunostaining (**L**) and quantification of *Numb*⁺IBA-1⁺ cells among IBA-1⁺ cells (**M**). Scale bars, 20 μm. NS, *n*  =  5 mice; LPS, *n*  =  4 mice; two-sided Welch’s *t*-test. Quantitative data in **A**–**C**, **K**, **M** are mean ± SEM. Triangles denote females; circles, males. *Numb*⁺Mg postnatal *Numb*-enriched microglial state, BAM border-associated macrophages, Inf_BAM inflammation-associated BAMs, RPIM recovery-phase inflammatory microglia, GO-BP Gene Ontology biological process, NS normal saline, LPS lipopolysaccharide, UMAP uniform manifold approximation and projection. Source data are provided as a Source Data file.

The finding that early postnatal microglial depletion disrupted neural maturation raised the question of whether neonatal inflammation interferes with the establishment of microglial states that normally emerge during this developmental period. To model neonatal inflammation-associated injury in the immature brain, mice received a single subcutaneous dose of lipopolysaccharide (LPS) at P2, a developmental stage overlapping early microglial expansion and maturation^40–42^. This challenge was associated with reduced MBP-positive area at P12, altered S1 dendritic morphology at P21, and behavioral alterations at P45 (Figs. 5A–C and S5A, B). We therefore focused on P12, when *Numb*^+^Mg is most prominent under physiological conditions, to examine whether neonatal inflammation remodels microglial state architecture and attenuates the establishment of this state.

To contextualize parenchymal microglial remodeling relative to macrophage-associated inflammatory signatures, we analyzed P12 microglia together with border-associated macrophage (BAM) and inflammatory BAM-like (Inf_BAM) populations. Integrated analysis identified five principal parenchymal microglial states—*Numb*^+^Mg, Mg1, PAM, Cd74^+^Mg, and a prominent LPS-associated recovery-phase inflammatory microglial state, termed RPIM—alongside BAM and Inf_BAM populations (Fig. 5C–F and Supplementary Data 3). Markers enriched in *Numb*^+^Mg, including *Hk2, Numb*, and *Txnip*, segregated from BAM-associated macrophage genes such as *Pf4, F13a1*, and *Ms4a7*, supporting separation of the transcriptional signature associated with *Numb*^+^Mg from macrophage-associated signatures within the inflamed neonatal brain (Figs. 5F, G and S3A).

Comparison of parenchymal microglial state composition revealed a marked shift after neonatal LPS exposure. Under physiological conditions at P12, *Numb*^+^Mg and Mg1 dominated the parenchymal microglial compartment, accounting for approximately 40% and 32% of cells, respectively (Supplementary Data 3). After LPS exposure, the relative abundance of *Numb*^+^Mg decreased to approximately 3% and that of Mg1 to approximately 2%, whereas RPIM increased to approximately 49% of parenchymal microglia (Fig. 5E). RPIM was marked by inflammatory and immunometabolic programs, including pattern-recognition and NF-κB-associated regulators (*Peli1, Tank*), cytokine and chemokine genes (*Il1a, Ccl3*), activation-associated surface molecules (*Cd52*), and phagolysosomal components (*Ctsz*) (Fig. 5G–I). By contrast, *Numb*^+^Mg retained developmental and metabolic features characteristic of physiological *Numb*^+^Mg at P12. Pathway analysis and UCell scoring reinforced this separation, with RPIM enriched for immune-activation pathways and *Numb*^+^Mg showing stronger neurite-, synapse- and dendrite-development programs (Fig. 5H–J).

Having observed reduced representation of *Numb*^+^Mg at P12, we next examined whether transcripts enriched in this state were also attenuated. In the P12 single-cell dataset, microglia from LPS-exposed brains showed lower expression of representative neurodevelopmental and metabolic genes enriched in *Numb*^+^Mg compared with NS controls (Fig. S3B). RT–qPCR analysis of CD45⁺ brain immune cells across P3, P7, and P12 further showed that selected markers of *Numb*^+^Mg, including *Tanc2, Plxdc2, Numb, Fchsd2, Qk*, and *Srgap2*, were significantly reduced at P7 and P12 after LPS exposure (Figs. 5K and S3C). These data indicate that neonatal inflammation attenuates transcriptional features associated with *Numb*^+^Mg during the developmental interval in which this state normally becomes prominent.

Histological analyses further supported disruption of homeostatic and *Numb*^+^Mg-associated microglial features at the tissue level. At P12, LPS-exposed brains showed reduced P2RY12⁺ microglial area and cell density compared with NS controls, consistent with attenuation of homeostatic microglial features. In contrast, total IBA-1⁺ cell density did not differ significantly between groups, indicating that reduced representation of *Numb*^+^Mg was not simply attributable to global loss or expansion of the IBA-1⁺ microglial compartment (Fig. S4A–C). RNAscope analysis further showed a marked reduction in the proportion of *Numb*⁺IBA-1⁺ cells in LPS-exposed brains compared with NS controls at P12 (NS, 50.6 ± 3.6%; LPS, 19.7 ± 1.6%; Fig. 5L, M). Together, these single-cell, transcriptional, and tissue-level analyses show that neonatal inflammation redirects the P12 microglial landscape from a physiological organization dominated by *Numb*^+^Mg/Mg1 toward an RPIM-dominant inflammatory state with attenuated homeostatic and *Numb*^+^Mg-associated transcriptional features.

### Neonatal inflammation is associated with a temporally ordered shift from APIM- to RPIM-predominant microglial states

**Fig. 6:**
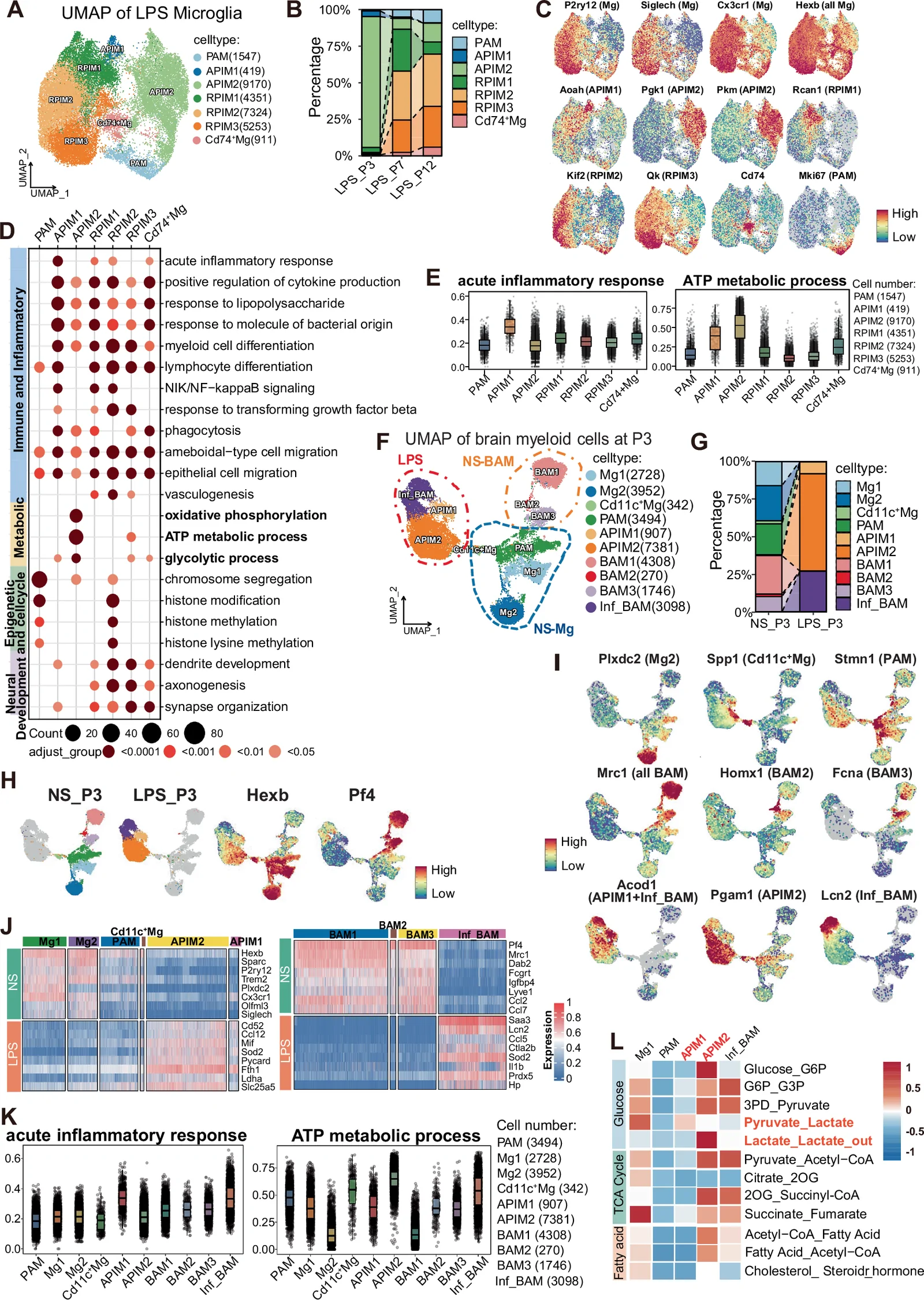
Neonatal inflammation induces time-dependent post-inflammatory microglial remodeling and early metabolic reprogramming. **A** UMAP of 28,975 microglia from six LPS-treated male mice at P3, P7, and P12 (*n*  =  2 mice per stage), colored by annotated microglial state. **B** Relative abundance of microglial states across postnatal stages after LPS exposure. **C** Feature plots of representative marker genes; color indicates normalized expression. **D** Selected GO-BP terms enriched across LPS-associated microglial states. Dot size indicates gene count, and color indicates Benjamini–Hochberg-adjusted *p* value. Enrichment was assessed using a one-sided hypergeometric test. **E** UCell-derived acute-inflammatory-response and ATP-metabolic-process scores across microglial states. **F** UMAP of 28,226 microglia and BAMs from three NS-treated and two LPS-treated male mice at P3, colored by cell state. Red, orange, and blue dashed outlines indicate LPS-associated cells, NS-associated BAMs, and NS-associated microglia, respectively. **G** Relative abundance of microglial and BAM-related states in NS and LPS groups. **H** UMAPs split by condition, with feature plots of representative identity markers. **I** Feature plots of markers distinguishing physiological microglial, APIM, and BAM-related states; color indicates normalized expression. **J** Heatmap of selected marker genes across annotated states. Columns represent cells and rows represent genes; expression was min–max normalized per gene to 0–1. **K** UCell-derived acute-inflammatory-response and ATP-metabolic-process scores across annotated states at P3. For **E** and **K**, box plots show the median (center line), 25th and 75th percentiles (box limits), and whiskers extending to the most extreme values within 1.5 times the interquartile range. Gray points represent individual cells; cell numbers are indicated in the panels, and *n* denotes cells. **L** Predicted metabolic fluxes in glycolysis, the tricarboxylic acid cycle, and fatty-acid metabolism inferred using scFEA; color indicates scaled relative flux. Mg microglia, PAM proliferation-associated microglia, APIM acute post-inflammatory microglia, RPIM recovery-phase inflammatory microglia, BAM border-associated macrophages, Inf_BAM inflammation-associated BAMs, GO-BP Gene Ontology biological process, NS normal saline, LPS lipopolysaccharide, TCA tricarboxylic acid, scFEA single-cell flux estimation analysis, UMAP uniform manifold approximation and projection.

The RPIM-dominant P12 landscape after LPS indicated that neonatal inflammation did not merely reduce *Numb*^+^Mg at a single endpoint, but may have redirected microglial maturation before this state became fully established. To test this, we analyzed LPS-exposed parenchymal microglia across P3, P7, and P12. This time-resolved analysis revealed a sequential pattern of post-inflammatory state remodeling in which acute post-inflammatory microglial states, APIM1 and APIM2, dominated the microglial response at P3, whereas recovery-phase inflammatory microglia, RPIM1–3, emerged from P7 onward and became predominant by P12 (Fig. 6A, B). These states retained microglial identity-associated genes, including *Hexb* and *Cx3cr1*, while remaining distinct from BAM-associated and peripheral immune-cell signatures (Fig. S5C–G and Supplementary Data 4)^25^, supporting their annotation as remodeled microglial states rather than macrophage or infiltrating immune-cell populations.

At P3, the acute response to neonatal LPS was dominated by APIM2, which accounted for approximately 90% of parenchymal microglia, whereas APIM1 represented a smaller population of approximately 4% (Fig. 6B and Supplementary Data 5). APIM2 displayed a metabolically activated profile, with enrichment of glycolysis- and ATP metabolism-associated pathways and expression of metabolic genes such as *Pgk1* and *Pkm* (Fig. 6C–E). In contrast, APIM1 showed a cytokine-rich inflammatory profile marked by *Il1b*, *Tnf*, and *Cxcl2*, together with enrichment of NF-κB signaling, chemotaxis, and cytokine-response pathways. Thus, the earliest microglial response to neonatal LPS was not organized as a uniform classical inflammatory state, but was dominated by APIM2-associated immunometabolic remodeling, with a smaller APIM1 compartment showing overt inflammatory activation.

This APIM-dominated acute response was followed by the emergence of recovery-phase inflammatory states from P7 onward. RPIM1 peaked at P7 and combined residual inflammatory mediators, including *Cxcl2* and *Il1b*, with regulatory factors such as *Zc3h12c* and *Nfat5*, consistent with a resolution-associated transitional profile. RPIM2 persisted across P7 and P12 and was enriched for transcriptional and chromatin-remodeling features, including *Jmjd1c*, *Klf6*, *Jun*, *Runx1*, and histone-modification pathways. By P12, RPIM3 became more prominent and re-expressed selected neurodevelopment-associated transcripts, including *Qk, Plxdc2*, and *Tanc2*, suggesting partial re-engagement of developmental transcriptional programs during recovery. However, compared with physiological *Numb*^+^Mg, RPIM3 showed incomplete restoration of *Numb*^+^Mg-associated signatures and reduced P2ry12 expression (Figs. 5F, I and 6C, D, S4B). Thus, neonatal inflammation progressed from acute APIM-associated immunometabolic remodeling to recovery-phase RPIM states that partially re-engaged developmental features without restoring the full transcriptional profile associated with *Numb*^+^Mg.

Consistent with this state-resolved temporal sequence, population-level analysis of all LPS-exposed parenchymal microglia revealed a coordinated temporal shift in dominant transcriptional programs (Supplementary Data 6). At P3, protein–protein interaction analysis highlighted metabolic and cytoskeletal activation, with modules enriched for mitochondrial ATP synthesis and oxidative phosphorylation genes, including *Atp5* and *Nduf* family members, glycolytic genes (*Pgk1*, *Pkm*), and cytoskeletal remodeling genes (*Rac2*, *Tubb5*) (Fig. S6A–C). By P12, the dominant programs had shifted toward immunoregulatory and tissue-remodeling pathways, including leukocyte migration, cytokine signaling, and extracellular matrix organization (Fig. S7A, B). Together, these analyses show that neonatal inflammation redirects microglia through a temporally ordered state-remodeling sequence, beginning with early metabolic and cytoskeletal activation and progressing toward recovery-phase immune-regulatory and tissue-remodeling states, rather than converging on the physiological Mg1/*Numb*^+^Mg state organization.

### APIM2 marks an early glycolytic remodeling phase preceding inflammation-induced *Numb*^+^Mg attenuation

The APIM-to-RPIM temporal pattern suggested that the attenuation of *Numb*^+^Mg at P12 was preceded by an earlier phase of inflammation-induced microglial remodeling. We therefore focused on P3, the earliest post-LPS time point in our atlas, when *Numb*^+^Mg is not yet prominent under physiological conditions, but inflammation-induced microglial deviation can already be detected. Because neonatal LPS also reshaped BAM-related macrophage compartments at this stage, we analyzed these populations alongside parenchymal microglia to define the compartment-specific organization of the early brain myeloid response.

Under NS conditions at P3, microglia and BAMs occupied clearly separated transcriptional compartments, with microglia distributed mainly across physiological early postnatal states, including Mg1, Mg2, Cd11c^+^Mg, and PAM. Neonatal LPS exposure rapidly reorganized this landscape: LPS-derived cells shifted away from regions occupied predominantly by physiological microglial states and were enriched in the APIM1, APIM2, and Inf_BAM regions in UMAP space (Figs. 6F, G, S8A and Supplementary Data 7). APIM1 and APIM2 retained *Hexb* expression and remained separated from *Pf4*^+^ BAM-related populations, supporting their annotation as inflammation-remodeled microglia rather than BAM-derived cells (Fig. 6H, J). Among these induced states, APIM2 was distinguished by metabolic genes such as *Pgam1*, whereas APIM1 was enriched for inflammatory-response genes, and Inf_BAM expressed BAM-associated inflammatory markers such as *Lcn2* (Fig. 6I, J).

This early P3 landscape, therefore, separated along metabolic and inflammatory axes. UCell scoring showed that ATP metabolism-associated signatures were highest in APIM2, whereas acute inflammatory-response scores were higher in APIM1 and Inf_BAM (Fig. 6K). scFEA-based flux inference further predicted APIM2 to have the highest glycolytic flux among the states examined, with higher predicted glucose-to-pyruvate conversion, pyruvate-to-lactate flux, and lactate export (Fig. 6L)^43^. This inferred flux pattern supports a Warburg-like glycolytic configuration in APIM2 during the acute response, in contrast to the weaker glycolysis-lactate module activity observed in APIM1 and Inf_BAM.

### APIM2 diversifies along an inflammatory axis while maintaining glycolytic features

Having identified APIM2 as the dominant glycolytic microglial state at the earliest stage of LPS-induced remodeling, we next asked whether this state was transcriptionally uniform or contained internal structure. High-resolution subclustering resolved APIM2 into five subclusters, APIM2a–e, which were derived predominantly from LPS-exposed brains and arranged along a continuous transcriptional gradient rather than forming sharply discrete populations (Fig. S8B–D and Supplementary Data 8). This gradient extended from proliferation-, translation-, and glycolysis-associated features toward stress-response, chemokine, and interferon-related programs. Representative genes included glycolytic regulators such as *Pgk1* and *Pgam1*, stress-response genes such as *Sod2*, chemokine genes such as *Ccl12*, and interferon-associated genes such as *Ifit3* (Figs. S8E and S9A). GO enrichment and UCell scoring further showed that ATP metabolism-associated signatures remained broadly elevated across APIM2 substates, whereas inflammatory-response programs increased along the gradient (Fig. S9B, C). Thus, APIM2 retained glycolytic and ATP metabolism-associated features while diversifying toward inflammatory and antiviral programs during the acute post-LPS response.

**Fig. 7:**
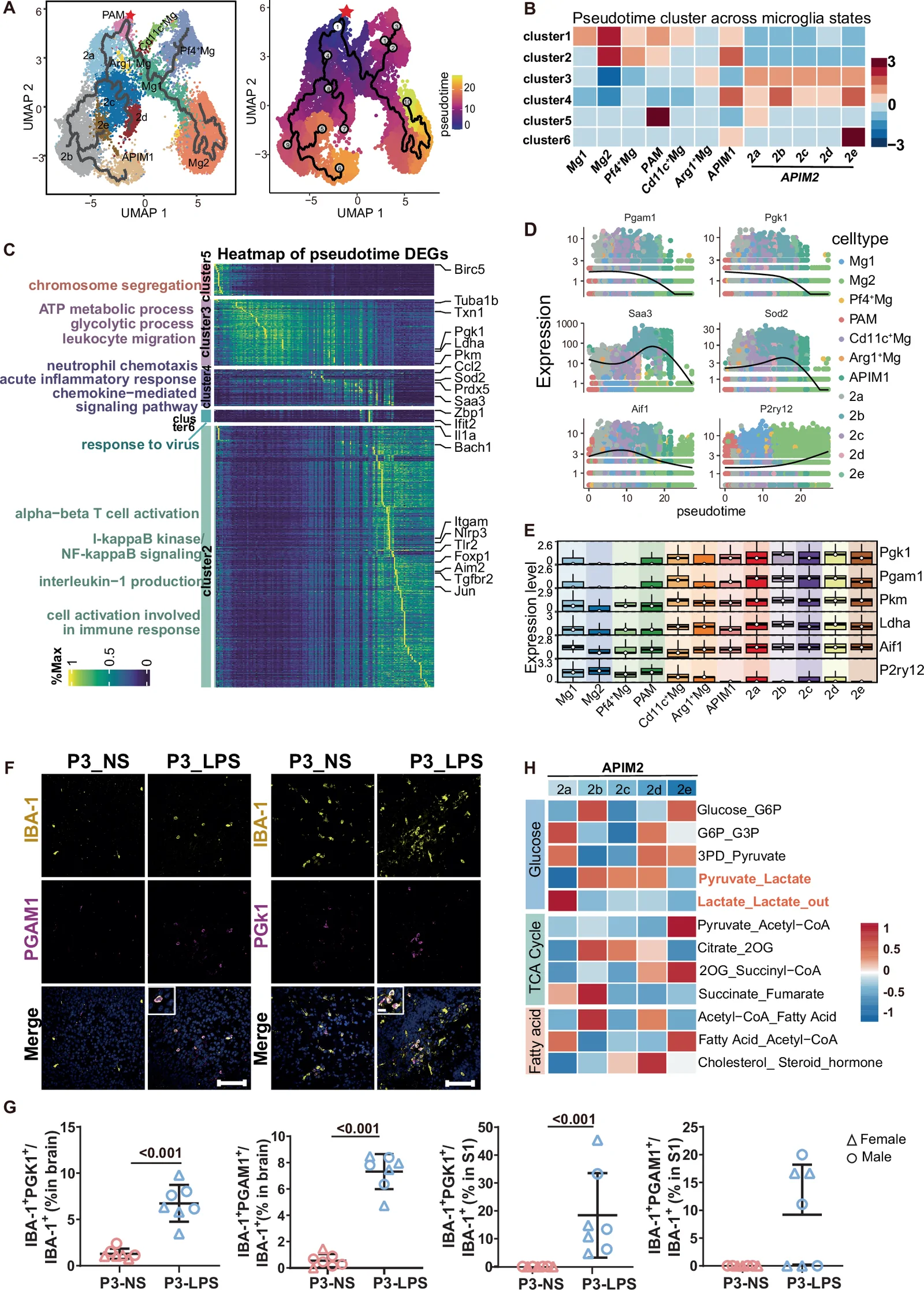
Pseudotime trajectory and metabolic signatures of P3 microglia following neonatal LPS exposure. **A** Pseudotemporal trajectory of P3 microglia. UMAP plots show cells colored by microglial state or pseudotime (purple, early; yellow, late). Black lines indicate the inferred principal graph, and the red star marks the root node within PAM. **B** Relative expression of pseudotime-associated gene modules across P3 microglial states; values were *z*-score scaled per module. **C** Pseudotime-varying genes ordered along the trajectory, with representative enriched GO-BP terms for each module. **D** Expression dynamics of *Pgam1*, *Pgk1*, *Saa3*, *Sod2*, *Aif1* and *P2ry12* along pseudotime. Points represent cells colored by microglial state; curves indicate fitted expression trends. **E** Normalized expression of representative genes across P3 microglial states. Box plots show the median (central line), 25th and 75th percentiles (box limits), and whiskers extending to the most extreme values within 1.5 times the interquartile range. Cell numbers were 1553, 4314, 1726, 307, 314, 144, 957, 3257, 1338, 1852, 542, and 432 for Mg1, Mg2, Pf4⁺Mg, PAM, CD11c⁺Mg, Arg1⁺Mg, APIM1, and the APIM2a–APIM2e subclusters, respectively; *n* denotes cells. Cells were derived from three NS-treated and two LPS-treated male mice. **F** Representative immunofluorescence images of PGAM1 or PGK1 with IBA-1 in NS- and LPS-treated mice at P3. Scale bars, 100 μm for main images and 10 μm for magnified insets. **G** Proportions of IBA-1⁺ cells coexpressing PGK1 or PGAM1 in whole-brain sections and S1. *n*  =  7 mice per group. Data are mean ± SEM. Whole-brain PGK1 and PGAM1 data were analyzed using two-sided Welch’s and unpaired Student’s *t* tests, respectively; S1 data were analyzed using two-sided exact Mann–Whitney *U* tests. Triangles and circles indicate female and male mice, respectively. **H** Average inferred metabolic fluxes in glycolysis, the tricarboxylic acid cycle, and fatty-acid metabolism across APIM2 substates, estimated using scFEA. Color indicates relative flux after min–max normalization within each metabolic module. PAM proliferation-associated microglia, APIM2 acute post-inflammatory microglial state 2, GO-BP Gene Ontology biological process, S1 primary somatosensory cortex, scFEA single-cell flux estimation analysis, UMAP uniform manifold approximation and projection. Source data are provided as a Source Data file.

We then placed this APIM2 continuum within the broader P3 microglial response using Monocle 3 pseudotime analysis, operationally rooted in PAM. APIM2 was positioned along an inferred acute remodeling axis in which pseudotime-associated modules progressed from chromosome segregation, translation, ATP metabolism, and glycolysis toward chemokine response, NF-κB signaling, interleukin-1 production, and interferon/antiviral programs (Fig. 7A–C). Representative gene dynamics supported this graded organization: glycolysis-associated genes, including *Pgam1* and *Pgk1*, were enriched at early pseudotime and declined thereafter, whereas stress- and inflammation-related genes such as *Saa3* and *Sod2* increased along the axis. *Aif1* showed an early increase followed by a decline, while *P2ry12* gradually increased toward later pseudotime (Fig. 7D, E). Consistent with the early glycolytic module, P3 immunostaining showed increased PGAM1 and PGK1 signals in IBA-1^+^cells after LPS exposure. In S1, PGK1^+^IBA-1^+^ cells were significantly increased, whereas PGAM1^+^IBA-1^+^ cells showed an upward trend (Fig. 7F, G). These analyses place APIM2 within a graded acute remodeling axis characterized by early glycolytic activation followed by progressive enrichment of inflammatory and antiviral programs.

We next examined whether this transcriptional continuum was accompanied by metabolic heterogeneity within APIM2. scFEA-based flux inference suggested substate-specific metabolic flux patterns concordant with the APIM2 gradient. APIM2a, positioned near the early glycolytic end of the continuum, showed the strongest inferred lactate-export flux, whereas APIM2e, positioned toward the later inflammatory end, showed higher inferred pyruvate-to-acetyl-CoA flux and downstream TCA-associated modules (Fig. 7H). These inferred flux patterns suggest that APIM2 diversification involves coordinated transcriptional and metabolic remodeling while remaining within a broadly glycolytic acute-response framework.

Finally, we used BAM-focused analyses as a contextual comparison for inflammatory remodeling across brain-resident macrophage compartments. BAMs also underwent acute metabolic-inflammatory remodeling after neonatal LPS exposure, followed by a shift toward more immunoregulatory features by P12 (Figs. S10, S11 and Supplementary Data 9). However, BAMs did not show enrichment of developmental transcriptional signatures associated with *Numb*^+^Mg under physiological conditions. Thus, neonatal inflammation induced metabolic-inflammatory remodeling across brain-resident macrophage compartments, but attenuation of the *Numb*^+^Mg-associated developmental transcriptional features was specific to the parenchymal microglial response rather than the BAM response.

Together, these analyses refine APIM2 as a heterogeneous early remodeling state that maintains glycolytic features while diversifying along inflammatory and antiviral axes. This APIM2 continuum marks the earliest immunometabolic phase of inflammation-disrupted microglial maturation, preceding the later RPIM-dominant landscape and impaired establishment of physiological *Numb*^+^Mg.

## Discussion

Our study identifies *Numb*^+^Mg as a temporally restricted postnatal microglial state associated with brain maturation. This finding indicates that early-life microglial maturation is not simply a linear acquisition of homeostatic identity, but includes stage-specific states aligned with defined phases of brain development. In mice, *Numb*^+^Mg is distinct from the earlier Cd11c⁺Mg state associated with white-matter development and myelination, supporting the concept that successive developmental stages are accompanied by different microglial states^20^. The coupling of neurodevelopment-associated and metabolic features in *Numb*^+^Mg further suggests that developmental microglial states may be metabolically configured to adapt to the changing cellular demands associated with dendritic growth, synaptogenesis, and circuit assembly. This concept is broadly consistent with reported neuron-associated microglial states in the human fetal brain, although those states appear molecularly distinct from *Numb*^+^Mg and are characterized by neuronal transcripts such as *SOX11*, *MEG3*, and *TUBB2A*^11,44^. Thus, *Numb*^+^Mg supports a broader principle that early-life neurodevelopment is accompanied by temporally restricted and context-specific microglial states whose molecular identities may vary across species, developmental stages, and tissue environments.

Beyond its neurodevelopment-associated transcriptional programs, a defining feature of *Numb*^+^Mg is its restricted early postnatal timing. In our dataset, *Numb*^+^Mg emerged during the first two postnatal weeks and was most prominent around P12, whereas in situ RNAscope showed a subsequent decline of *Numb*-expressing microglia by P17. This temporal profile is further supported by published developmental microglial atlases, in which later postnatal and adult microglia are predominantly assigned to homeostatic or maturation-associated states, with no dominant *Numb*^+^Mg-like state reported at P21, P30, or later stages^14,17,19^. These observations suggest that *Numb*^+^Mg is unlikely to represent a long-lasting microglial identity but instead reflects an early postnatal-enriched state within a restricted developmental window. This timing is notable because it overlaps with a period of active synaptogenesis, dendritic maturation, and circuit refinement. Although the PLX5622 depletion strategy was not *Numb*^+^Mg-specific, broad microglial depletion across this window spared gross myelination but altered synaptic protein composition, dendritic architecture, and laminar maturation, supporting the functional importance of microglia during this stage. The preferential structural phenotype in S1 was not accompanied by regional enrichment of Numb-expressing IBA-1^+^microglia, arguing against a simple model in which local accumulation of *Numb*^+^Mg-associated microglia explains the regional vulnerability. Instead, S1 may be particularly sensitive to microglial perturbation because somatosensory circuits undergo rapid postnatal plasticity and maturation during this period, with heightened susceptibility before P14–P15^45–48^. Thus, *Numb*^+^Mg represents a temporally restricted microglial state whose emergence coincides with a sensitive time window for region-specific cortical circuit maturation.

Our findings point to immunometabolic remodeling as an early axis through which neonatal inflammation may perturb microglial developmental timing. The APIM2-dominant response at P3 suggests that the initial microglial response to neonatal LPS is not defined solely by canonical cytokine-driven activation, but also by early metabolic reconfiguration. This interpretation is consistent with evidence that glycolytic metabolism shapes microglial inflammatory activation and suggests that this relationship is also relevant to the neonatal brain, where metabolic rewiring may influence not only acute immune responses but also the subsequent establishment of development-associated and homeostatic microglial states^49,50^. Metabolic–inflammatory remodeling was also evident in BAM-related macrophage compartments, indicating that neonatal inflammation engages multiple brain-resident myeloid compartments. However, the developmental consequence appears most directly linked to parenchymal microglia: *Numb*^+^Mg is normally acquired by parenchymal microglia and is markedly attenuated after neonatal inflammation. Thus, neonatal inflammation may affect brain maturation not simply by inducing inflammatory myeloid states, but by altering the developmental timing and state organization of parenchymal microglia.

Several considerations define the scope of this study. First, our analyses identify *Numb*^+^Mg as a temporally restricted early postnatal microglial state at the population level, but do not resolve the fate of individual *Numb*^+^Mg cells beyond this window. Lineage tracing or temporally controlled fate mapping will be needed to determine whether *Numb*^+^Mg cells transition toward Mg1/homeostatic identities, pass through intermediate states, or are replaced by other microglial states. Second, PLX5622 perturbs microglia during the developmental interval in which *Numb*^+^Mg and Mg1 normally expand, but does not selectively ablate *Numb*^+^Mg. Thus, the resulting phenotypes should be interpreted as consequences of early postnatal microglial depletion rather than evidence of *Numb*^+^Mg-specific causality. Third, the proposed relationship between early immunometabolic remodeling and later attenuation of *Numb*^+^Mg remains based on temporal and transcriptional associations. Causal testing will require microglia-directed manipulation of candidate metabolic regulators, such as Hif1a or Ldha. Finally, only male mice were used for the scRNA-seq experiments, whereas both sexes were included in the downstream validation and functional experiments. The present scRNA-seq dataset was therefore not designed to assess sex-dependent differences in developmental or inflammation-associated microglial states. Larger sex-balanced cohorts will be required to determine whether *Numb*^+^Mg dynamics or inflammatory vulnerability differ between males and females.

In summary, our study identifies *Numb*^+^Mg as a temporally restricted early postnatal microglial state aligned with cortical maturation and shows that neonatal inflammation impairs its establishment. These findings suggest that early-life inflammation disrupts brain development not simply by inducing inflammatory activation, but by altering the timing and identity of microglial states normally engaged during early postnatal maturation. This work provides a cellular and transcriptional framework for understanding how neonatal immune challenge may increase vulnerability to impaired cortical maturation and adverse neurodevelopmental outcomes.

## Methods

### Animals

C57BL/6J mice (*Mus musculus*) were obtained from Vital River (Beijing, China). Adult male and female mice aged 8–10 weeks were used for breeding. Neonatal and juvenile offspring were studied from P2 to P45, depending on the experiment. Mice were housed in the animal facility of the Third Affiliated Hospital of Zhengzhou University at an ambient temperature of 20–22 °C and a relative humidity of 40–60%, under a 12-h light–dark cycle, with ad libitum access to food and water. A total of 302 mice were used in this study.

For scRNA-seq, only male neonatal pups were used to limit biological variability in the discovery dataset and facilitate interpretation of developmental microglial state transitions. For all other experiments, pups of both sexes were included and randomly assigned to experimental groups, with the sex distribution balanced between groups. Sex was recorded for each animal and is indicated by distinct symbols in the relevant figures. Sex-disaggregated data are provided in the Source Data file. Exact animal numbers, ages, and sex distributions for individual experiments are reported in the corresponding Methods sections and figure legends.

All animal procedures were approved by the Ethics Committee of the Third Affiliated Hospital of Zhengzhou University under protocol no. 2024-292-01.

### Drug administration

A neonatal sepsis model was established by subcutaneous injection of lipopolysaccharide (LPS; *Escherichia coli* O111:B4; Sigma, Cat. No. L2630) on P2. Only pups weighing 1.5–2.0 g were included. LPS was administered at 5 mg kg⁻¹ in sterile saline at an injection volume of 10 μl g⁻¹ body weight. Control animals received an equivalent volume of sterile saline.

For microglial depletion, PLX5622 (MCE, Cat. No. HY-114153) was dissolved in DMSO to prepare a 50 mg ml⁻¹ stock solution and subsequently diluted in a vehicle containing 45% PEG300, 5% Tween-80, and 50% sterile saline. PLX5622 was administered at 50 mg kg⁻¹. Beginning on P5, pups received daily intraperitoneal injections of either PLX5622 or vehicle for seven consecutive days. The working solution was freshly prepared before each injection. Animals were assigned to the PLX5622 (Plx) or vehicle (Ve) group.

### RNAscope fluorescent in situ hybridization

RNAscope fluorescent in situ hybridization was performed on 10-μm fresh-frozen brain sections using the RNAscope Multiplex Fluorescent Reagent Kit v2 (ACD Bio-Techne, Cat. No. 323100) according to the manufacturer’s instructions. Sections were hybridized with a probe against *Numb* (Mm-*Numb*-C2, Cat. No. 473401-C2), followed by IBA-1 immunofluorescence staining mouse monoclonal anti-IBA-1 antibody (1:500; Oasis, Cat. No. OB-MMS039-01) to identify microglia.

Images used for quantitative analysis were acquired using an Apotome 3 microscope (Carl Zeiss, Oberkochen, Germany) with a 20× objective under identical acquisition settings across experimental groups. Representative high-resolution images were acquired using a Nikon AX R NSPARC ultra-fast spectral scanning confocal microscope (Nikon Corporation, Japan) with z-stack scanning, followed by maximum-intensity projection. For each animal, four randomly selected fields were acquired from each indicated brain region. Quantification was performed using HALO AI image analysis software. The proportion of *Numb*^+^ IBA-1^+^ microglia was calculated as the number of *Numb*^+^ IBA-1^+^ cells divided by the total number of IBA-1^+^ cells in each field. The four field-level values were averaged to generate one regional value per animal, which was used as the biological replicate for statistical analysis. Animals from four independent litters were included in this experiment; the exact number of animals per group is provided in the corresponding figure legend.

### Behavioral tests

#### O-maze test

At P43, exploratory behavior was assessed using an O-maze apparatus consisting of two open arms and two enclosed arms arranged in a circular track (diameter 60 cm) elevated 40 cm above the ground. The test was conducted under 100 lux lighting in a quiet, dimly lit room between 14:00 and 17:00. Mice were habituated to the test room for 1 h prior to testing. Each mouse was gently placed on the maze and allowed to explore for 5 min. The number of entries, time spent, and distance traveled in each arm were recorded using a video tracking system (Anymaze, USA). Anxiety levels were evaluated by comparing the time spent in open versus enclosed arms. Animals from eight independent litters were included in this experiment; the exact number of animals per group is provided in the corresponding figure legend.

#### Social interaction test

At P45, social behavior was evaluated using a three-chamber apparatus (60 × 40 × 22 cm) under the same environmental conditions. The test followed a two-phase social-exploration protocol. In phase one, the subject mouse was placed in the central chamber with access to all chambers and allowed to explore freely for 10 min. In phase two, a stranger mouse (age- and sex-matched, unfamiliar) was placed in a wire cage in one side chamber, while the opposite chamber contained an empty cage. The partitions were removed, and the subject mouse was allowed to explore for 10 min. The sociability index was calculated as (Social exploration – Empty exploration) / (Social Exploration + Empty exploration). Behavior was recorded and analyzed with the ANY-maze software. Animals from eight independent litters were included in this experiment; the exact number of animals per group is provided in the corresponding figure legend.

### RT–qPCR analysis

Total RNA was extracted using the EasySpin Plus Micro Kit (Aidlab, China). RNA concentration and purity were assessed by measuring the A260/A280 ratio using a UV spectrophotometer. Complementary DNA (cDNA) was synthesized by reverse transcription, and quantitative real-time PCR was performed using a LightCycler 96 system (Roche, USA).

PCR amplification was performed with an initial denaturation step at 95 °C for 3 min, followed by 40 cycles of denaturation at 95 °C for 10 s and annealing and extension at 60 °C for 30 s. Melting-curve analysis was performed from 65 °C, with an increase of 0.5 °C per cycle for 41 cycles. Relative gene-expression levels were calculated using the ${2}^{-\Delta \Delta {C}_{{{{\rm{t}}}}}}$ method, with *Gapdh* as the reference gene. Primer sequences are provided in Supplementary Table 1. Animals from seven independent litters were included in this experiment; the exact number of animals in each group is provided in the corresponding figure legend.

### Immunofluorescence staining

Mice were anesthetized with sodium pentobarbital and transcardially perfused with 4% paraformaldehyde (PFA) in PBS. Brains were either dehydrated through graded ethanol and xylene for paraffin embedding or cryoprotected in 30% sucrose at 4 °C until fully equilibrated. Paraffin-embedded brains were sagittally sectioned at a thickness of 5 μm using a rotary microtome, whereas cryoprotected brains were sectioned at a thickness of 30 μm using a freezing microtome. Antigen retrieval was performed using sodium citrate buffer (pH 6.0) or Tris–EDTA buffer (pH 9.0), depending on the primary antibody. Sections were washed with PBS and blocked with 5% goat serum for 1 h at room temperature. Sections were then incubated with primary antibodies overnight at 4 °C, followed by the appropriate fluorophore-conjugated secondary antibodies for 1 h at 37 °C.

The following primary antibodies were used: rabbit polyclonal anti-MBP (1:500; Abcam, Cat. No. ab40390), guinea pig polyclonal anti-Olig2 (1:500; Oasis, Cat. No. OB-PGP040-01), mouse monoclonal anti-IBA-1 (1:500; Oasis, Cat. No. OB-MMS039-01), rabbit polyclonal anti-IBA-1 (1:200; Wako, Cat. No. 019-19741), rat polyclonal anti-P2RY12 (1:200; Oasis, Cat. No. OB-PRT120), rat polyclonal anti-Cux1 (1:500; Oasis, Cat. No. OB-PRT034), rabbit polyclonal anti-Ctip2 (1:500; Oasis, Cat. No. OB-PRB025-02), guinea pig polyclonal anti-Tbr1 (1:500; Oasis, Cat. No. OB-PGP023-02), rabbit polyclonal anti-PGK1 (1:200; Proteintech, Cat. No. 17811-1-AP), and mouse monoclonal anti-PGAM1, clone 2H2A9 (1:500; Proteintech, Cat. No. 67470-1-Ig).

The following secondary antibodies were used: Alexa Fluor 488-conjugated goat anti-guinea pig IgG (1:1000; Oasis, Cat. No. G-RT488), Alexa Fluor 594-conjugated goat anti-rat IgG (1:1000; Oasis, Cat. No. G-RT594), Alexa Fluor 555-conjugated goat anti-rabbit IgG (H + L) (1:3000; Abcam, Cat. No. ab150078), Alexa Fluor Plus 647-conjugated goat anti-rabbit IgG (H + L) (1:3000; Invitrogen, Cat. No. A37233), and Alexa Fluor 647-conjugated goat anti-mouse IgG (H + L) (1:3000; Invitrogen, Cat. No. A-21235).

Fluorescence images were acquired using a Nikon AX R NSPARC ultra-fast spectral scanning confocal microscope (Nikon Corporation, Japan). Whole-slide images were acquired using a KF-PRO-005 digital slide scanner (KFBIO, China). Animals from six independent litters were included in these experiments; the exact number of animals in each group is provided in the corresponding figure legends.

### Western blotting

Whole brains were collected from individual mice at P21 and homogenized for protein extraction. Each sample represented one mouse. Animals from three independent litters were included in these analyses; the exact number of animals in each group is provided in the corresponding figure legend. Total protein (25 μg per sample) was denatured, separated on 15% SDS–polyacrylamide precast gels (YEASEN, China), and transferred onto 0.45-μm polyvinylidene difluoride membranes (Invitrogen, Thermo Fisher Scientific, USA). Membranes were blocked and incubated with primary antibodies overnight at 4 °C, followed by incubation with the appropriate horseradish peroxidase (HRP)-conjugated secondary antibodies. After washing with TBST, immunoreactive bands were visualized using a ChemiDoc XRS^+^ imaging system (Bio-Rad, USA), and band intensities were quantified using Image Lab v6.0 software (Bio-Rad). GAPDH was detected on the same membrane as the corresponding target protein and used as the loading control. Uncropped and unprocessed scans of all western blots are provided in the Source Data file.

The following primary antibodies were used: rabbit monoclonal anti-VGLUT1, clone EPR22269 (1:1000; Abcam, Cat. No. ab227805), rabbit polyclonal anti-VGAT (1:1000; Proteintech, Cat. No. 14471-1-AP), rabbit polyclonal anti-gephyrin (1:1000; Proteintech, Cat. No. 12681-1-AP), mouse monoclonal anti-PSD-95, clone 6G6-1C9 (1:1000; Abcam, Cat. No. ab2723), and rabbit monoclonal anti-GAPDH, clone D16H11 (1:3000; Cell Signaling Technology, Cat. No. 5174S). The following secondary antibodies were used: HRP-conjugated Affinipure goat anti-mouse IgG (H + L) (1:10,000; Proteintech, Cat. No. SA00001-1) and HRP-conjugated Affinipure goat anti-rabbit IgG (H + L) (1:10,000; Proteintech, Cat. No. SA00001-2.

### Golgi staining

Neuronal morphology in P21 mouse brains was examined using the FD Rapid GolgiStain™ Kit (FD NeuroTechnologies, Cat. No. PK401) according to the manufacturer’s protocol. After fixation and impregnation, brains were sectioned at a thickness of 100 μm using a cryostat. Sections were mounted, stained, dehydrated, cleared in xylene, and coverslipped using neutral resin.

For each animal, a variable number of pyramidal neurons meeting the predefined morphological criteria were reconstructed in each analyzed brain region or cortical layer. Neurons were included when they showed clear and continuous staining from the soma to the distal dendrites and could be distinguished from neighboring neurons. Brightfield images were acquired using an Apotome 3 microscope (Carl Zeiss, Oberkochen, Germany). Three-dimensional neuronal reconstruction and Sholl analysis were performed manually using Neurolucida® 360 software (MBF Bioscience, Williston, USA). Mean dendritic length and the number of dendritic intersections were quantified. For each brain region or cortical layer, measurements from all reconstructed neurons were averaged within each mouse to generate one mouse-level value, which was used as the biological replicate for statistical analysis. The exact numbers of reconstructed neurons for each mouse and region are provided in the Source Data file. Animals from five independent litters were included in this analysis.

### Tissue collection and single-cell suspension preparation

Mice were anesthetized with sodium pentobarbital and transcardially perfused with cold D-PBS. Whole brains were dissected, transferred to RPMI 1640 medium supplemented with 5% FBS (Gibco, Cat. No. C11875500CP), and maintained on ice. Single-cell suspensions were prepared using the Neonatal Brain Dissociation Kit (Miltenyi Biotec, Cat. No. 130-092-628) according to the manufacturer’s protocol. Brain tissues were minced, enzymatically digested with 950 μl Buffer X, 25 μl Enzyme P, 10 μl Buffer Y, and 5 μl Enzyme A, and processed using a gentleMACS™ Octo Dissociator with Heaters with the NTDK program. The resulting cell suspensions were filtered through a 40-μm cell strainer and centrifuged at 500 × *g* for 5 min. Cell pellets were resuspended in 30% Percoll (Sigma, Cat. No. P7828) and centrifuged at 1000 × *g* for 30 min without braking to remove myelin. The resulting cell pellets were collected, washed, and centrifuged again before downstream applications.

### Flow cytometry and cell sorting

Single-cell suspensions were incubated with anti-mouse CD16/CD32 to block Fc receptors and subsequently stained with FVS510 viability dye (BD Biosciences, Cat. No. 564406), APC-conjugated rat monoclonal anti-P2RY12 antibody, clone S16007D (1:200; BioLegend, Cat. No. 848006), and PE–Cy7-conjugated rat monoclonal anti-CD11b antibody, clone M1/70 (1:200; BD Biosciences, Cat. No. 552850). The gating strategy is shown in Supplementary Fig. S1c. Data were acquired using a FACSCanto II flow cytometer (BD Biosciences) and analyzed using FlowJo v10.0 software.

Brain tissues, including the meninges but excluding the cerebellum and olfactory bulbs, were dissociated into single-cell suspensions as described above. CD45⁺ cells were enriched using the EasySep™ Mouse CD45 Positive Selection Kit (STEMCELL Technologies, Cat. No.18945) according to the manufacturer’s instructions. The resulting CD45⁺ cell pellets were stored at −80 °C until RNA extraction.

### Single-cell RNA-seq library construction and sequencing

For single-cell RNA-seq analysis, only male neonatal pups were used to limit biological variability and facilitate the interpretation of developmental microglial state transitions. scRNA-seq libraries were prepared using the SeekOne® Digital Droplet Single Cell 3′ Library Preparation Kit (SeekGene, Cat. No. K00202) according to the manufacturer’s protocol. Briefly, cells were mixed with reverse-transcription reagents and loaded into the sample wells of a SeekOne® S3 microfluidic chip. Barcoded hydrogel beads and partitioning oil were subsequently added to the corresponding wells to generate emulsion droplets. Reverse transcription was performed within the droplets at 42 °C for 90 min, followed by enzyme inactivation at 85 °C for 5 min.

After droplet disruption, barcoded cDNA was purified and amplified by PCR. The resulting cDNA was fragmented, end-repaired, A-tailed, and ligated to sequencing adapters. Indexed PCR was performed to amplify the 3′ ends of polyadenylated transcripts containing cell barcodes and unique molecular identifiers (UMIs). Final libraries were purified using VAHTS DNA Clean Beads (Vazyme, Cat. No. N411-01) and assessed using a Qubit Fluorometer (Thermo Fisher Scientific, Cat. No. Q33226) and a Qsep400 Bio-Fragment Analyzer (BiOptic). Libraries were sequenced on an Illumina NovaSeq 6000 or DNBSEQ-T7 platform using 150-bp paired-end reads.

### Integration and quality control

All analyses were performed in R v4.1.2 unless otherwise specified. Single-cell transcriptomic data were processed using Seurat v4.2.1, and batch effects among samples were corrected using Harmony v0.1.0. Data from individual samples were normalized before being combined for downstream analysis. Low-quality cells were excluded according to the following criteria: fewer than 200 or more than 3000 detected genes (nFeature_RNA), mitochondrial transcript content greater than 5%, or more than 30% of transcripts derived from heat-shock protein (*Hsp*) family genes^16,51,52^. Clusters containing fewer than 200 cells were excluded from downstream analyses.

### Dimensionality reduction and clustering

Gene-expression data were normalized using the NormalizeData function in Seurat and subsequently centered and scaled using ScaleData. Principal-component analysis was performed using RunPCA. The number of principal components retained for downstream analysis was determined using ElbowPlot or JackStrawPlot, with the first 30–40 principal components typically selected.

Unsupervised clustering was performed using the FindNeighbors and FindClusters functions based on a shared nearest-neighbor graph. The clustering resolution was empirically selected to identify biologically meaningful cell states. Cluster marker genes were identified using FindAllMarkers with a two-sided Wilcoxon rank-sum test and Bonferroni adjustment for multiple comparisons. Cell identities were assigned by comparing cluster-enriched genes with established cell-type markers reported in published mouse brain single-cell datasets. UMAP plots, violin plots, dot plots, and heatmaps were generated using Seurat and ComplexHeatmap v2.14.0.

### Cross-dataset comparison with an external developmental microglial dataset

The previously published developmental microglial dataset GSE121654 was used for cross-dataset comparison. Cells from the E14.5, P4/P5, and P30 stages were included. The external dataset and our annotated microglial dataset were processed separately, and quality control of the external dataset was performed according to the criteria described in the original study.

For reference mapping, our annotated P3, P7, and P12 microglial dataset was used as the reference and GSE121654 as the query. Transfer anchors were identified using Seurat v4.2.1, and query cells were projected into the reference low-dimensional space using MapQuery. For co-embedding analysis, reciprocal PCA-based integration was performed in Seurat, followed by dimensionality reduction and UMAP visualization in the integrated space.

### Pathway enrichment analysis

Differentially expressed genes for each cluster were identified using FindAllMarkers with an adjusted *p* value  <  0.01 and log2 fold change > 0. Gene symbols were converted to Entrez identifiers using the bitr function in clusterProfiler v4.2.2. Gene Ontology biological-process and Kyoto Encyclopedia of Genes and Genomes pathway enrichment analyses were performed using enrichGO and enrichKEGG, respectively. Enrichment significance was assessed using a one-sided hypergeometric test with Benjamini–Hochberg correction for multiple comparisons. Results were visualized using dot plots.

### Gene set enrichment analysis (GSEA) and functional scoring

Gene set enrichment analysis was performed using the GSEA function in clusterProfiler on gene lists ranked by log2 fold change. Gene symbols were converted to Entrez identifiers before analysis, and enrichment *p* values were adjusted using the Benjamini–Hochberg method.

Functional signature scores were calculated using UCell v2.1.1. The AddModuleScore_UCell function was applied to curated or literature-derived gene sets. UCell scoring is rank-based and reflects the relative enrichment of each gene signature at the individual-cell leve^53^.

### Trajectory inference and pseudotime analysis

Pseudotime trajectories were reconstructed using Monocle3 v1.0.0^54^. Cells were projected into UMAP space, and a principal graph was learned using the learn_graph function. Root nodes were assigned according to the biologically defined starting state for each trajectory, as indicated in the corresponding figures, and cells were ordered along pseudotime using order_cells.

Genes associated with pseudotime progression were identified using Moran’s *I* statistic. Genes with Moran’s *I*  >  0.1 were considered to show pseudotime-dependent expression patterns. Gene Ontology enrichment analysis was performed for the resulting gene modules, and their expression dynamics were visualized using ComplexHeatmap.

### Protein–protein interaction (PPI) network and functional module analysis

Differentially expressed genes were submitted to the STRING database for protein–protein interaction network construction, using a minimum interaction confidence score of 0.7. Network modules were identified using the clustering tools implemented in STRING. Functional enrichment analyses of individual modules were performed using Gene Ontology and Monarch phenotype annotations to characterize the biological functions and phenotype associations of the corresponding genes.

### Single-cell metabolic flux analysis

Single-cell metabolic flux was estimated using single-cell Flux Estimation Analysis (scFEA) v1.1.2, a neural network-based framework that infers intracellular metabolic fluxes from enzyme-encoding gene expression under flux-balance constraints^43. Gene-expression matrices derived from the Seurat objects were analyzed using the official Python implementation of scFEA. Metabolic networks were represented as functional modules, and relative flux values were inferred for individual cells. Results were visualized in R using pheatmap, ggplot2, and dplyr.

### Statistical analysis

All statistical analyses were performed using IBM SPSS Statistics v21.0 (IBM, USA) and R v4.1.2. Unless otherwise stated, data are presented as mean ± standard error of the mean (SEM), and all statistical tests were two-sided. Normality and homogeneity of variance were assessed using the Shapiro–Wilk and Levene’s tests, respectively.

For comparisons between two independent groups, an unpaired Student’s *t* test was used when the data were normally distributed with equal variances, whereas Welch’s *t*-test was used when variances were unequal. The Mann–Whitney *U* test was used for data that did not satisfy the normality assumption. For comparisons among three or more groups, one-way ANOVA followed by Tukey’s multiple-comparisons test was used when the assumptions of normality and homogeneity of variance were met. Welch’s ANOVA followed by the Games–Howell multiple-comparisons test was used when variances were unequal. For non-normally distributed data, the Kruskal–Wallis *H* test followed by Dunn’s multiple-comparisons test was used.

For experiments involving two factors, two-way ANOVA followed by Bonferroni-adjusted multiple comparisons was used when the parametric assumptions were satisfied. Otherwise, the Scheirer–Ray–Hare test was used, followed by appropriate non-parametric simple-effects analyses. Exact sample sizes, statistical tests, multiple-comparison procedures, and definitions of the experimental unit are provided in the corresponding figure legends. *p*  <  0.05 was considered statistically significant.

### Reporting summary

Further information on research design is available in the Nature Portfolio Reporting Summary linked to this article.

## Supplementary information


Supplementary Information
Description of Additional Supplementary Files
Supplementary Data 1
Supplementary Data 2
Supplementary Data 3
Supplementary Data 4
Supplementary Data 5
Supplementary Data 6
Supplementary Data 7
Supplementary Data 8
Supplementary Data 9
Reporting Summary
Transparent Peer Review file


## Source data


Source data


## Data Availability

The scRNA-seq data generated in this study are available in the NCBI Gene Expression Omnibus under accession code GSE337230 and in the NCBI Sequence Read Archive under BioProject accession PRJNA1329933. The previously published developmental microglial dataset used for cross-dataset comparison is available in the NCBI Gene Expression Omnibus under accession code GSE121654. Source data underlying the figures and tables, including uncropped scans of all western blots presented in the main figures, are provided with this paper in the Source Data file. Source data are provided with this paper.

## Code availability

The custom code used for the scRNA-seq and spatial transcriptomic analyses in this study is available on GitHub at https://github.com/fafax093-design/scRNA-neonatal-microglia-analyses-2025 and has been archived on Zenodo at https://doi.org/10.5281/zenodo.20795495^55^.
