# Supplementary material for: Neonatal inflammation disrupts a temporally restricted postnatal Numb-enriched microglial state in mice
Source: Nat Commun. 2026 Aug 4;17:9385. doi: 10.1038/s41467-026-76341-6 (PMC13534487; doi:10.1038/s41467-026-76341-6)
Supplement: Supplementary file 1 [file 41467_2026_76341_MOESM12_ESM.pdf]

nature portfolio

Corresponding author(s): Changlian Zhu

Last updated by author(s): 2026.06.26

Reporting Summary

Nature Portfolio wishes to improve the reproducibility of the work that we publish. This form provides structure for consistency and transparency in reporting. For further information on Nature Portfolio policies, see our Editorial Policies and the Editorial Policy Checklist.

Statistics

For all statistical analyses, confirm that the following items are present in the figure legend, table legend, main text, or Methods section.

n/a	Confirmed	
☐	☒	The exact sample size (*n*) for each experimental group/condition, given as a discrete number and unit of measurement
☐	☒	A statement on whether measurements were taken from distinct samples or whether the same sample was measured repeatedly
☐	☒	The statistical test(s) used AND whether they are one- or two-sided<br>*Only common tests should be described solely by name; describe more complex techniques in the Methods section.*
☒	☐	A description of all covariates tested
☐	☒	A description of any assumptions or corrections, such as tests of normality and adjustment for multiple comparisons
☐	☒	A full description of the statistical parameters including central tendency (e.g. means) or other basic estimates (e.g. regression coefficient) AND variation (e.g. standard deviation) or associated estimates of uncertainty (e.g. confidence intervals)
☐	☒	For null hypothesis testing, the test statistic (e.g. *F*, *t*, *r*) with confidence intervals, effect sizes, degrees of freedom and *P* value noted<br>*Give P values as exact values whenever suitable.*
☒	☐	For Bayesian analysis, information on the choice of priors and Markov chain Monte Carlo settings
☒	☐	For hierarchical and complex designs, identification of the appropriate level for tests and full reporting of outcomes
☒	☐	Estimates of effect sizes (e.g. Cohen's *d*, Pearson's *r*), indicating how they were calculated

Our web collection on statistics for biologists contains articles on many of the points above.

Software and code

Policy information about availability of computer code

Data collection	Single-cell RNA-sequencing libraries were generated using the SeekOne® Digital Droplet Single Cell 3′ Library Preparation Kit and sequenced on an Illumina NovaSeq 6000 or DNBSEQ-T7 platform with paired-end 150-bp reads. Flow cytometry data were acquired using a BD FACSCanto II flow cytometer. Fluorescence and brightfield images were acquired using a Zeiss Apotome 3 microscope, a Nikon AX R NSPARC confocal microscope or a KFBIO KF-PRO-005 digital slide scanner. RT–qPCR data were acquired using a Roche LightCycler 96 real-time PCR system. Western blot images were acquired using a Bio-Rad ChemiDoc XRS+ imaging system, and behavioral data were recorded using ANY-maze v7.4 software.
Data analysis	Single-cell RNA-sequencing data were analyzed in R v4.1.2 using Seurat v4.2.1, Harmony v0.1.0, Monocle3 v1.0.0, clusterProfiler v4.2.2, ComplexHeatmap v2.14.0, UCell v2.1.1 and scFEA v1.1.2. Data processing and visualization were additionally performed using dplyr v1.1.4, ggplot2 v3.5.2 and pheatmap v1.0.12. Flow cytometry data were analyzed using FlowJo v10.0. Fluorescence images were quantified using HALO AI v3.6. Neuronal reconstructions and Sholl analyses were performed using Neurolucida 360 v2020. Western blot densitometry was performed using Image Lab v6.0, and behavioral data were analyzed using ANY-maze v7.4. Statistical analyses were conducted using IBM SPSS Statistics v21.0 and R v4.1.2. Custom analysis code used in this study is available on GitHub at https://github.com/fafax093-design/scRNA-neonatal-microglia-analyses-2025 and has been archived on Zenodo at https://doi.org/10.5281/zenodo.20795495.

For manuscripts utilizing custom algorithms or software that are central to the research but not yet described in published literature, software must be made available to editors and reviewers. We strongly encourage code deposition in a community repository (e.g. GitHub). See the Nature Portfolio guidelines for submitting code & software for further information.

Data

Policy information about availability of data

All manuscripts must include a data availability statement. This statement should provide the following information, where applicable:

- Accession codes, unique identifiers, or web links for publicly available datasets
- A description of any restrictions on data availability
- For clinical datasets or third party data, please ensure that the statement adheres to our policy

The scRNA-seq data generated in this study are available in the NCBI Gene Expression Omnibus under accession code GSE337230 [https://www.ncbi.nlm.nih.gov/geo/query/acc.cgi?acc=GSE337230]. The associated raw sequencing data are available in the NCBI Sequence Read Archive through BioProject accession code PRJNA1329933 [https://www.ncbi.nlm.nih.gov/bioproject/PRJNA1329933]. The previously published scRNA-seq dataset used in this study is available in the Gene Expression Omnibus under accession code GSE121654 [https://www.ncbi.nlm.nih.gov/geo/query/acc.cgi?acc=GSE121654]. Source data are provided with this paper.

Research involving human participants, their data, or biological material

Policy information about studies with human participants or human data. See also policy information about sex, gender (identity/presentation), and sexual orientation and race, ethnicity and racism.

Reporting on sex and gender	NA
Reporting on race, ethnicity, or other socially relevant groupings	NA
Population characteristics	NA
Recruitment	NA
Ethics oversight	NA

Note that full information on the approval of the study protocol must also be provided in the manuscript.

Field-specific reporting

Please select the one below that is the best fit for your research. If you are not sure, read the appropriate sections before making your selection.

- [x] Life sciences
- [ ] Behavioural & social sciences
- [ ] Ecological, evolutionary & environmental sciences

For a reference copy of the document with all sections, see nature.com/documents/nr-reporting-summary-flat.pdf

Life sciences study design

All studies must disclose on these points even when the disclosure is negative.

Sample size	No statistical methods were used to predetermine sample sizes. Sample sizes were based on prior experiments conducted in our laboratory and were similar to those reported in previous publications using comparable neonatal inflammation models and experimental approaches (Zhu et al., 2025, Acta Neuropathol. Commun.; Favuzzi et al., 2021, Cell; Stogsdill et al., 2022, Nature; Tansley et al., 2022, Nat. Commun.). Exact sample sizes and the definitions of n are provided in the corresponding figure legends.
Data exclusions	Animals with a body weight outside the predefined eligibility range of 1.5–2.0 g at the time of LPS administration were not enrolled in the experiment. No other animals were excluded from the analyses.
Replication	All experimental findings in this research were replicated across multiple biological samples. For single-cell RNA-sequencing analyses, the numbers of biologically independent samples and cells are reported separately. The number of replications for each experiment is detailed in the figure legends.
Randomization	Animals were randomly assigned to experimental groups before treatment. For experiments including both sexes, the sex distribution was balanced between groups.
Blinding	Experiments were performed using the numbers of biologically independent mice or samples specified in the corresponding figure legends. Measurements from multiple cells, sections, fields or neurons within the same mouse were treated as subsamples and were averaged or analysed at the mouse/sample level as appropriate. For representative images, the number of independent animals showing similar results is stated in the relevant figure legend. The scRNA-seq analysis included 13 biologically independent samples.

Reporting for specific materials, systems and methods

We require information from authors about some types of materials, experimental systems and methods used in many studies. Here, indicate whether each material, system or method listed is relevant to your study. If you are not sure if a list item applies to your research, read the appropriate section before selecting a response.

Materials & experimental systems

n/a	Involved in the study
	[x] Antibodies
[x]	Eukaryotic cell lines
[x]	Palaeontology and archaeology
	[x] Animals and other organisms
[x]	Clinical data
[x]	Dual use research of concern
[x]	Plants

Methods

n/a	Involved in the study
[x]	ChIP-seq
	[x] Flow cytometry
[x]	MRI-based neuroimaging

Antibodies

Antibodies used

Antibodies used for immunofluorescent included:
Rabbit polyclonal anti-MBP, Abcam, Cat# ab40390, dilution1:500;
Guinea pig polyclonal anti-Olig2, Oasis, Cat# OB-PGP040-01, dilution1:500;
Mouse monoclonal anti-IBA-1, Oasis, Cat# OB-MMS039-01, dilution1:500;
Rabbit polyclonal anti-IBA-1, Wako, Cat# 019-19741, dilution1:200;
Rat polyclonal anti-P2RY12, Oasis, Cat# OB-PRT120, dilution1:200;
Rat polyclonal anti-Cux1, Oasis, Cat# OB-PRT034,dilution1:500;
Rabbit polyclonal anti-Ctip2, Oasis, Cat# OB-PRB025-02,dilution1:500;
Guinea pig polyclonal anti-Tbr1, Oasis, Cat# OB-PGP023-02,dilution1:500;
Rabbit polyclonal anti-PGK1, Proteintech, Cat# 17811-1-AP,dilution1:200;
Mouse monoclonal [2H2A9] anti-PGAM1, Proteintech, Cat# 67470-1-Ig,dilution1:500;
Goat anti-Guinea pig IgG, Alexa Fluor® 488, Oasis, Cat# G-RT488, dilution1:1000;
Goat anti-Rat IgG, Alexa Fluor® 594, Oasis, Cat# G-RT594, dilution1:1000;
Goat anti-Rabbit IgG H&L, Alexa Fluor® 555, Abcam, Cat# ab150078, dilution1:3000;
Goat anti-Rabbit IgG (H+L), Alexa Fluor™ Plus 647, Invitrogen, Cat# A37233, dilution1:3000;
Goat anti-Mouse IgG (H+L), Alexa Fluor™ 647, Invitrogen, Cat# A-21235, dilution1:3000;
Antibodies used for western blot:
Rabbit monoclonal [EPR22269] anti-VGLUT1, Abcam, Cat# ab227805, dilution 1:1000;
Rabbit polyclonal anti-VGAT, Proteintech, Cat# 14471-1-AP, dilution 1:1000;
Rabbit polyclonal anti-Gephyrin, Proteintech, Cat# 12681-1-AP, dilution 1:1000;
Mouse monoclonal [6G6-1C9] anti-PSD-95, Abcam, Cat# ab2723, dilution 1:1000;
Rabbit monoclonal [D16H11]anti-GAPDH, Cell Signaling Technology, Cat# 5174S, dilution 1:3000
HRP-conjugated Affinipure Goat anti-Mouse IgG (H+L), 1:10000, Proteintech, Cat# SA00001-1
HRP-conjugated Affinipure Goat anti-Rabbit IgG (H+L), 1:10000, Proteintech, Cat# SA00001-2
Antibodies used for flow cytometry:
P2RY12 APC (Rat monoclonal clone S16007D, BioLegend, Cat# 848006, dilution 1:200)
CD11b PE-Cy7 (Rat monoclonal clone M1/70 , BD, Cat# 552850, dilution 1:200)

Validation

No new antibodies were generated as part of this study. Commercial primary antibodies were selected on the basis of manufacturer validation for the indicated species and application. The relevant manufacturer validation resources are listed below.
• Rabbit polyclonal anti-MBP, Abcam, Cat# ab40390, dilution1:500, suitable for IF/IHC. https://www.abcam.cn/products/primary-antibodies/myelin-basic-protein-antibody-ab40390.html;
• Guinea pig polyclonal anti-Olig2, Oasis, Cat# OB-PGP040-01, dilution1:500, suitable for IF/IHC. https://www.oasisbiofarm.net/#/productdetails?proId=c2a0bff9db8745dca0f0cca8a35e9887&exp2=%E6%8A%97%E4%BD%93;
• Mouse monoclonal anti-IBA-1, Oasis, Cat# OB-MMS039-01, dilution1:500, suitable for IF/IHC. https://www.oasisbiofarm.net/#/productdetails?proId=85542fb5f9da46d8bfc2824d7b737ca5&exp2=%E6%8A%97%E4%BD%93;
• Rabbit polyclonal anti-IBA-1, Wako, Cat# 019-19741, dilution1:200, suitable for IHC. https://labchem-wako.fujifilm.com/asia/product/detail/W01W0101-1974.html;
• Rat polyclonal anti-P2RY12, Oasis, Cat# OB-PRT120, dilution1:200, suitable for IF/IHC. https://www.oasisbiofarm.net/#/productdetails?proId=180202c4a0e74718a4c9e74812675529&exp2=%E6%8A%97%E4%BD%93;
• Rat polyclonal anti-Cux1, Oasis, Cat# OB-PRT034,dilution1:500, suitable for IF/IHC. https://www.oasisbiofarm.net/#/productdetails?proId=67f31cd0c1a34ee2b7a8e927148f3b53&exp2=%E6%8A%97%E4%BD%93;
• Rabbit polyclonal anti-Ctip2, Oasis, Cat# OB-PRB025-02,dilution1:500, suitable for IF/IHC. https://www.oasisbiofarm.net/#/productdetails?proId=f77eb8ea228e48fb9e69d4d16403c926&exp2=%E6%8A%97%E4%BD%93;
• Guinea pig polyclonal anti-Tbr1, Oasis, Cat# OB-PGP023-02,dilution1:500, suitable for IF/IHC.

https://www.oasisbiofarm.net/#/productdetails?prold=d06b7a3d403142aa826868bf97cd9962&exp2=%E6%8A%97%E4%BD%93;
• Rabbit polyclonal anti-PGK1, Proteintech, Cat# 17811-1-AP,dilution1:200, suitable for IF/IHC. https://ptgcn.com/products/PGK1-Antibody-17811-1-AP.htm;
• Mouse monoclonal [2H2A9] anti-PGAM1, Proteintech, Cat# 67470-1-Ig,dilution1:500, suitable for IF/IHC. https://ptgcn.com/products/PGAM1-Antibody-67470-1-Ig.htm;
• Rabbit monoclonal [EPR22269] anti-VGLUT1, Abcam, Cat# ab227805, dilution 1:1000, suitable for WB. https://www.abcam.cn/products/primary-antibodies/vglut1-antibody-epr22269-ab227805.html;
• Rabbit polyclonal anti-VGAT, Proteintech, Cat# 14471-1-AP, dilution 1:1000, suitable for WB. https://ptgcn.com/products/SLC32A1-Antibody-14471-1-AP.htm;
• Rabbit polyclonal anti-Gephyrin, Proteintech, Cat# 12681-1-AP, dilution 1:1000, suitable for WB. https://ptgcn.com/products/GPHN-Antibody-12681-1-AP.htm;
• Mouse monoclonal [6G6-1C9] anti-PSD-95, Abcam, Cat# ab2723, dilution 1:1000, suitable for WB. https://www.abcam.cn/products/primary-antibodies/psd95-antibody-6g6-1c9-synaptic-markerab2723.html;
• Rabbit monoclonal [D16H11] anti-GAPDH, Cell Signaling Technology, Cat# 5174S, dilution 1:3000, suitable for WB. https://www.cellsignal.cn/products/primary-antibodies/gapdh-d16h11-xp-rabbitmab/5174;
• P2RY12 APC (Rat monoclonal clone S16007D, BioLegend, Cat# 848006, dilution 1:200) , suitable for FC. https://www.biolegend.com/en-us/products/apc-anti-mouse-p2ry12-antibody-15402;
• CD11b PE-Cy7 (Rat monoclonal clone M1/70 , BD, Cat# 552850, dilution 1:200), suitable for FC.https://www.bdbiosciences.com/en-us/products/reagents/flow-cytometry-reagents/researchreagents/single-color-antibodies-ruo/pe-cy-7-rat-anti-cd11b.552850?tab=product_details.

Animals and other research organisms

Policy information about studies involving animals; ARRIVE guidelines recommended for reporting animal research, and Sex and Gender in Research

Laboratory animals

C57BL/6J mice (Mus musculus) were obtained from Beijing Vital River Laboratory Animal Technology Co., Ltd. (Beijing, China). A total of 302 mice were used in this study. Adult male and female mice aged 8–10 weeks were used for breeding, and experimental offspring were studied from postnatal day 2 to postnatal day 45, depending on the experiment. Mice were housed in an animal facility at the Third Affiliated Hospital of Zhengzhou University at an ambient temperature of 20–22°C and a relative humidity of 40–60%, under a 12-h light–dark cycle, with free access to food and water.

Wild animals

This study did not involve wild animals.

Reporting on sex

Only male neonatal mice were used for single-cell RNA-sequencing to reduce biological variability in the discovery dataset. Both male and female mice were included in all other experiments, with sex distribution balanced across experimental groups where feasible. The sex of each animal is provided in the Source Data and indicated in the relevant figures where applicable. Data from both sexes were pooled for the primary analyses because the study was not designed or powered to assess sex-specific effects.

Field-collected samples

This study did not involve field-collected samples.

Ethics oversight

All animal procedures were approved by the Ethics Committee of the Third Affiliated Hospital of Zhengzhou University (approval no. 2024-292-01) and were conducted in accordance with institutional guidelines for the care and use of laboratory animals.

Note that full information on the approval of the study protocol must also be provided in the manuscript.

Plants

Seed stocks

NA

Novel plant genotypes

NA

Authentication

NA

Flow Cytometry

Plots

Confirm that:

- [x] The axis labels state the marker and fluorochrome used (e.g. CD4-FITC).
- [x] The axis scales are clearly visible. Include numbers along axes only for bottom left plot of group (a 'group' is an analysis of identical markers).
- [x] All plots are contour plots with outliers or pseudocolor plots.
- [ ] A numerical value for number of cells or percentage (with statistics) is provided.

Methodology

Sample preparation	Whole brains from neonatal mice were enzymatically dissociated using the Neonatal Brain Dissociation Kit. Cell suspensions were filtered through a 40-µm cell strainer and subjected to 30% Percoll centrifugation to remove myelin. CD45+ cells were subsequently enriched using the EasySep Mouse CD45 Positive Selection Kit. Cells were stained with a fixable viability dye and antibodies against CD11b and P2RY12 to assess the enrichment and purity of the isolated microglial population.
Instrument	Flow cytometry data were acquired using a BD FACSCanto II flow cytometer (BD Biosciences).
Software	Flow cytometry data were acquired using a BD FACSCanto II flow cytometer with BD FACSDiva v8.0.1.1 software and analyzed using FlowJo v10.0.
Cell population abundance	Flow cytometry was used only to assess the enrichment and purity of the CD45+ fraction after magnetic selection. Post-selection purity was determined as the percentage of live singlet cells expressing both CD11b and P2RY12. A representative post-selection sample contained 98.9% CD11b+P2RY12+ cells.
Gating strategy	Cells were sequentially gated based on FSC/SSC characteristics, singlets, live cells, CD11b-positive cells and P2RY12-positive cells. Positive and negative gates were defined using unstained controls, single-stained compensation controls and the clear separation between marker-positive and marker-negative populations.

- [x] Tick this box to confirm that a figure exemplifying the gating strategy is provided in the Supplementary Information.
