# Supplementary material for: Neonatal inflammation disrupts a temporally restricted postnatal Numb-enriched microglial state in mice
Source: Nat Commun. 2026 Aug 4;17:9385. doi: 10.1038/s41467-026-76341-6 (PMC13534487; doi:10.1038/s41467-026-76341-6)
Supplement: Supplementary file 2 [file 41467_2026_76341_MOESM13_ESM.pdf]

nature portfolio

Peer Review File

Neonatal inflammation disrupts a temporally restricted postnatal Numb-enriched microglial state in mice

Corresponding Author: Dr Changlian Zhu

This file contains all reviewer reports in order by version, followed by all author rebuttals in order by version.

Version 0:

Reviewer comments:

Reviewer #1

(Remarks to the Author)
This study by Zhu et al. investigated microglial state and function in early postnatal period of mouse brain development. The author performed single cell transcriptomic profiling of P3, P7, and P12 brain parenchymal microglia, with and without immune perturbation with single dose LPS injection at P2. They uncovered a previously uncharacterized microglial state that they named neurodevelopment-associated microglial state (NMD). Intriguingly, this cluster of microglia increased dramatically during 2nd postnatal week, from 4% at P3 to 11.8% at P7 to 46% at P12. A single dose of LPS injection on P2 alters such developmental trajectory and renders inflammatory microglia transition. To study the function of NMD microglial state, the authors depleted microglia by CSF1R inhibitor PLX5622 injection from P5 to P11. While gross myelination remained unchanged, microglial depletion from P5-11 resulted in increased Gephyrin and PSD-95, reduced dendritic complexity and shorter dendritic length. At functional level, PLX-treated mice demonstrated reduced locomotion and impaired sociability. Microglia show high heterogeneity during brain development and in various diseases, and this study provides an additional layer of understanding in this regard.

Unfortunately, the data presented does not support their overall conclusion, which is “a transient neurodevelopmental microglial state defines a critical period of cortical maturation”. First, their end point was P12. It is not possible to conclude NMD state is transient without including more timepoints beyond P12. Specifically, one doesn’t know how the NDM state evolves beyond P12. More time points beyond P12 are needed to fully support their conclusion. Second, PLX depletes all microglia. NMD cluster only represents 11.8% at P7 and 46% at P12. It is inappropriate to conclude circuit maturation impairment is due to NMD state alone.

Other major concerns include:

1. The behavior analysis showed impaired locomotor activity in PLX group (Fig. 4I), which makes it hard to tease out the effect of reduced locomotor activity in anxiety/social behaviors (similar issue for the LPS group compared to control, especially with myelination phenotype).
2. To elucidate whether second postnatal week involves region-specific changes in microglial density and maturation, the authors performed IHC of IBA1 and PRRY12 across cortex, hippocampus, corpus callosum, and striatum. The whole paper was about NDM expansion and function, it would be desirable to include some markers that defines NDM cluster instead of pan-microglia (IBA1) and homeostatic microglial (P2RY12) markers.
3. There are several published microglia datasets (Hammond, Timothy R., et al. Immunity 50.1 (2019): 253-271; Li, Qingyun, et al. Neuron 101.2 (2019): 207-223; Matcovitch-Natan et al, Science 2016) that include similar (Matcovitch-Natan et al. P3, P6, and P9; Hammond et al. Immunity iP4, and Li, Qingyun, et al. iP7) and additional timepoints (P20, P30). The authors should integrate different datasets to support their conclusion.
4. Fig 4C appears to show more dendritic spines in PLX treated group. This data should be quantified and discussed in the result section.
5. The method section indicated both male and female pups were included. However, no data was shown on both sexes in Fig. 3-5. Literature has demonstrated sex dimorphism in microglial function, especially in setting of immune perturbation. It is important to show the data in the result sections.
6. Lines 326-329: “Following LPS exposure, these developmental and homeostatic states contracted sharply (NDM ~3%, Mg1 ~2%), whereas the recovery-phase inflammatory state RPIM expanded (~49%), indicating a shift from developmental/homeostatic to inflammatory programs.” Is the actual cell number of NDM also different? A decrease in the

NDM percentage may be due to excessive proliferation of PRIM.

Reviewer #2

(Remarks to the Author)
Zhu et al., provide an interesting manuscript that does extensive profiling of microglial states in the developing brain, highlighting the second postnatal week in mice as an underappreciated and thus understudied developmental window. I agree with this sentiment and the authors should be lauded for their detailed analysis of this large sequencing dataset and for integrating many aspects of microglial biology (metabolism, development, inflammation) in their studies. The microglia populations that they identify are indeed novel and will likely provide valuable context to the field. These details aside, the article suffers significantly from a lack of strong focus, disorganized/hard to interpret figures, and little/no concrete identification and tracking of the microglia “subsets” they identified outside of sequencing. If not rectified, these issues will significantly detract from the potential influence their findings might have on the field.

Major Comments:
1. The bioinformatics, while impressive, is extremely overwhelming visually and hard to follow. While it is appreciated that many of the same visualizations and analyses are performed from figure to figure (for consistency), some analyses are unnecessary to the main points of the paper, and the dense organization of the figures makes it difficult and unpleasant to read and properly interpret. Below in minor comments are my suggested changes.
2. Moreover, the current layout of the manuscript emphasizes the importance of NDMs in the first half, then APIMs in the second half, and never produces any strong evidence that these cell subtypes are in any way linked to one another.
3. The identification of “NDM” and “APIM1/2” microglia subsets would be strengthened by additional molecular analyses other than sequencing. Is there some way in saline/control mice to visualize these two groups of microglia at P12? (i.e. some marker, either an antibody or FISH probe, that is + for NDMs, - for Mg1s) so you could histologically determine their specific contributions to neurodevelopment and potentially begin to assign some functional significance to these subsets.
4. The authors repeatedly refer to NDMs as a “a transient, second week-dominant neurodevelopment-associated microglial state”, but they never look for these cells past P12. How long does this microglia population last? Do they die off, or develop into MG1 “homeostatic” mature microglia after a set amount of time? You cannot state that they are transient without showing that they disappear shortly after appearing.
5. The manuscript does not do a good job of properly justifying analyses in certain brain regions.
o Are these novel subtypes identified present in equal proportions across cortical, subcortical, and hippocampal regions?
6. Several questions related to experimental design need to be more explicitly addressed, including: How many mice per group? How many litters per group? Male/Female distribution of each group?

Minor Comments:
- Some mention of the LPS condition should be made in figure 1a schematic, so that it is clear to the reader that all data was collected together (important for future comparative analyses).
- The UMAP in 1C should be labelled as “Saline” treated
- Fig 1H volcano plot PEM vs MG1 was not mentioned in the text. Is this visualization necessary to the ultimate conclusions of the paper? If not, it should be removed
- The schematic in 1L makes it look like NDMs are the most mature microglia – is this really the case the authors are trying to make? If so, are they really transient?
- Fig 2C - a title like “pseudo time clusters” would be informative and make the figure more accessible for readers not familiar with monocle
- Fig 2D - letter in the panel is far from the raster plot
- Labels providing context to each of the subplots in 2E would also be helpful (i.e. group them as you describe in the text, “Cell cycle markers” “Neurodevelopment Factors”.
o Apoe and SPP1 are not even mentioned in the text – what is their significance to warrant being included in this set of plots?
- Max intensity projection images of the histology in 2I would be appreciated
- Additionally, a quantification of the % microglia positive for these markers would help with major concern 3 above
- Details about the western blot synapse data should be made more clear in the text and main figure. In fact, I can find no details about how the tissues were collected for this experiment. Was this whole brain tissue? How can the authors address the fact that these synaptic proteins are expressed at different levels across different brain regions?
- The justification in the text for focusing on somatosensory in Figure 4B,C is not accompanied by any data and the representative images are not convincing
- Open area time feels more appropriate than open distance for EZM metric. Also the figure would be easier to read if the EZM graphs accompanied the EZM heatmaps
- Micorglia in UMAP 5E
- Need to make it more clear in Fig 5E that this includes the initial saline data from Figure 1 AND the “new” LPS data
o The BAM data (Fig 5E-F) could be moved to supplement, as it distracts from the focus of the manuscript
- Are APIMs invading macs that become microglia?
- Need to clarify in the figure that the UMAP in 5G is just the LPS mice
- Histology in 5K is nice but it is not entirely related to what the rest of this figure is trying to show. This figure just feels unnecessary
- What is meant by “RPIM underwent striking reprogramming” (Line 333)? Compared to the RPIM clusters in figure 5? How

can that comparison be made?
- Histology corroborating the findings in Figure 6I would be nice
- Need indication that this is now P3 cells only for the UMAP clustering in Fig7A
- Box/highlight to bring APIM1 and APIM2 to attention in 7G. That heatmap is terribly large and mostly irrelevant other than those two groups
- 7H-K belongs in the supplement
- 8D is not showing what is stated in the text
- 8F needs to be labelled as APIM2 cells
- Why hippocampus in 8G-H and not somatosensory cortex?

Reviewer #3

(Remarks to the Author)
In this manuscript by Zhu, Xu et al., the authors aim to unravel the heterogeneity and functional relevance of microglia, the brain resident macrophages, during postnatal development and in the context of neonatal inflammation. They present a comprehensive single-cell atlas of postnatal microglial states in mice at P3, P7, and P12. Using scRNA-seq, pseudotime reconstruction, and functional perturbations (including PLX5622-mediated microglial depletion and neonatal LPS exposure), they identify a novel microglial state that peaks during the second postnatal week, termed NDM. This population is proposed to support cortical dendritic maturation and social behavior. The study further shows that neonatal LPS exposure leads to a reorganization of microglial states, marked by the expansion of metabolically primed inflammatory microglia (APIM2) and incomplete recovery of homeostatic or neurosupportive programs.

Overall, this work identifies an interesting and potentially important transient developmental microglial state with clear relevance for understanding postnatal brain maturation. The authors thus address a major question and provide promising insights into microglial heterogeneity during development. However, several aspects of the study are incomplete, and the different sections are not well integrated. The NDM state is described almost exclusively at the transcriptomic level, with limited in situ validation or spatial characterization. The two main parts of the manuscript (the description of NDM and the analysis of neonatal inflammation) are not well connected making it difficult to assess functional connections between NDM identity, cortical circuit maturation, and the effects of neonatal inflammation.

NDM state characterization

The identification of a new microglial state (NDM) enriched during the second postnatal week is an important finding. However, the spatial and temporal characterization of this state remains incomplete. It is unclear whether NDM microglia are restricted to the somatosensory cortex or broadly distributed across brain regions. The authors should provide both qualitative data from immunolabeling as well as quantification showing the proportion of microglia expressing NDM markers (QK, NAV2, HK2) in relevant regions at different time points.

The authors state that NDM is a transient state, but no data are provided to demonstrate its disappearance at later time points. Including later developmental stages would strengthen this point. Additionally, the acronym NDM may be confusing since it refers to a late developmental window, a clearer designation could help avoid ambiguity in the field (especially with the designation of PEM).

While five co-expression modules are identified, the manuscript does not discuss the regulatory mechanisms or signaling pathways that might drive the Mg1 vs. NDM bifurcation. Highlighting candidate transcription factors or upstream regulators would provide mechanistic insight.
Finally, the authors should show in the scRNA-seq data whether NDM-associated genes are microglia and not shared with border-associated macrophages (BAMs).

Microglia depletion experiments

In the PLX5622 depletion experiments, the kinetics of microglial depletion and repopulation are not shown, yet the authors describe outcomes at P21 and P45, when microglia are expected to have repopulated. Still, it remains unknown when NDM cells disappear.

While we acknowledge that specific ablation of NDM or Mg1 microglia is technically challenging, the authors should describe NDM and Mg1 states before and after PLX treatment. Deciphering whether NDM state is particularly enriched in S1 versus other cortical regions could help to support a causal link between NDM and the dendritic or behavioral outcomes. Including quantitative data from other regions would be important to strengthen the claim that the observed effects are specific of S1.

It would also be important to quantify the repopulation dynamics. It remains unclear whether the reported effects in S1 result solely from the transient absence of microglia/macrophages or from altered properties of repopulated cells that could fail to re-establish homeostatic microglia or NDM-like state if the NDM state would still be present during/after repopulation.

Furthermore, as PLX5622 target CSF1R-expressing macrophages, the authors should temper their conclusions regarding microglia specific roles.

Neonatal inflammation

The LPS-induced neonatal inflammation provide an important link between early life immune challenges and macrophages developmental trajectories, which is highly relevant regarding neurodevelopmental disorders. However, the connection between the first part of the paper (NDM characterization) and the second part (LPS effects) could be strengthen. It would be helpful to analyze whether LPS-induced suppression of NDM microglia leads to similar dendritic defects in S1, as observed following PLX treatment.

The manuscript refers to "persistent" effects of neonatal inflammation, but the latest analyzed time point is P12. To support persistence, later developmental stages (for instance P21 or P45) should be included to assess long-term transcriptional or morphological changes.

To support the proposed model that early metabolic reprogramming (P3) prevents NDM recovery by P12, microglia-specific manipulation of HIF1α or LDHA (via knockout or pharmacological inhibition) could test whether reducing Warburg-like metabolism restores neurodevelopmental transcriptional programs.
The authors should consider discussing whether the observed metabolic changes are intrinsic to microglia or are potentially influenced by BAMs.

The bifurcation between the "chemokine/NF-κB" and "interferon/antiviral" APIM2 trajectories is transcriptionally defined, but the proportion of cells following each trajectory over time (P3–P12) is not quantified. Linking these trajectories to anatomical regions would strengthen the interpretation.

The emergence of Inf_BAM cells is intriguing, but their potential role in cortical inflammation remains unclear. Anatomical localization (meningeal vs. perivascular) using Mrc1/CD206 immunostaining would help clarify their potential influence on cortical microglia.

Additional points

-The number of biological replicates per condition and per time point should be clearly stated.

-Given that neurodevelopmental disorders linked to early life inflammation often show sex-specific outcomes, the authors should clearly indicate the sex of animals used in each experiment, especially in the PLX and LPS models, and specify whether any sex-dependent differences were observed.

-The authors should include representative images of microglia from both control and LPS conditions, indicating brain regions (Figure 5K).

-Some figure citations appear missing throughout the text and should be added to guide readers through the data.

Reviewer #4

(Remarks to the Author)
I co-reviewed this manuscript with one of the reviewers who provided the listed reports. This is part of the Nature Communications initiative to facilitate training in peer review and to provide appropriate recognition for Early Career Researchers who co-review manuscripts.

Version 1:

Reviewer comments:

Reviewer #1

(Remarks to the Author)
The authors did a great job in addressing my concerns. The revised manuscript would be an interest for a broader readership of Nat Commun. I have no further concerns.

Reviewer #2

(Remarks to the Author)
The manuscript is now much clearer and improved. I have no further comments.

Reviewer #3

(Remarks to the Author)
The authors have done a good job addressing the reviewers comments. Overall, I think they have responded well to the concerns raised. The manuscript has been strengthened by the addition of new experimental data, and several conclusions have been revised. In particular, the spatiotemporal dynamics is now clearer and more convincing.

My remaining concern relates to the nomenclature. In my view, the term "neurodevelopment-associated microglia" remains

too broad, as many microglial populations present during development could reasonably be considered neurodevelopment-associated. The proposed acronym therefore does not sufficiently capture the specific features that distinguish this population from other developmental microglial states described in the literature. Given the already large number of acronyms used to define developmental and context-specific microglial populations, a more specific terminology would help avoid confusion and facilitate comparison across studies.

In addition, I found the distinction between the microglial "state" and its molecular signature somewhat unclear throughout the revised manuscript, including in the title. The authors define and discuss a cellular state, but at several points the terminology appears to shift toward describing a molecular or transcriptional program. While these concepts are related, they are not equivalent. A cellular state may be characterized by a particular molecular signature, but the signature itself should not necessarily be interpreted as the state. In this context, I would suggest that the authors consider aligning the terminology more consistently, for example by referring to a microglial state defined by (or associated with) a specific transcriptional/molecular program, rather than using “program”. In this context, the previous wording ("state") seemed conceptually more precise and better aligned with the way the results are presented throughout the manuscript.

Apart from these points, I believe the authors have addressed the major concerns raised during the review process, and the manuscript has been substantially improved.

Reviewer #4

(Remarks to the Author)
I co-reviewed this manuscript with one of the reviewers who provided the listed reports. This is part of the Nature Communications initiative to facilitate training in peer review and to provide appropriate recognition for Early Career Researchers who co-review manuscripts.

Open Access This Peer Review File is licensed under a Creative Commons Attribution 4.0 International License, which permits use, sharing, adaptation, distribution and reproduction in any medium or format, as long as you give appropriate credit to the original author(s) and the source, provide a link to the Creative Commons license, and indicate if changes were made.
In cases where reviewers are anonymous, credit should be given to 'Anonymous Referee' and the source.
The images or other third party material in this Peer Review File are included in the article’s Creative Commons license, unless indicated otherwise in a credit line to the material. If material is not included in the article’s Creative Commons license and your intended use is not permitted by statutory regulation or exceeds the permitted use, you will need to obtain permission directly from the copyright holder.
To view a copy of this license, visit https://creativecommons.org/licenses/by/4.0/

Point-by-point Response to the Reviewers' Comments

We sincerely thank the reviewers for their thoughtful and constructive assessment of our manuscript. Their comments have helped us improve the conceptual clarity of the study and strengthen the supporting evidence. In response, we performed additional experiments and analyses, incorporated new data, and revised the manuscript accordingly. Below, we provide a point-by-point response to each comment. Major changes in the revised manuscript are highlighted in yellow.

Reviewer #1 (Remarks to the Author):

This study by Zhu et al. investigated microglial state and function in early postnatal period of mouse brain development. The author performed single cell transcriptomic profiling of P3, P7, and P12 brain parenchymal microglia, with and without immune perturbation with single dose LPS injection at P2. They uncovered a previously uncharacterized microglial state that they named neurodevelopment-associated microglial state (NMD). Intriguingly, this cluster of microglia increased dramatically during 2nd postnatal week, from 4% at P3 to 11.8% at P7 to 46% at P12. A single dose of LPS injection on P2 alters such developmental trajectory and renders inflammatory microglia transition. To study the function of NMD microglial state, the authors depleted microglia by CSF1R inhibitor PLX5622 injection from P5 to P11. While gross myelination remained unchanged, microglial depletion from P5-11 resulted in increased Gephyrin and PSD-95, reduced dendritic complexity and shorter dendritic length. At functional level, PLX-treated mice demonstrated reduced locomotion and impaired sociability. Microglia show high heterogeneity during brain development and in various diseases, and this study provides an additional layer of understanding in this regard.

Unfortunately, the data presented does not support their overall conclusion, which is "a transient neurodevelopmental microglial state defines a critical period of cortical maturation".

Reviewer comment 1. *First, their end point was P12. It is not possible to conclude NMD state is transient without including more timepoints beyond P12. Specifically, one doesn't know how the NDM state evolves beyond P12. More time points beyond P12 are needed to fully support their conclusion.*

Response: We appreciate this important point. Our original P3–P12 scRNA-seq dataset defined the emergence and peak enrichment of NDM during early postnatal

development, but did not by itself resolve the post-P12 trajectory of this state. In the revised manuscript, we have therefore refined the terminology and added post-P12 evidence to define NDM more precisely as a developmentally restricted postnatal neurodevelopment-associated microglial state, rather than relying on "transient" as a conclusion from the original time course alone.

First, we extended the experimental validation beyond P12 by performing RNAscope analysis for *Numb*, an NDM-enriched marker, together with IBA-1 immunostaining across P3, P7, P12, and P17. This analysis showed that $Numb^{+}$ IBA-1^{+} microglia were present at relatively low proportions at P3 and P7, increased markedly at P12, and declined substantially by P17. Specifically, the proportion of $Numb^{+}$ IBA-1^{+} microglia was 6.3 ± 0.4% at P3, 9.5 ± 1.3% at P7, 50.6 ± 3.6% at P12, and 15.6 ± 0.9% at P17 (Fig. 2G,H). Although *Numb* alone does not define the full NDM transcriptional state, this orthogonal in situ analysis provides post-P12 experimental evidence that an NDM-associated program is strongly enriched around P12 and is reduced by P17.

Second, we extended the developmental comparison to P30 by integrating our dataset with the open-access developmental microglial atlas from Hammond et al.[1]. Among available developmental microglial datasets, this dataset was selected for formal integration because it was generated using a droplet-based single-cell platform and was therefore technically more comparable to our droplet-based SeekOne dataset than plate-based datasets. In this cross-dataset analysis, cells mapped to an NDM-like identity were concentrated within the early postnatal compartment, minimally represented at E14.5, and only sparsely detected at P30. By contrast, P30 microglia mapped predominantly to Mg1-like or homeostatic states (Fig. 2I,J and Fig. S2F, G). These data support the conclusion that NDM is not maintained as a major microglial identity by P30.

Third, to place our findings in the broader developmental microglial literature, we summarized published scRNA-seq atlases that include P21 and later postnatal stages [1–3] in Response Fig. 1 and Response Table 1. Across these datasets, microglia at P21, P30, P60, and adult stages are primarily annotated as homeostatic, maturation-associated, or adult-like populations. Their reported annotations and marker profiles do not support the presence of an NDM-like neurodevelopment-associated state as a dominant microglial identity at these later stages. Because these studies differ in platform, sampling strategy, and cluster nomenclature, we use them as independent contextual support rather than as direct proof of complete NDM absence. Nevertheless, they are consistent with our revised interpretation that NDM is developmentally restricted and is not sustained as a dominant later postnatal or adult-like microglial state.

Together, the original P3–P12 scRNA-seq data, the newly added P17 RNAscope validation, the P30 cross-dataset integration, and later-stage developmental microglial atlases provide convergent support for the refined conclusion that NDM is enriched within a restricted early postnatal developmental window, peaks around P12, and declines thereafter rather than persisting as a major microglial identity into later postnatal maturation.

Accordingly, we have revised the manuscript to avoid using "transient" beyond the temporal resolution of the original P3–P12 scRNA-seq dataset. We now describe NDM as a temporally restricted early postnatal neurodevelopment-associated microglial state. These revisions have been incorporated into the Results section 2.3 (lines 206-220), the Discussion (lines 497–506 and 536-541), and the Methods (lines 590–608 and 752–762) of the revised manuscript.

Response Table 1. Summary of published developmental microglial single-cell atlases covering P21, P30 and P60 stages.

Study	Stage beyond P12	Main reported microglial populations	Conclusion for NDM
Masuda et al., Nature 2019	P21<br><br>Adult (16 weeks)	Juvenile and adult microglia were resolved into time- and region-dependent postnatal clusters, including C7–C10-related populations. Postnatal microglia showed Cst3/Sparc-associated signatures, while adult microglia displayed a more homogeneous regional distribution than juvenile microglia.	No dominant NDM-like neurodevelopment-associated population was reported at P21 and adult stages.
Hammond et al., Immunity 2019	P30<br><br>P100	Juvenile (P30) and adult (P100) microglia largely overlapped within three central clusters, 7a–7c, which lacked distinct cluster-specific marker genes.	No dominant NDM-like neurodevelopment-associated population was reported at P30 and P100.
Li et al., Neuron 2019	P60	Adult microglia were almost exclusively distributed in two clusters, cluster 0 and cluster 6. Both expressed homeostatic microglial signature genes, and cluster 6 differed from cluster 0 by only 27 genes, many of which were immediate early genes.	No dominant NDM-like neurodevelopment-associated population was reported at P60.
Overall	P21–P100	Later-stage microglia mainly converge toward homeostatic/adult-like states.	NDM is not reported as a sustained dominant later-stage microglial identity.

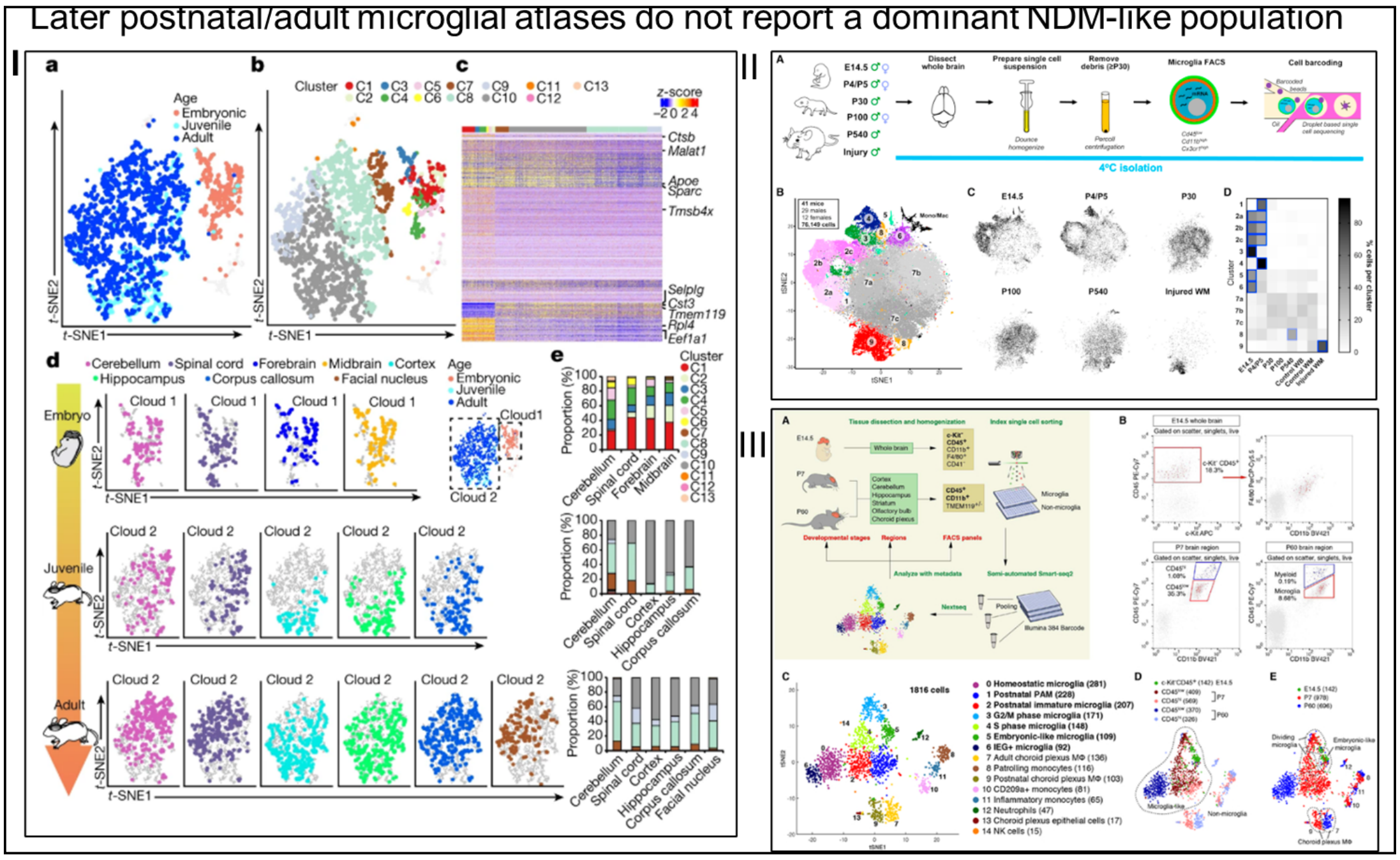

Response Fig. 1. Published developmental microglial scRNA-seq atlases support the absence of a dominant NDM-like population at later postnatal stages. Representative panels are shown from I, Masuda et al., *Nature* 2019, covering juvenile P21 microglia; II, Hammond et al., *Immunity* 2019, covering juvenile P30 and adult P100 microglia; and III, Li et al., *Neuron* 2019, covering adult/P60 microglia.

Ref

[1]. Hammond, Timothy R et al. "Single-Cell RNA Sequencing of Microglia throughout the Mouse Lifespan and in the Injured Brain Reveals Complex Cell-State Changes." *Immunity* vol. 50,1 (2019): 253-271.e6.

[2]. Li, Qingyun et al. "Developmental Heterogeneity of Microglia and Brain Myeloid Cells Revealed by Deep Single-Cell RNA Sequencing." *Neuron* vol. 101,2 (2019): 207-223.e10.

[3]. Masuda, Takahiro et al. "Spatial and temporal heterogeneity of mouse and human microglia at single-cell resolution." *Nature* vol. 566,7744 (2019): 388-392.

Reviewer comment 2. *Second, PLX depletes all microglia. NMD cluster only represents 11.8% at P7 and 46% at P12. It is inappropriate to conclude circuit maturation impairment is due to NMD state alone.*

Response: We appreciate this important point. PLX5622-mediated CSF1R inhibition broadly depletes microglia and does not selectively ablate NDM. We therefore do not interpret the PLX5622 phenotypes as evidence that loss of NDM alone is sufficient to impair cortical circuit maturation. In the revised manuscript, we have clarified this distinction while preserving the functional relevance of the developmental window during which NDM emerges and becomes enriched.

The P5–P11 PLX5622 depletion paradigm was designed to test whether microglia are required during the early postnatal period when NDM and Mg1 expand, rather than to perform an NDM-specific manipulation. This window overlaps with the transition from low NDM representation at P7 to prominent NDM enrichment by P12, but it also affects Mg1 and other early postnatal microglial states. Accordingly, we now frame the PLX5622 experiment as evidence that broad depletion of microglia during this

developmental window disrupts cortical maturation-associated outcomes, rather than as direct proof of NDM-specific causality.

We have revised the Abstract, Results section 2.4 (lines 227–231), and Discussion (lines 508-509 and 541-545) to make this interpretation explicit. In the revised text, PLX5622 treatment from P5 to P11 is described as depleting early postnatal microglia during the period when NDM and Mg1 expand. This manipulation spared gross myelination but was associated with altered synaptic protein composition, reduced dendritic complexity, reduced Ctip2^{+} layer 5 neuronal density in the primary somatosensory cortex, and later behavioral alterations, which are now interpreted cautiously in light of locomotor effects. We now avoid attributing these phenotypes specifically to NDM loss.

This revision clarifies the intended functional interpretation without overextending the conclusion: the single-cell analyses identify NDM as a developmentally restricted neurodevelopment-associated microglial state, while the PLX5622 experiment demonstrates that microglia are functionally important during the same early postnatal window. We also note in the revised Discussion that selective testing of NDM itself will require future state-resolved or inducible perturbation strategies, because validated tools for selectively manipulating NDM without affecting other early postnatal microglial states are currently lacking.

Reviewer comment 3. *The behavior analysis showed impaired locomotor activity in PLX group (Fig. 4I), which makes it hard to tease out the effect of reduced locomotor activity in anxiety/social behaviors (similar issue for the LPS group compared to control, especially with myelination phenotype).*

Response: We thank the reviewer for raising this important point. Reduced locomotor activity is an important consideration for exploration-dependent behavioral assays, particularly when interpreting elevated zero maze and three-chamber outcomes. In the revised manuscript, we have therefore adjusted both the behavioral readouts and the language used to interpret these data, while keeping the behavioral findings in their appropriate role as downstream phenotypic outcomes rather than the primary mechanistic evidence.

For the PLX5622 experiment, reduced locomotion could directly influence distance-based measurements in the elevated zero maze. We therefore replaced open-arm distance with open-arm time as the primary open-arm readout in the revised manuscript (Fig. 4F). We also revised the corresponding text to avoid interpreting this assay as direct evidence of anxiety-like behavior. The elevated zero-maze result is

now described more specifically as reduced locomotor activity accompanied by reduced open-arm time.

We also revised the interpretation of the three-chamber assay. Because this assay measures the relative allocation of interaction time between an unfamiliar mouse and an empty target, it is not solely a total-distance readout. Nevertheless, reduced locomotion or exploratory drive may influence task performance. We therefore no longer describe these findings as definitive evidence of impaired sociability. Instead, we report them more precisely as reduced interaction with the unfamiliar mouse and a reduced social preference index following early postnatal microglial depletion.

Importantly, the behavioral results are not used as the primary basis for the functional interpretation of the PLX5622 experiment in the revised manuscript. They are presented as later behavioral alterations associated with early postnatal microglial depletion. The conclusion that early postnatal microglia contribute to cortical maturation is supported primarily by convergent molecular and structural evidence, including altered synaptic protein composition, reduced dendritic complexity, and reduced Ctip2^{+} layer 5 neuronal density in the primary somatosensory cortex.

We applied the same interpretive caution to the LPS model. For the elevated zero maze, we used open-arm time rather than open-arm distance as the primary open-arm readout (Fig. 5B), and we revised the text to avoid presenting the behavioral results as direct evidence of anxiety-like or social deficits. We also clarified that later behavioral alterations after neonatal LPS exposure should not be attributed solely to reduced MBP expression or myelination-related changes. Instead, they are now described as behavioral alterations associated with broader neurodevelopmental changes following neonatal inflammation, including reduced MBP expression.

These revisions have been incorporated into the revised Results section, as indicated in lines 266-274 and 282–284 of the revised manuscript.

Reviewer comment 4*. To elucidate whether second postnatal week involves region-specific changes in microglial density and maturation, the authors performed IHC of IBA1 and PRRY12 across cortex, hippocampus, corpus callosum, and striatum. The whole paper was about NDM expansion and function, it would be desirable to include some markers that defines NDM cluster instead of pan-microglia (IBA1) and homeostatic microglial (P2RY12) markers.*

Response: We thank the reviewer for this constructive suggestion. The original IBA-1 and P2RY12 analyses were intended to assess overall microglial abundance and canonical homeostatic maturation across brain regions. However, we agree that these

markers do not directly report the NDM-associated program, which is central to the manuscript. In the revised study, we therefore added an in situ analysis of an NDM-enriched transcript to complement the pan-microglial and homeostasis-associated markers.

Specifically, we performed RNAscope in situ hybridization for *Numb*, a representative NDM-enriched transcript associated with the neurodevelopment-related gene program, combined with IBA-1 immunostaining to identify microglia. This approach allowed us to quantify *Numb*$^+$ IBA-1$^+$ microglia across postnatal stages and selected brain regions, providing an orthogonal tissue-level readout of microglia expressing an NDM-associated transcript. We have also clarified in the revised manuscript that this represents marker-level validation of an NDM-associated program in situ, rather than definitive reconstruction of the full transcriptomic NDM state from tissue sections.

The new RNAscope analysis showed that *Numb*$^+$ IBA-1$^+$ microglia were present at low proportions at P3 and P7, increased markedly at P12, and declined by P17 (Fig. 2G,H). This temporal pattern is consistent with the early postnatal enrichment of the NDM-associated program identified by scRNA-seq and supports the interpretation that *Numb*-defined NDM-like microglia are most prominent within a restricted early postnatal window.

We further examined the regional distribution of *Numb*$^+$ IBA-1$^+$ microglia at P12 in the primary somatosensory cortex (S1), hippocampus, and striatum. *Numb*$^+$ IBA-1$^+$ microglia were detected across all three regions, with no significant enrichment in S1 (Fig. 4C, D). We have incorporated this finding into the revised interpretation of the PLX5622 experiment. Specifically, the preferential structural alterations observed in S1 do not appear to be explained simply by a higher local abundance of *Numb*-expressing NDM-like microglia. Rather, these findings suggest that S1 may be particularly sensitive to microglial perturbation during a period of active dendritic and synaptic maturation.

These revisions have been incorporated into the Results sections (lines 206-220 and 258-264), the Discussion (lines 512-519), and the Methods (lines 590–608) of the revised manuscript.

Reviewer comment 5. *There are several published microglia datasets (Hammond, Timothy R., et al. Immunity 50.1 (2019): 253-271; Li, Qingyun, et al. Neuron 101.2 (2019): 207-223; Matcovitch-Natan et al, Science 2016) that include similar (Matcovitch-Natan et al. P3, P6, and P9; Hammond et al. Immunity iP4, and Li, Qingyun, et al. iP7) and additional timepoints (P20, P30). The authors should integrate*

different datasets to support their conclusion.

Response: We thank the reviewer for this valuable suggestion. We agree that comparison with published developmental microglial datasets provides an important external framework for evaluating the temporal interpretation of NDM, particularly whether the NDM-associated program is preferentially linked to early postnatal development or persists as a major microglial identity at later postnatal stages.

In the revised manuscript, we addressed this point using a two-level approach. First, we performed formal cross-dataset integration with the developmental microglial atlas from Hammond et al., which includes embryonic, early postnatal, and later postnatal microglia, including E14.5, P4/P5, and P30[1]. Among the datasets suggested by the reviewer, this dataset was the most suitable for direct co-embedding and label-transfer analysis because it was generated using a droplet-based single-cell platform, making it technically more comparable to our droplet-based SeekOne dataset.

This integration supported the developmental restriction of the NDM-associated program. Cells mapped to an NDM-like identity were concentrated within the early postnatal compartment, were minimally represented at E14.5, and were only sparsely detected at P30. By contrast, P30 microglia mapped predominantly to Mg1-like or homeostatic microglial states (Fig. 2I,J and Fig. S2F,G). These findings are consistent with our own P3–P12 time-series data and support the conclusion that NDM is preferentially associated with a restricted early postnatal window rather than being maintained as a dominant microglial identity at later postnatal stages.

We also considered the Li et al. and Matcovitch-Natan et al. datasets as independent developmental references[2-3]. Because these datasets were generated using Smart-seq2 and MARS-seq, respectively, rather than droplet-based platforms, we did not merge them into the same integrated object. Instead, we summarized their relevant developmental time points, reported microglial populations, and later-stage annotations in Response Table 1 and Response Fig. 1. The Matcovitch-Natan et al. dataset is particularly useful for contextualizing early microglial maturation before P12, whereas the Li et al. dataset provides additional reference information across early postnatal and later stages.

Across these published developmental atlases, later postnatal microglia at P20/P21, P30, P60, and adult stages are mainly annotated as homeostatic, maturation-associated, or adult-like populations. Their reported annotations and marker profiles do not support NDM as a dominant neurodevelopment-associated microglial identity at these later stages. We have summarized these cross-dataset observations in the revised response and have also added this point to the Discussion to better contextualize the temporal

restriction of NDM in relation to published developmental microglial atlases.

Together, the formal integration with the Hammond et al. atlas and the broader reference-based comparison with the Li et al. and Matcovitch-Natan et al. datasets strengthen our conclusion that NDM represents a developmentally restricted early postnatal neurodevelopment-associated microglial state. These analyses have been incorporated into Results section 2.3 (lines 213–220) and the Methods section (lines 752–762), with supporting summaries provided in Response Fig. 1 and Response Table 1.

Ref:
[1]. Hammond, Timothy R et al. "Single-Cell RNA Sequencing of Microglia throughout the Mouse Lifespan and in the Injured Brain Reveals Complex Cell-State Changes." *Immunity* vol. 50,1 (2019): 253-271.e6.
[2]. Li, Qingyun et al. "Developmental Heterogeneity of Microglia and Brain Myeloid Cells Revealed by Deep Single-Cell RNA Sequencing." *Neuron* vol. 101,2 (2019): 207-223.e10.
[3]. Masuda, Takahiro et al. "Spatial and temporal heterogeneity of mouse and human microglia at single-cell resolution." *Nature* vol. 566,7744 (2019): 388-392.

Reviewer comment 6. *Fig 4C appears to show more dendritic spines in PLX treated group. This data should be quantified and discussed in the result section.*

Response: We thank the reviewer for this careful observation. We agree that the original representative image could give the impression of altered dendritic spine abundance in the PLX5622-treated group. After re-examining the dataset, we recognized that this image was not appropriate for supporting or excluding a conclusion about dendritic spine density, because the Golgi–Cox experiment in this study was designed and acquired for dendritic arbor reconstruction rather than dedicated spine quantification.

To avoid potential overinterpretation, we have removed the original representative image and the corresponding descriptive text from the revised manuscript. We have also restricted the Results to the morphological endpoints that were systematically quantified, including neuronal reconstruction, Sholl analysis, dendritic complexity, and total dendritic length. These analyses support the conclusion that early postnatal microglial depletion alters dendritic arbor maturation, particularly in the primary somatosensory cortex, but we do not draw conclusions regarding dendritic spine density or spine maturation.

We also note that previous studies have reported that developmental microglial perturbation can affect spine- or synapse-related features across different brain regions, developmental stages, and experimental paradigms[1–3]. Thus, this is a biologically relevant direction; however, in the present study, we have limited our

conclusion strictly to the systematically quantified dendritic arborization phenotypes. The corresponding revisions have been incorporated into Results section 2.4 (lines 246-253) and revised Fig. 3H–K of the revised manuscript.

Ref:
[1]. Paolicelli, Rosa C et al. "Synaptic pruning by microglia is necessary for normal brain development." *Science (New York, N.Y.)* vol. 333,6048 (2011): 1456-8.
[2]. Favuzzi, Emilia et al. "GABA-receptive microglia selectively sculpt developing inhibitory circuits." *Cell* vol. 184,15 (2021): 4048-4063.e32.
[3]. Ma, Xiaokuang et al. "Depletion of microglia in developing cortical circuits reveals its critical role in glutamatergic synapse development, functional connectivity, and critical period plasticity." *Journal of neuroscience research* vol. 98,10 (2020): 1968-1986.

Reviewer comment 7. *The method section indicated both male and female pups were included. However, no data was shown on both sexes in Fig. 3-5. Literature has demonstrated sex dimorphism in microglial function, especially in setting of immune perturbation. It is important to show the data in the result sections.*

Response: We thank the reviewer for raising this important point. Sex is an important biological variable in microglial biology, particularly during development and in the context of early-life immune perturbation. In the revised manuscript, we have updated the relevant data displays to indicate the sex of each individual animal directly in the statistical plots for the PLX5622 and LPS experiments, including Figs.2-7 and the corresponding supplementary figures. We have also clarified the sex composition of the experimental cohorts in the figure legends and Methods.

After annotating individual data points by sex, we did not observe an obvious sex-dependent separation across the measured outcomes in these experiments. However, the present study was not designed or statistically powered to perform formal sex-stratified analyses. We therefore do not make sex-specific claims or draw post hoc conclusions regarding sex-dependent effects from these datasets. Instead, the revised figures allow the distribution of male and female animals to be assessed directly while keeping the interpretation aligned with the experimental design.

We have explicitly stated this limitation in the revised Discussion (lines 549–554) and updated the relevant figure legends and Methods accordingly.

Reviewer comment 8. *Lines 326-329: "Following LPS exposure, these developmental and homeostatic states contracted sharply (NDM ~3%, Mg1 ~2%), whereas the recovery-phase inflammatory state RPIM expanded (~49%), indicating a shift from developmental/homeostatic to inflammatory programs." Is the actual cell number of NDM also different? A decrease in the NDM percentage may be due to excessive*

proliferation of PRIM.

Response: We appreciate the reviewer's point regarding compositional effects in scRNA-seq data. A reduction in the relative proportion of NDM does not, by itself, demonstrate an absolute reduction in transcriptome-defined NDM cell number.

To address this concern, we added orthogonal tissue-level analyses at P12. First, quantification of total IBA-1$^+$ microglial density showed no significant difference between NS and LPS groups (Fig. S4A–C). This argues against gross expansion of the total IBA-1+ microglial population as the sole explanation for the reduced relative representation of NDM in the scRNA-seq dataset.

Second, we performed RNAscope analysis for *Numb*, a representative NDM-enriched transcript associated with the neurodevelopment-related program, combined with IBA-1 immunostaining. This analysis showed that the proportion of *Numb*$^+$ IBA-1$^+$ microglia among total IBA-1$^+$ cells was significantly reduced after LPS exposure (NS, 50.6 ± 3.6%; LPS, 19.7 ± 1.6%; Fig. 5L, M). Thus, neonatal LPS exposure reduced the fraction of IBA-1$^+$ microglia expressing an NDM-associated transcript at the tissue level.

We have revised the Results to avoid inferring absolute NDM cell loss solely from scRNA-seq proportions. Instead, we now describe the LPS-induced phenotype as a marked reduction in the relative representation of NDM in the scRNA-seq dataset, supported by an independent tissue-level reduction in the fraction of *Numb*$^+$ IBA-1$^+$ microglia. Together, the unchanged total IBA-1$^+$ microglial density and reduced *Numb*$^+$ IBA-1$^+$ fraction argue against a purely compositional explanation driven only by RPIM expansion, and support a reduction of NDM-associated features within the IBA-1$^+$ microglial population after neonatal LPS exposure.

The Results section has been revised accordingly to clarify this interpretation **(**lines 303 and lines 323-335**)**.

Reviewer #2 (Remarks to the Author):

Zhu et al., provide an interesting manuscript that does extensive profiling of microglial states in the developing brain, highlighting the second postnatal week in mice as an underappreciated and thus understudied developmental window. I agree with this sentiment and the authors should be lauded for their detailed analysis of this large

sequencing dataset and for integrating many aspects of microglial biology (metabolism, development, inflammation) in their studies. The microglia populations that they identify are indeed novel and will likely provide valuable context to the field. These details aside, the article suffers significantly from a lack of strong focus, disorganized/hard to interpret figures, and little/no concrete identification and tracking of the microglia "subsets" they identified outside of sequencing. If not rectified, these issues will significantly detract from the potential influence their findings might have on the field.

Major Comments:

Reviewer comment 1. *The bioinformatics, while impressive, is extremely overwhelming visually and hard to follow. While it is appreciated that many of the same visualizations and analyses are performed from figure to figure (for consistency), some analyses are unnecessary to the main points of the paper, and the dense organization of the figures makes it difficult and unpleasant to read and properly interpret. Below in minor comments are my suggested changes.*

Response: We thank the reviewer for acknowledging the scope of the bioinformatics analyses and for highlighting the importance of clearer visual organization. We agree that, in the original submission, the density of some figure panels and the repetition of supporting computational analyses could obscure the main biological message. In the revised manuscript, we have substantially reorganized the main and supplementary figures to improve readability, logical flow, and interpretability.

Specifically, we moved repetitive or supporting computational analyses to the Supplementary Information, removed non-essential panels, and simplified the main figures so that each figure now more directly supports a defined biological conclusion. We also revised the figure legends to clarify the purpose of each panel and improve consistency across the manuscript. These changes sharpen the main narrative around three central elements: the developmental emergence and in situ validation of NDM, the consequences of early postnatal microglial depletion during the window in which NDM emerges and expands, and the sequential APIM/RPIM remodeling induced by neonatal LPS exposure.

We address the reviewer's panel-specific suggestions in the corresponding responses below.

Reviewer comment 2. *Moreover, the current layout of the manuscript emphasizes the importance of NDMs in the first half, then APIMs in the second half, and never produces any strong evidence that these cell subtypes are in any way linked to one another.*

Response: We thank the reviewer for this important comment. In the revised manuscript, we reorganized the LPS-related Results to clarify how the NDM and APIM/RPIM analyses are connected within the same developmental–inflammatory framework. We first define NDM as a physiological microglial program normally enriched at P12, and then show that neonatal LPS attenuates this program at the same developmental stage. Thus, the P12 analysis serves as the critical endpoint at which neonatal inflammation is associated with impaired establishment of the NDM/Mg1-dominated developmental microglial landscape.

We next asked what microglial changes preceded this altered P12 endpoint. In revised section 2.6, we integrated LPS-exposed microglia across P3, P7, and P12 and identified a temporally ordered APIM/RPIM remodeling pattern. APIM states dominated the acute P3 response, whereas RPIM states emerged from P7 onward and became predominant by P12. Importantly, RPIM3 partially re-expressed selected neurodevelopment-associated transcripts but did not restore the full NDM-associated and homeostatic program. This analysis positioned APIM/RPIM as LPS-associated microglial states that precede and accompany the impaired establishment of the normal P12 NDM/Mg1 landscape, rather than as an unrelated inflammatory module.

We then focused on the earliest stage of microglial remodeling after LPS in revised sections 2.7 and 2.8. At P3, before the normal P7–P12 expansion of the NDM-associated program, the acute LPS response was dominated by APIM2. APIM2 accounted for the majority of parenchymal microglia and was characterized by glycolytic, ATP metabolic, and lactate-associated features, rather than by a uniform classical inflammatory program. Thus, the significance of the APIM2 analysis is that it identifies the earliest dominant microglial response to neonatal inflammation and shows that microglia are already strongly reshaped in an immunometabolic direction before the physiological NDM program is normally established.

Accordingly, APIM2 provides early temporal and immunometabolic context for the later P12 microglial landscape characterized by RPIM predominance and NDM attenuation. In this revised structure, APIM/RPIM are integrated into the same

developmental–inflammatory framework as NDM: neonatal inflammation is first associated with an APIM2-dominated acute immunometabolic response, followed by the emergence and predominance of RPIM states, coinciding with impaired establishment of the normal NDM/Mg1-dominated P12 program. These revisions have been incorporated into revised Results sections 2.5–2.8.

Reviewer comment 3. *The identification of "NDM" and "APIM1/2" microglia subsets would be strengthened by additional molecular analyses other than sequencing. Is there some way in saline/control mice to visualize these two groups of microglia at P12? (i.e. some marker, either an antibody or FISH probe, that is $^{+}$ for NDMs, - for Mg1s) so you could histologically determine their specific contributions to neurodevelopment and potentially begin to assign some functional significance to these subsets.*

Response: We appreciate the reviewer's constructive suggestion. In the revised manuscript, we added tissue-level molecular validation for the scRNA-seq-defined programs in the biological contexts in which they were detected.

For NDM, we performed RNAscope in situ hybridization for *Numb*, a representative NDM-enriched transcript associated with the neurodevelopment-related program, combined with IBA-1 immunostaining to identify microglia. In saline-treated control brains, *Numb*$^{+}$ IBA-1^{+} microglia were present at low proportions at P3 and P7, increased markedly at P12, and declined by P17 (Fig. 2G,H). We also applied this assay at P12 after neonatal LPS exposure and found that the proportion of *Numb*$^{+}$ IBA-1^{+} microglia was significantly reduced compared with saline controls (NS, 50.6 ± 3.6%; LPS, 19.7 ± 1.6%; Fig. 5L, M). These data provide orthogonal in situ support for the temporal enrichment of an NDM-associated program and its reduction after neonatal inflammation.

For APIM1/2, we clarified their biological context in the revised manuscript. These states were not identified as baseline populations in saline-treated P12 brains; rather, they emerged after neonatal LPS exposure and were most prominent during the acute inflammatory phase at P3. We therefore validated APIM2-associated molecular features in that relevant context. Specifically, we added immunostaining for the glycolysis-associated proteins PGAM1 and PGK1, reflecting the glycolytic program enriched in APIM2. After neonatal LPS exposure at P3, PGAM1 and PGK1 signals increased in IBA-1^{+} microglia; in S1, PGK1^{+} IBA-1^{+} microglia were significantly increased, whereas PGAM1^{+} IBA-1^{+} microglia showed an upward trend (Fig. 7F, G).

These findings support APIM2-associated glycolytic features in tissue during the acute inflammatory phase.

We have clarified that these marker-based assays provide in situ validation of state-associated molecular features, rather than definitive histological reconstruction of the full transcriptomic states or proof of state-specific functional causality. These data and clarifications have been incorporated into the revised Results (lines 206–212, 329–335, and 449-455) and Methods (lines 590–608), and the need for state-specific manipulation tools is now discussed in the revised Discussion (lines 536-541).

Reviewer comment 4. *The authors repeatedly refer to NDMs as a "a transient, second week-dominant neurodevelopment-associated microglial state", but they never look for these cells past P12. How long does this microglia population last? Do they die off, or develop into MG1 "homeostatic" mature microglia after a set amount of time? You cannot state that they are transient without showing that they disappear shortly after appearing.*

Response: We thank the reviewer for raising this important and constructive point. This comment prompted us to refine both the terminology used to describe NDM and the evidentiary basis for its post-P12 dynamics. In the revised manuscript, we have added post-P12 experimental validation, incorporated later-stage developmental reference datasets, and clarified that our conclusions concern population-level dynamics of the NDM-associated program rather than direct lineage fate of individual NDM cells.

Our original P3–P12 scRNA-seq time course defined the emergence and peak enrichment of NDM during early postnatal development, but did not by itself determine how this state evolves after P12. We have therefore revised the manuscript to avoid using "transient" or "second week-dominant" as stand-alone conclusions based solely on the original scRNA-seq dataset. Instead, we now describe NDM more precisely as a temporally restricted early postnatal neurodevelopment-associated microglial state.

First, we extended our experimental validation beyond P12 by performing RNAscope in situ hybridization for *Numb*, a representative NDM-enriched transcript associated with the neurodevelopment-related program, combined with IBA-1 immunostaining across P3, P7, P12, and P17. This analysis showed that the proportion of *Numb*$^+$ IBA-1$^+$ microglia was low at P3 and P7 (6.3 ± 0.4% and 9.5 ± 1.3%, respectively), increased

markedly at P12 (50.6 ± 3.6%), and declined by P17 (15.6 ± 0.9%; Fig. 2G,H). Although *Numb* alone does not define the full NDM transcriptional state, these new in situ data provide post-P12 experimental evidence that an NDM-associated program is enriched around P12 and substantially reduced by P17.

Second, we extended the developmental analysis to P30 by integrating our dataset with the open-access developmental microglial atlas from Hammond et al.[1], which includes E14.5, P4/P5, and P30 microglia. We selected this dataset for direct integration because it was generated using a droplet-based single-cell platform and is therefore technically more comparable to our droplet-based SeekOne dataset. In this cross-dataset analysis, cells mapped to an NDM-like identity were concentrated within the postnatal developmental compartment, were minimally represented at E14.5, and were only sparsely detected at P30. In contrast, P30 microglia mapped predominantly to Mg1-like or homeostatic states (Fig. 2I,J and Fig. S2F, G). This later-stage analysis supports the interpretation that the NDM-associated program is not maintained as a major microglial identity by P30.

Third, we reviewed additional published developmental microglial scRNA-seq atlases that include P21 and later stages, and summarized the relevant evidence in Response Fig. 1 and Response Table 1. Across these studies, P21, P30, P60, and adult microglia were mainly assigned to homeostatic, maturation-associated, or adult-like clusters. We did not find evidence in their reported annotations or signatures for a dominant NDM-like neurodevelopment-associated microglial population at P21 or later stages. Because these datasets differ in platform, sampling design, and cluster nomenclature, we use them as supportive later-stage reference evidence rather than as direct proof of complete NDM absence. Nevertheless, they provide independent contextual support that NDM is not sustained as a dominant microglial identity during later postnatal maturation.

Together, our P3–P12 scRNA-seq data, newly added P17 RNAscope validation of *Numb*, P30 cross-dataset integration, and published developmental atlases covering P21 and later stages provide convergent evidence that NDM is enriched within a restricted postnatal developmental window, peaks around P12, and declines thereafter. These findings support the conclusion that NDM represents a developmentally restricted postnatal neurodevelopment-associated microglial state, rather than a mature or adult-like microglial identity sustained into later postnatal or adult stages.

Regarding the reviewer's question of whether NDM cells are eliminated, transition into Mg1/homeostatic microglia, or enter other microglial states during later maturation, this is an important biological question that requires a different experimental framework. The analyses added in this revision define the temporal dynamics of the NDM-associated program at the population level, but do not establish the lineage fate of individual NDM cells. Resolving this question would require prospective labeling, lineage tracing, longitudinal state tracking, or temporally controlled fate-mapping approaches, which are beyond the scope of the present study and represent an important focus for future work. We have now explicitly clarified this point in the revised Discussion.

These revisions have been incorporated into the Results section 2.3 (lines 206-220), the Discussion (lines 497–506 and 536-541), and the Methods (lines 590–608 and 752–762) of the revised manuscript.

Reviewer comment 5. *The manuscript does not do a good job of properly justifying analyses in certain brain regions. Are these novel subtypes identified present in equal proportions across cortical, subcortical, and hippocampal regions?*

Response: We appreciate the reviewer's point regarding the regional interpretation of the newly identified microglial states. Because our scRNA-seq analysis was performed using whole-brain $CD45^+$ cells, it does not provide region-resolved quantification of microglial state composition. We have clarified this limitation in the revised manuscript and added tissue-level regional validation of an NDM-associated marker.

Specifically, we performed RNAscope in situ hybridization for *Numb*, a representative NDM-enriched transcript associated with the neurodevelopment-related program, combined with IBA-1 immunostaining. We quantified $Numb^+$ IBA-1^+ microglia at P12 in three representative regions: the primary somatosensory cortex (S1), hippocampus, and striatum, corresponding to cortical, hippocampal, and subcortical compartments.

This analysis showed that $Numb^+$ IBA-1^+ microglia were detectable across all three examined regions at P12, without significant enrichment in S1 relative to the hippocampus or striatum (Fig. 4C, D). Thus, within the regions examined, microglia expressing this NDM-associated transcript were broadly detected rather than selectively enriched in the region showing the most prominent structural phenotype after microglial depletion.

We have revised the interpretation accordingly. The preferential S1 phenotype is unlikely to be explained simply by higher local abundance of *Numb*-expressing microglia. Instead, it more likely reflects heightened sensitivity of S1 circuits to early postnatal microglial perturbation during a period of active dendritic and synaptic maturation. This interpretation is consistent with the revised dendritic morphology analysis, which showed that early postnatal microglial depletion reduced dendritic complexity and total dendritic length in S1, whereas comparable changes were not detected in hippocampal or striatal neurons (Fig. 3H–K).

These new RNAscope data and clarifications have been incorporated into Results section 2.4 (lines 246-264), the corresponding figure legends, and the Discussion (lines 508-519) of the revised manuscript.

Reviewer comment 6. *Several questions related to experimental design need to be more explicitly addressed, including: How many mice per group? How many litters per group? Male/Female distribution of each group?*

Response: We thank the reviewer for pointing out the need for clearer reporting of the experimental design. In the revised manuscript, we have updated the figure legends and Methods to explicitly state the number of animals per group, the number of independent litters where applicable, and the male/female distribution of the experimental cohorts.

For the PLX5622 and LPS experiments, we have also updated the relevant data plots so that the sex of each individual animal is indicated directly in the data displays, including Figs. 2–7 and the corresponding supplementary figures. This allows the distribution of male and female animals to be assessed transparently alongside the group-level results.

After annotating the data by sex, we did not observe an obvious sex-dependent separation across the measured outcomes. However, the present study was not designed or statistically powered for formal sex-stratified analyses. We therefore do not make sex-specific claims or infer sex-dependent effects from these datasets. This limitation is now explicitly stated in the revised Discussion (lines 549–554), and the relevant figure legends and Methods have been revised accordingly.

Minor Comments:

We appreciate the reviewer's careful reading and helpful suggestions. We have revised the manuscript accordingly, as detailed below.

1）.Some mention of the LPS condition should be made in figure 1a schematic, so that it is clear to the reader that all data was collected together (important for future comparative analyses).

Response: Fig. 1A has been updated to include the LPS condition and dose (5 mg/kg), clarifying that saline- and LPS-treated samples were collected within the same overall experimental framework.

2). The UMAP in 1C should be labelled as "Saline" treated

Response: The corresponding UMAP panel, now shown as Fig. 1B in the revised manuscript, has been labelled as "Control microglia (Saline)" to make the treatment condition explicit.

3).Fig 1H volcano plot PEM vs MG1 was not mentioned in the text. Is this visualization necessary to the ultimate conclusions of the paper? If not, it should be removed.

Response: The original PEM versus Mg1 volcano plot was not essential to the main conclusions and has been removed from the revised main figure to simplify the presentation and improve readability.

4).The schematic in 1L makes it look like NDMs are the most mature microglia – is this really the case the authors are trying to make? If so, are they really transient?

Response: The original schematic could inadvertently imply that NDM represents the most mature or terminal microglial state, which was not our intended interpretation. Our data support NDM as a developmentally restricted postnatal neurodevelopment-associated microglial state, rather than a terminally mature or end-stage identity. To avoid this potential misinterpretation, the original schematic has been removed, and the manuscript terminology has been revised to avoid implying a linear maturation endpoint.

5).Fig 2C - a title like "pseudo time clusters" would be informative and make the figure more accessible for readers not familiar with monocle.

Response: We have added the title "Pseudotime clusters across microglial states" to Fig. 2C to clarify the purpose of the panel and make the Monocle-based analysis more accessible.

6). Fig 2D - letter in the panel is far from the raster plot.

Response: The panel label in Fig. 2D has been adjusted so that it is properly aligned with the raster plot.

7). Labels providing context to each of the subplots in 2E would also be helpful (i.e. group them as you describe in the text, "Cell cycle markers" "Neurodevelopment Factors". Apoe and SPP1 are not even mentioned in the text – what is their significance to warrant being included in this set of plots?

Response: The original Fig. 2E was not sufficiently focused on the main biological interpretation and included genes, such as Apoe and Spp1, whose relevance to the surrounding text was not clearly explained. Rather than simply adding subgroup labels, we replaced this panel with a new transcription factor activity analysis in Fig. 2E, comparing candidate regulatory differences between Mg1 and NDM. This revised panel more directly supports the interpretation of regulatory programs associated with the bifurcation between homeostatic-like Mg1 and NDM. Expanded transcription factor activity analyses across microglial states and developmental stages are now shown in Supplementary Fig. S2A–C, and the corresponding Results text has been revised accordingly (lines 192-196).

8). Max intensity projection images of the histology in 2I would be appreciated. Additionally, a quantification of the % microglia positive for these markers would help with major concern 3 above.

Response: The histology panel has been revised accordingly. In the revised manuscript, we replaced the original panel with RNAscope in situ hybridization for *Numb*, a representative NDM-enriched transcript associated with the neurodevelopment-related gene program, combined with IBA-1 immunostaining to identify microglia.

The representative images are now shown as maximum-intensity projections generated from confocal z-stacks, providing clearer visualization of RNA puncta within IBA-1^+ microglia. We also quantified the proportion of *Numb*$^+$ IBA-1^+ microglia among total IBA-1^+ cells across developmental stages. This analysis showed that *Numb*$^+$ IBA-1^+ microglia increased around P12 and declined thereafter, providing orthogonal in situ validation of NDM-associated marker expression beyond sequencing. The representative images and quantifications have been incorporated into the revised manuscript (Fig. 2G-H; lines 206-212 and 590-608).

9). Details about the western blot synapse data should be made more clear in the text and main figure. In fact, I can find no details about how the tissues were collected for this experiment. Was this whole brain tissue? How can the authors address the fact

that these synaptic proteins are expressed at different levels across different brain regions?

Response: The tissue source and interpretive scope of the western blot analysis have been clarified in the revised manuscript. We now state in the Results, Methods, and figure legend that the western blot analysis was performed using whole-brain bulk tissue lysates. Whole brains were collected from individual animals at P21 and homogenized for protein extraction before western blotting.

We have also clarified that these bulk western blot data should be interpreted as an initial whole-brain biochemical assessment of synaptic protein changes, rather than as region-specific evidence. Because synaptic proteins can differ across brain regions, we do not use the western blot results alone to support conclusions regarding regional vulnerability. Instead, the regional interpretation is based on subsequent histological and morphological analyses directly comparing S1, hippocampus, and striatum, which showed reduced dendritic complexity and total dendritic length in S1 but not in hippocampal or striatal neurons.

The relevant Results text, Methods description, and figure legend have been revised accordingly (Results, line 239-243; Methods, lines 662–663).

10). The justification in the text for focusing on somatosensory in Figure 4B,C is not accompanied by any data and the representative images are not convincing.

Response: The rationale for focusing on the primary somatosensory cortex (S1) has been strengthened with direct comparative regional data. In the revised manuscript, we replaced the original representative images and focused the analysis on quantitative Golgi–Cox reconstruction, Sholl analysis, and total dendritic length measurements across S1, hippocampus, and striatum. These analyses showed significant dendritic abnormalities in S1 neurons, including both layer 2/3 and layer 5 neurons, whereas comparable alterations were not detected in hippocampal or striatal neurons (Fig. 3H–K; lines 246-253). Thus, the revised focus on S1 is now supported by comparative regional quantification rather than representative images alone.

We also added P12 RNAscope analysis for *Numb*, a representative NDM-enriched transcript, combined with IBA-1 immunostaining. *Numb*$^{+}$ IBA-1^{+} microglia were detectable across S1, hippocampus, and striatum, without significant regional enrichment in S1 (Fig. 4C, D; lines 258-264). We have therefore revised the

interpretation to avoid implying that the S1 phenotype is driven by selective regional accumulation of Numb-expressing microglia. Instead, the data suggest that S1 may be particularly sensitive to early postnatal microglial perturbation during this developmental window.

11).Open area time feels more appropriate than open distance for EZM metric. Also the figure would be easier to read if the EZM graphs accompanied the EZM heatmaps.

Response: The elevated zero-maze analysis and figure layout have been revised accordingly. We replaced open-arm distance with open-arm time as the primary open-arm readout in both the PLX5622 and LPS experiments, given the potential influence of locomotor activity on distance-based measurements. We also repositioned the elevated zero-maze quantification panels next to the corresponding heatmaps to improve readability and facilitate direct comparison between representative behavioral traces and quantitative results (Fig. 4E, F and Fig. 5B).

12). Micorglia in UMAP 5E: Need to make it more clear in Fig 5E that this includes the initial saline data from Figure 1 AND the "new" LPS data. The BAM data (Fig 5E-F) could be moved to supplement, as it distracts from the focus of the manuscript

Response: We have revised the labels and figure legend for the P12 UMAP analysis to make clear that this analysis includes both the NS/control cells from the baseline dataset and the LPS-treated cells generated for the comparative analysis. The overview UMAP is now labelled accordingly, clarifying that NS/control and LPS-treated cells were analyzed within the same comparative framework.

To improve figure focus, we moved the broader $CD45^+$ immune-cell overview, including the joint UMAP and composition plots of major immune-cell compartments, to Fig. S5C, D, with supporting marker analyses shown in Fig. S5E–G. More detailed BAM-focused analyses are now presented in Figs. S9 and S10.

In the main P12 analysis, we retained only the BAM-related information necessary to distinguish parenchymal microglial states from BAM/Inf_BAM populations. This distinction supports the interpretation that the NDM-associated program is linked to parenchymal microglial states rather than reflecting a BAM-associated macrophage signature. These revisions allow the main figures to focus more directly on the microglial states central to the developmental–inflammatory framework, including NDM, Mg1, and RPIM.

13). Are APIMs invading macs that become microglia?

Response: We do not interpret APIM1/2 as invading macrophages that become microglia. In our dataset, APIM1/2 retained microglial identity-associated genes, particularly *Hexb*. This is relevant because *Hexb* has been identified as a stably expressed microglial core gene that remains informative even under pathological conditions, when several conventional microglial markers may be reduced[1].

Consistent with this microglial identity, APIM1/2 were clearly separated from Pf4^{+} BAM-related populations in the UMAP space. In contrast, canonical macrophage/BAM-associated genes, including *Pf4, Mrc*1, and *Ms4a7*, were enriched in BAM and Inf_BAM populations rather than in APIM1/2. APIM1/2 also did not express representative peripheral immune-cell markers, such as *Cd79a, Cd3e, Ly6c2,* or *Ngp.*

Based on these transcriptional features, we annotate APIM1/2 as LPS-induced inflammation-remodeled microglial states rather than macrophages or BAM-derived cells. We have added this clarification to the revised Results, stating that APIM1 and APIM2 retained *Hexb* expression and remained separated from *Pf4*$^{+}$ BAM-related populations, supporting their microglial annotation(lines 345-348 and lines 405-408; Fig. S5C–G and Fig6H,J).

Ref:

[1]. Masuda, Takahiro et al. "Novel Hexb-based tools for studying microglia in the CNS." *Nature immunology* vol. 21,7 (2020): 802-815.

14).Need to clarify in the figure that the UMAP in 5G is just the LPS mice

Response: The corresponding UMAP panel, now shown as Fig. 6A in the revised manuscript, has been labelled as "UMAP of LPS microglia" to make clear that this analysis includes only microglia from LPS-treated mice.

15). Histology in 5K is nice but it is not entirely related to what the rest of this figure is trying to show. This figure just feels unnecessary .

Response: The histological panels from the original Fig. 5K, L have been moved to Fig. S4A–C to improve the focus and readability of the main figure. These panels are now retained as supporting in situ evidence for LPS-associated changes in IBA-1^{+} and P2RY12^{+} microglial features across P3–P12, while the main figure focuses on the transcriptional remodeling of LPS-treated microglia.

16).What is meant by "RPIM underwent striking reprogramming" (Line 333)? Compared to the RPIM clusters in figure 5? How can that comparison be made?

Response: We have removed the phrase "RPIM underwent striking reprogramming" because it could imply a direct temporal or lineage-based comparison that was not tested in that analysis. In the revised Results, we now describe the comparison more precisely as a state-level transcriptional comparison within the integrated P12 NS/LPS dataset.

Specifically, RPIM is now described relative to physiological microglial states, particularly NDM and Mg1, within the same P12 dataset. RPIM was enriched for inflammatory, immunometabolic, and phagolysosomal genes, whereas NDM showed stronger developmental and metabolic features **(**Fig. 5G–I; lines 305-306**)**. This revised wording avoids implying a direct transition from another RPIM cluster or a lineage relationship.

17). Histology corroborating the findings in Figure 6I would be nice.

Response: We added P12 RNAscope analysis comparing NS and LPS brains to provide in situ corroboration for the transcriptional reduction of NDM-associated genes.

Specifically, we examined *Numb*, a representative NDM-enriched transcript associated with the neurodevelopment-related program, together with IBA-1 immunostaining to identify microglia. We then quantified the proportion of *Numb*$^+$ IBA-1$^+$ microglia among total IBA-1$^+$ cells in tissue sections. This analysis showed a significant reduction in *Numb*$^+$ IBA-1$^+$ microglia after neonatal LPS exposure compared with NS controls (NS, 50.6 ± 3.6%; LPS, 19.7 ± 1.6%; revised Fig. 5L, M).

These data provide independent in situ support for the reduction of NDM-associated molecular features after neonatal LPS exposure, consistent with the transcriptional findings shown in Fig. 6I. The new RNAscope data have been incorporated into the revised Results (lines 329–335) and the corresponding figure legends.

18). Need indication that this is now P3 cells only for the UMAP clustering in Fig7A

Response: The corresponding UMAP panel, now shown as Fig. 6F in the revised manuscript, has been labelled to clearly indicate that the clustering analysis was performed using P3 cells only. The revised label and figure legend also specify that this P3 dataset includes both microglial and BAM populations.

19). highlight to bring APIM1 and APIM2 to attention in 7G. That heatmap is terribly large and mostly irrelevant other than those two groups.

Response: The corresponding heatmap, now shown as Fig. 6L, has been simplified to focus on the relevant P3 populations and to make APIM1 and APIM2 more visually

prominent. We reduced the complexity of the panel by retaining the metabolic flux modules most relevant to the APIM1/APIM2 comparison. These revisions improve readability and allow the panel to more directly support the conclusion that APIM2 exhibits a distinct metabolic profile during the acute inflammatory response.

20). 7H-K belongs in the supplement

Response: We have moved the original Fig. 7H–K to Fig. S8 to simplify the main figure and improve readability.

21). 8D is not showing what is stated in the text

Response: The original panel has been replaced, and the corresponding Results text and figure legend have been revised so that the displayed analysis and manuscript description are now aligned.

In the revised manuscript, this analysis is now shown as Fig. 7D, E. The revised figure displays pseudotime-associated dynamics of representative metabolic, stress/inflammatory, and homeostasis-associated genes, including *Pgam1*, *Pgk1*, *Saa3*, *Sod2*, *Aif1*, and *P2ry12*. The corresponding Results text has been revised to describe these gene dynamics explicitly (lines 444-449), and the figure legend has been updated accordingly.

22). 8F needs to be labelled as APIM2 cells

Response: The corresponding panel, now shown as Fig. 7H in the revised manuscript, has been labelled as APIM2 cells, with the APIM2 substates indicated. The figure legend has also been updated accordingly.

23). Why hippocampus in 8G-H and not somatosensory cortex?

Response: The original hippocampal analysis in Fig. 8G, H has been replaced with analyses focused on the primary somatosensory cortex (S1), which is more closely aligned with the regional phenotype observed after early postnatal microglial perturbation.

In the revised manuscript, the corresponding panels are now shown in Fig. 7F, G and include both whole-brain-level and S1-specific quantification. At the whole-brain level, neonatal LPS exposure increased the proportion of IBA-1^+ cells positive for PGK1 and PGAM1. In S1, the proportion of PGK1^+ IBA-1^+ cells was significantly increased after neonatal LPS exposure, whereas PGAM1^+ IBA-1^+ cells showed an upward trend that did not reach statistical significance ($P = 0.06$). The Results text and figure legend have

been revised to reflect this distinction and to avoid overstating the S1 PGAM1 result.

These revisions have been incorporated into the revised Results section (lines 449-452) and the corresponding figure legend.

Reviewer #3 (Remarks to the Author):

In this manuscript by Zhu, Xu et al., the authors aim to unravel the heterogeneity and functional relevance of microglia, the brain resident macrophages, during postnatal development and in the context of neonatal inflammation. They present a comprehensive single-cell atlas of postnatal microglial states in mice at P3, P7, and P12. Using scRNA-seq, pseudotime reconstruction, and functional perturbations (including PLX5622-mediated microglial depletion and neonatal LPS exposure), they identify a novel microglial state that peaks during the second postnatal week, termed NDM. This population is proposed to support cortical dendritic maturation and social behavior. The study further shows that neonatal LPS exposure leads to a reorganization of microglial states, marked by the expansion of metabolically primed inflammatory microglia (APIM2) and incomplete recovery of homeostatic or neurosupportive programs.

Overall, this work identifies an interesting and potentially important transient developmental microglial state with clear relevance for understanding postnatal brain maturation. The authors thus address a major question and provide promising insights into microglial heterogeneity during development. However, several aspects of the study are incomplete, and the different sections are not well integrated. The NDM state is described almost exclusively at the transcriptomic level, with limited in situ validation or spatial characterization. The two main parts of the manuscript (the description of NDM and the analysis of neonatal inflammation) are not well connected making it difficult to assess functional connections between NDM identity, cortical circuit maturation, and the effects of neonatal inflammation.

(1). NDM state characterization

Reviewer comment 1. *The identification of a new microglial state (NDM) enriched during the second postnatal week is an important finding. However, the spatial and temporal characterization of this state remains incomplete. It is unclear whether NDM microglia are restricted to the somatosensory cortex or broadly distributed across brain regions. The authors should provide both qualitative data from immunolabeling as well as quantification showing the proportion of microglia expressing NDM markers*

(QK, NAV2, HK2) in relevant regions at different time points.

Response: We appreciate the reviewer's important point regarding the spatial and temporal characterization of NDM-associated microglia. In the revised manuscript, we added qualitative and quantitative in situ validation using RNAscope combined with IBA-1 immunostaining.

Specifically, we performed RNAscope in situ hybridization for *Numb*, a representative NDM-enriched transcript associated with the neurodevelopment-related gene program, together with IBA-1 immunostaining to identify microglia. Although the reviewer suggested *Qk*, *Nav2*, and *Hk2* as candidate NDM markers, we selected *Numb* for the regional and temporal analysis because, among the NDM-enriched candidates evaluated under our experimental conditions, it provided robust, reproducible, and readily quantifiable in situ signals. We therefore used *Numb* as an orthogonal in situ readout of NDM-associated molecular features.

Using this approach, we examined the temporal dynamics of *Numb*$^+$ IBA-1$^+$ microglia across P3, P7, P12, and P17. The proportion of *Numb*$^+$ IBA-1$^+$ microglia was low at P3 and P7 (6.3 ± 0.4% and 9.5 ± 1.3%, respectively), increased markedly at P12 (50.6 ± 3.6%), and declined by P17 (15.6 ± 0.9%; Fig. 2G,H). We next assessed the regional distribution of *Numb*$^+$ IBA-1$^+$ microglia at P12 in the primary somatosensory cortex (S1), hippocampus, and striatum, representing cortical, hippocampal, and subcortical regions. *Numb*$^+$ IBA-1$^+$ microglia were detected across all three examined regions, with no significant enrichment in S1 relative to the hippocampus or striatum (Fig. 4C, D). Thus, within the regions examined, microglia expressing this NDM-associated transcript were broadly distributed and were not restricted to the somatosensory cortex.

These regional data helped refine our interpretation of the S1 phenotype after early postnatal microglial depletion. Because *Numb*$^+$ IBA-1$^+$ microglia were detected across S1, hippocampus, and striatum without significant enrichment in S1, the preferential structural alterations in S1 are unlikely to be explained simply by higher local abundance of microglia expressing this NDM-associated transcript. Instead, these findings suggest that S1 circuits may be particularly sensitive to microglial perturbation during this early postnatal developmental window, potentially reflecting the active dendritic and synaptic maturation of somatosensory circuits.

The new RNAscope images, quantifications, and corresponding Methods details have

been incorporated into the revised Results (lines 206-212 and 258-264), Discussion (lines 497-519), and Methods (lines 590-608).

Reviewer comment 2. *The authors state that NDM is a transient state, but no data are provided to demonstrate its disappearance at later time points. Including later developmental stages would strengthen this point. Additionally, the acronym NDM may be confusing since it refers to a late developmental window, a clearer designation could help avoid ambiguity in the field (especially with the designation of PEM).*

Response: We thank the reviewer for this important and constructive comment. This point led us to strengthen the temporal evidence for NDM and to refine how this state is described and named in the revised manuscript. We have added post-P12 experimental validation, incorporated later-stage developmental reference datasets, and revised the terminology to avoid implying that NDM is defined solely by a transient time window or by a terminal maturation stage.

Our original P3–P12 scRNA-seq dataset defined the emergence and peak enrichment of NDM during early postnatal development, but did not by itself determine how this state evolves after P12. We have therefore revised the manuscript to avoid using “transient” or “second-week-dominant” as stand-alone conclusions based solely on the original scRNA-seq time course. Instead, we now describe NDM more precisely as a developmentally restricted postnatal neurodevelopment-associated microglial state.

First, we extended the experimental validation beyond P12 by performing RNAscope in situ hybridization for *Numb*, a representative NDM-enriched transcript associated with the neurodevelopment-related program, combined with IBA-1 immunostaining across P3, P7, P12, and P17. This analysis showed that the proportion of *Numb*$^+$ IBA-1$^+$ microglia was low at P3 and P7 (6.3 ± 0.4% and 9.5 ± 1.3%, respectively), increased markedly at P12 (50.6 ± 3.6%), and declined by P17 (15.6 ± 0.9%; Fig. 2G.H). Although Numb alone does not define the full NDM transcriptional state, these new in situ data provide post-P12 experimental evidence that an NDM-associated program is enriched around P12 and substantially reduced by P17.

Second, we extended the developmental analysis to P30 by integrating our dataset with the open-access developmental microglial atlas from Hammond et al.[1], which includes E14.5, P4/P5, and P30 microglia. We selected this dataset for formal integration because it was generated using a droplet-based single-cell platform and is therefore

technically more comparable to our droplet-based SeekOne dataset than plate-based datasets. In this cross-dataset analysis, cells mapped to an NDM-like identity were concentrated within the postnatal developmental compartment, were minimally represented at E14.5, and were only sparsely detected at P30. In contrast, P30 microglia mapped predominantly to Mg1-like or homeostatic states (Fig. 2I,J and Fig. S2F, G). This later-stage analysis supports the interpretation that the NDM-associated program is not maintained as a major microglial identity by P30.

Third, we reviewed additional published developmental microglial scRNA-seq atlases that include P21 and later stages [1–3]. To present this supporting evidence transparently, we summarized the reported later-stage microglial populations from representative datasets in Response Table 1 and Response Fig. 1. Across these studies, microglia at P21, P30, P60, and adult stages are mainly assigned to homeostatic, maturation-associated, or adult-like clusters. Importantly, the reported annotations and marker profiles do not support the presence of NDM as a dominant neurodevelopment-associated microglial identity at P21 or later stages. Because these datasets differ in platform, sampling design, and cluster nomenclature, we use them as supportive later-stage reference evidence rather than as direct proof of complete NDM absence. Nevertheless, they provide independent contextual support that NDM is not sustained as a dominant microglial identity during later postnatal maturation.

Together, our P3–P12 scRNA-seq data, newly added P17 RNAscope validation of *Numb*, P30 cross-dataset integration, and published developmental atlases covering P21 and later stages provide convergent evidence that NDM is enriched within a restricted postnatal developmental window, peaks around P12, and declines thereafter. These findings support the refined conclusion that NDM represents a developmentally restricted postnatal neurodevelopment-associated microglial state, rather than a mature or adult-like microglial identity sustained into later postnatal or adult stages.

We also carefully considered the reviewer's concern regarding the acronym NDM. In the revised manuscript, we clarify at first mention that NDM denotes neurodevelopment-associated microglia, referring to the transcriptional and functional features enriched in this state, rather than to a fixed late-developmental stage or terminal maturation identity. We retained the acronym NDM because it captures the defining biological program of this population—its enrichment for neurodevelopment-related and metabolism-associated features—while avoiding a

purely time-based name. To reduce ambiguity, we no longer use "second-week-dominant" as a stand-alone descriptor and instead refer to NDM as developmentally restricted within the early postnatal period. We have also clarified the distinction between NDM and PEM: PEM denotes an earlier postnatal microglial state with lysosomal, lipid-handling, and oxidative-stress-associated features, whereas NDM denotes a later-enriched neurodevelopment-associated program. This revised terminology should help avoid confusion between temporally adjacent but transcriptionally distinct postnatal microglial states.

These revisions have been incorporated into the Results section 2.3 (lines 206-220), the Discussion (lines 497–506 and 536-541), and the Methods (lines 590–608 and 752–762) of the revised manuscript.

Reviewer comment 3*. While five co-expression modules are identified, the manuscript does not discuss the regulatory mechanisms or signaling pathways that might drive the Mg1 vs. NDM bifurcation. Highlighting candidate transcription factors or upstream regulators would provide mechanistic insight.*

Response： We thank the reviewer for this constructive comment. The original manuscript identified co-expression modules associated with the Mg1–NDM divergence, and we have now extended this analysis to nominate candidate regulatory programs that may contribute to this bifurcation.

To address this point, we added transcription factor activity inference using aggregated single-cell expression profiles across the P3–P12 postnatal developmental time course. This analysis was designed to identify candidate upstream regulatory differences associated with the divergence between the Mg1-enriched homeostatic maturation branch and the NDM branch.

The revised analysis identified distinct transcription factor activity patterns associated with Mg1 and NDM. *Maml1*, *Hif3a*, *Zmynd8*, and *Etv3* showed higher inferred activity in NDM, whereas *Jun*, *Hif1a*, *Sp1*, and *Tbpl1* showed higher inferred activity in Mg1. Among these candidates, *Maml1* showed the most consistent higher inferred activity in NDM across developmental time points. The broader state-level analysis further showed that NDM and Mg1 occupied distinct transcription factor activity profiles, supporting regulatory divergence between the NDM branch and the Mg1-enriched homeostatic maturation branch.

These analyses have been incorporated into the revised Results (lines 192-196) and are shown in revised Fig. 2E and Fig. S2A–C. The candidate regulators identified here provide a focused basis for future functional studies testing the mechanisms that drive NDM specification and Mg1 maturation.

Reviewer comment 4. *Finally, the authors should show in the scRNA-seq data whether NDM-associated genes are microglia and not shared with border-associated macrophages (BAMs)*.

Response: We appreciate the reviewer's point. In the revised manuscript, we explicitly compared representative NDM-associated genes with canonical BAM-associated markers in the integrated P12 scRNA-seq dataset to distinguish the NDM program from BAM-related macrophage signatures.

This analysis showed that NDM-associated genes, including *Hk2*, *Numb*, and *Txnip*, were preferentially expressed in the NDM population and were not broadly expressed by BAM or Inf_BAM populations. Conversely, BAM and Inf_BAM preferentially expressed canonical BAM-associated genes, including *Pf4*, *F13a1*, and *Ms4a7*.

These results support the interpretation that the NDM-associated program reflects a parenchymal microglial state rather than a BAM-associated macrophage signature. This clarification has been incorporated into the revised Results (lines 294-298) and the corresponding figures (Fig. 5G and Fig. S3A).

(2) Microglia depletion experiments

Reviewer comment 5. *In the PLX5622 depletion experiments, the kinetics of microglial depletion and repopulation are not shown, yet the authors describe outcomes at P21 and P45, when microglia are expected to have repopulated. Still, it remains unknown when NDM cells disappear.*

While we acknowledge that specific ablation of NDM or Mg1 microglia is technically challenging, the authors should describe NDM and Mg1 states before and after PLX treatment. Deciphering whether NDM state is particularly enriched in S1 versus other cortical regions could help to support a causal link between NDM and the dendritic or behavioral outcomes. Including quantitative data from other regions would be important to strengthen the claim that the observed effects are specific of S1.

It would also be important to quantify the repopulation dynamics. It remains unclear whether the reported effects in S1 result solely from the transient absence of

microglia/macrophages or from altered properties of repopulated cells that could fail to re-establish homeostatic microglia or NDM-like state if the NDM state would still be present during/after repopulation.

Furthermore, as PLX5622 target CSF1R-expressing macrophages, the authors should temper their conclusions regarding microglia specific roles.

Response: We thank the reviewer for these important and closely related comments. The PLX5622 experiment was designed as a developmental-window perturbation during the period when NDM and Mg1 normally emerge and become enriched, rather than as a state-specific ablation experiment. In the revised manuscript, we have clarified this point by adding depletion–repopulation kinetics, strengthening the regional analysis of the phenotype, assessing regional distribution of an NDM-associated transcript, and refining the interpretation of CSF1R-dependent myeloid-cell targeting.

First, we quantified depletion and repopulation in the neonatal PLX5622 paradigm. PLX5622 was administered from P5 to P11, a period overlapping the normal emergence and enrichment of NDM and Mg1. IBA-1^{+} cell quantification showed efficient depletion after 3 days of treatment, sustained depletion after 7 days of treatment, and rapid repopulation after PLX5622 withdrawal, with IBA-1^{+} cells largely restored within 72 h (Fig. 3B, C; lines 231-234). These data indicate that the later P21 and P45 phenotypes are unlikely to reflect ongoing gross depletion of IBA-1^{+} cells, but rather are consistent with the consequences of perturbing CSF1R-dependent myeloid cells during this early postnatal developmental window.

Second, we strengthened the regional analysis of the PLX5622 phenotype. Quantitative Golgi–Cox reconstruction, Sholl analysis, and total dendritic length measurements were expanded to S1, hippocampus, and striatum. Early postnatal PLX5622-mediated depletion reduced dendritic complexity and total dendritic length in S1 neurons, including both layer 2/3 and layer 5 neurons, whereas comparable alterations were not detected in hippocampal or striatal neurons (Fig. 3H–K; lines lines 246-253). Thus, the revised manuscript supports the preferential S1 phenotype with direct comparative regional quantification rather than representative images alone.

Third, we examined whether the S1 phenotype could be explained by regional enrichment of microglia expressing an NDM-associated transcript. We added

RNAscope analysis for *Numb*, a representative NDM-enriched transcript associated with the neurodevelopment-related program, combined with IBA-1 immunostaining. At P12, *Numb*$^{+}$ IBA-1^{+} microglia were detected across S1, hippocampus, and striatum, without significant enrichment in S1 (Fig. 4C, D; lines 258-264). These data indicate that the preferential S1 phenotype is unlikely to be explained simply by local enrichment of microglia expressing this NDM-associated transcript.

To support this regional interpretation, we also revised the Discussion to incorporate relevant literature on S1/barrel cortex development. Prior studies have shown that somatosensory circuits undergo active sensory map formation, dendritic and synaptic maturation, and layer-specific refinement during the early postnatal period, particularly around the second postnatal week. Microglia have also been implicated in cortical inhibitory synapse development and whisker-evoked cortical activity during this developmental window[1-4]. Together with our regional quantification, these studies support the interpretation that S1 may be particularly sensitive to early postnatal microglial perturbation, rather than being affected because of selective local enrichment of NDM-associated microglia.

Finally, we refined the state-specific and cell-type-specific scope of the PLX5622 interpretation. PLX5622 targets CSF1R-dependent myeloid cells and does not selectively ablate NDM, Mg1, or any individual microglial state. Moreover, because state-resolved profiling after PLX5622 treatment was not part of the current design, the revised manuscript does not distinguish whether the later phenotypes reflect transient depletion during the developmental window, altered properties of repopulated cells, or both. We therefore frame the PLX5622 data as evidence that cortical maturation is sensitive to CSF1R-dependent myeloid-cell perturbation during the early postnatal window in which NDM and Mg1 normally expand, without attributing the phenotype to NDM or Mg1 alone.

These revisions have been incorporated into the revised Results and Discussion sections, including the depletion–repopulation analysis (Fig. 3B, C; lines 231-234), regional dendritic morphology analysis (Fig. 3H–K; lines lines 246-253), regional RNAscope analysis (Fig. 4C, D; lines 258-264), and the revised Discussion of S1 regional vulnerability and PLX5622-related limitations (lines 512-519 and 541-545).

Ref:

[1]. Pedrosa, Laís Resque Russo et al. “Time Window of the Critical Period for Neuroplasticity in

S1, V1, and A1 Sensory Areas of Small Rodents: A Systematic Review." Frontiers in neuroanatomy vol. 16 763245. 17 Mar. 2022.
[2]. Stern, E A et al. "Rapid development and plasticity of layer 2/3 maps in rat barrel cortex in vivo." Neuron vol. 31,2 (2001): 305-15.
[3]. Gesuita, Lorenzo et al. "Microglia contribute to the postnatal development of cortical somatostatin-positive inhibitory cells and to whisker-evoked cortical activity." Cell reports vol. 40,7 (2022).
[4]. van der Bourg, Alexander et al. "Layer-Specific Refinement of Sensory Coding in Developing Mouse Barrel Cortex." Cerebral cortex vol. 27,10 (2017): 4835-4850.

(3) Neonatal inflammation

Reviewer comment 6*. The LPS-induced neonatal inflammation provide an important link between early life immune challenges and macrophages developmental trajectories, which is highly relevant regarding neurodevelopmental disorders. However, the connection between the first part of the paper (NDM characterization) and the second part (LPS effects) could be strengthen. It would be helpful to analyze whether LPS-induced suppression of NDM microglia leads to similar dendritic defects in S1, as observed following PLX treatment.*

Response: This comment identified an important opportunity to make the connection between the developmental NDM program and the neonatal inflammation arm more explicit. In the revised manuscript, we addressed this issue at two complementary levels: first, by directly validating the effect of neonatal LPS on NDM-associated microglial features in situ; and second, by assessing whether neonatal LPS exposure is accompanied by S1 dendritic abnormalities overlapping with those observed after early postnatal PLX5622-mediated depletion.

To directly link neonatal inflammation to the NDM-associated program, we added P12 RNAscope analysis for *Numb*, a representative NDM-enriched transcript associated with the neurodevelopment-related program, combined with IBA-1 immunostaining. This analysis showed a significant reduction in the proportion of *Numb*$^{+}$ IBA-1^{+} microglia after neonatal LPS exposure compared with NS controls (NS, 50.6 ± 3.6%; LPS, 19.7 ± 1.6%; Fig. 5L, M). These data provide independent in situ evidence that neonatal inflammation reduces NDM-associated molecular features at the developmental stage when NDM is normally prominent.

In parallel, following the reviewer's suggestion, we analyzed dendritic morphology in S1 neurons after neonatal LPS exposure. This analysis showed reduced dendritic complexity and total dendritic length in S1 after LPS exposure (Fig. S5A, B), indicating

that neonatal inflammation is accompanied by S1 structural abnormalities that partially overlap with those observed after early postnatal PLX5622-mediated depletion.

We have framed these findings according to the experimental design. The S1 dendritic analysis establishes phenotypic convergence between neonatal LPS exposure and early postnatal microglial depletion, whereas the P12 Numb RNAscope analysis provides a more direct link between neonatal inflammation and suppression of the NDM-associated microglial program. Because systemic LPS exposure can affect the developing brain through multiple cellular and systemic mechanisms, we do not interpret the LPS-associated dendritic phenotype as evidence that reduced NDM-associated features alone cause the neuronal phenotype. Rather, the revised data support a clearer temporal and phenotypic connection: neonatal inflammation reduces NDM-associated microglial features at P12 and is accompanied by altered S1 structural maturation during the same developmental window.

Together, these additions strengthen the integration of the two major parts of the study while avoiding NDM-specific causal inference from a systemic inflammatory model. Definitive testing of whether loss of NDM-associated microglial features is sufficient or necessary for the LPS-associated S1 phenotype will require future state-specific perturbation or rescue approaches.

These revisions have been incorporated into the revised Results and Supplementary Information, including the P12 *Numb* RNAscope analysis after LPS exposure (Fig. 5L, M) and the S1 dendritic morphology analysis after LPS exposure (Fig. S5A, B).

Reviewer comment 7. *The manuscript refers to "persistent" effects of neonatal inflammation, but the latest analyzed time point is P12. To support persistence, later developmental stages (for instance P21 or P45) should be included to assess long-term transcriptional or morphological changes.*

Response: The reviewer raises an important issue regarding the temporal scope of the term "persistent." In the original manuscript, this wording was intended to indicate that neonatal LPS exposure at P2 induced microglial-state remodeling that remained evident through P12, beyond the acute inflammatory phase. However, to avoid implying long-term transcriptional or microglial-state remodeling beyond the P3–P12 scRNA-seq time course, we have revised this terminology throughout the manuscript.

In particular, we revised two Results subsection titles. The original title "*Neonatal*

inflammation triggers a persistent reorganization of microglial states" has been replaced with "*Neonatal inflammation remodels the P12 microglial landscape and attenuates the NDM program*." This revised wording more accurately reflects the P12 analysis, in which neonatal LPS exposure reduced the relative representation of NDM and Mg1 and expanded RPIM, while attenuating NDM-associated molecular features.

We also replaced the original title "Neonatal inflammation instigates a persistent microglial state shift, depriving the developing cortex of neurodevelopmental support" with "Neonatal inflammation redirects microglia through a sequential APIM-to-RPIM remodeling program." This revised title better reflects the time-resolved analysis across P3–P12, in which neonatal inflammation induced acute post-inflammatory microglial states followed by recovery-phase inflammatory microglial states, without implying long-term persistence beyond the analyzed transcriptomic window.

As an additional check, we examined later-stage histological readouts at P21 using IBA-1 and P2RY12 staining. These analyses did not reveal significant differences between NS and LPS groups in the assessed IBA-1^{+} or P2RY12^{+} microglial measures (Response Fig. 2). We therefore do not extend the conclusion to long-term transcriptional or microglial-state remodeling at P21 or later stages; instead, the P21 data are provided in the response to define the temporal boundary of the LPS-induced microglial changes assessed in this study.

The relevant Results subsection titles have been revised accordingly (lines 275-276 and 336-337).

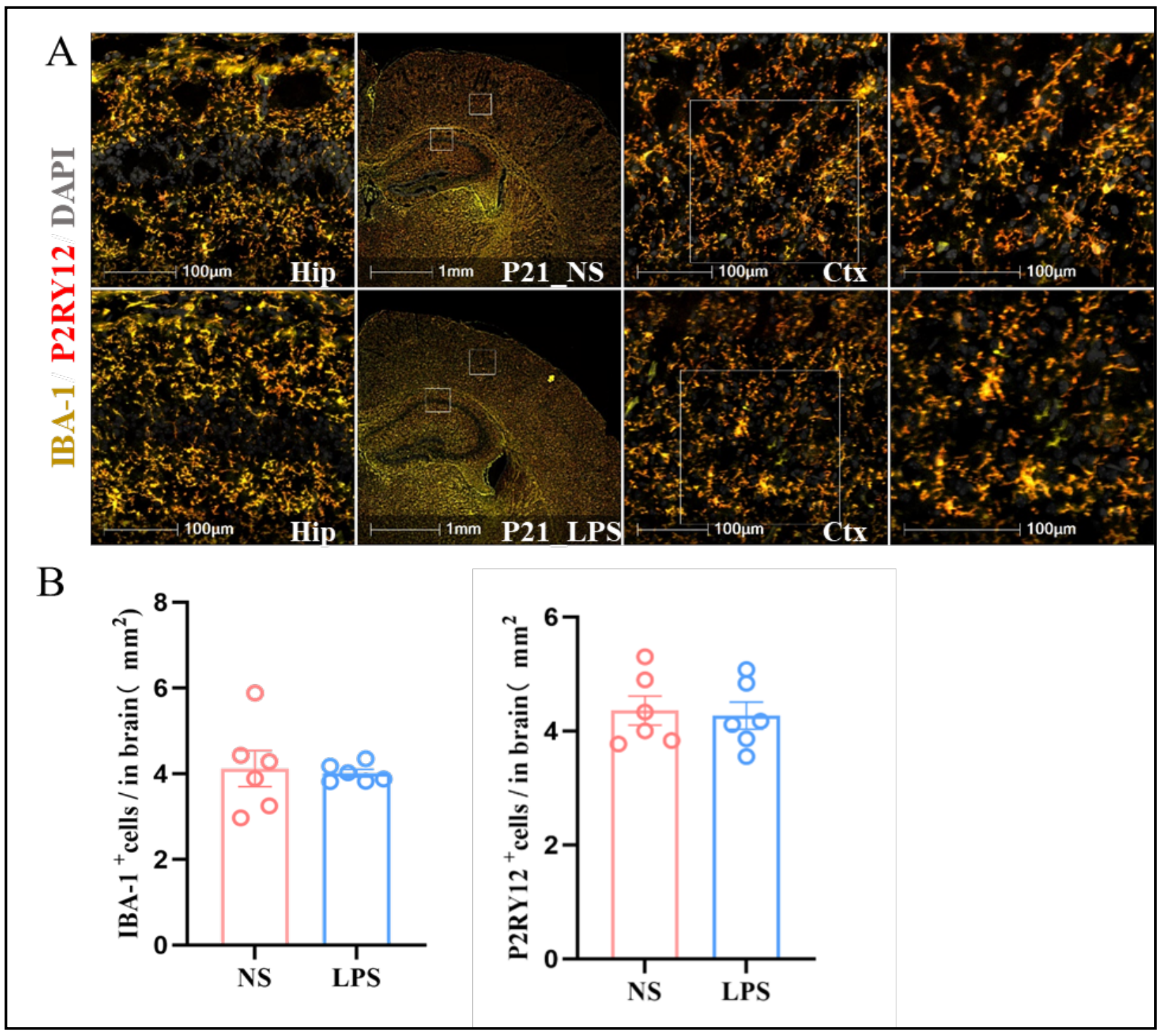

Response Fig.2. P21 IBA-1 and P2RY12 histological assessment after neonatal LPS exposure. A. Representative images of hippocampal and cortical regions from NS- and LPS-treated brains at P21 stained for IBA-1, P2RY12, and DAPI. B. Quantification of IBA-1^+ and P2RY12^+ cell densities in NS- and LPS-treated brains at P21. No significant differences were detected between groups for the assessed markers. Data are shown as mean ± SEM; n = 6 per group.

Reviewer comment 8*. To support the proposed model that early metabolic reprogramming (P3) prevents NDM recovery by P12, microglia-specific manipulation of HIF1α or LDHA (via knockout or pharmacological inhibition) could test whether reducing Warburg-like metabolism restores neurodevelopmental transcriptional programs.The authors should consider discussing whether the observed metabolic changes are intrinsic to microglia or are potentially influenced by BAMs.*

Response: The reviewer highlights an important mechanistic extension of our study. Direct manipulation of HIF1α, LDHA, or related glycolytic regulators would be a powerful way to test whether early Warburg-like metabolic remodeling causally constrains the later establishment of the NDM-associated developmental program after neonatal inflammation.

We have clarified in the revised manuscript that this causal question requires a dedicated experimental framework distinct from the scope of the present study. Such experiments would need temporally controlled and microglia-directed metabolic perturbation after neonatal inflammatory exposure, followed by state-resolved assessment of microglial recovery and neurodevelopmental outcomes. This would constitute a separate mechanistic study rather than a direct extension of the current single-cell atlas and validation framework. Accordingly, we now present early metabolic remodeling as a candidate upstream mechanism associated with later impairment of the NDM-associated program, rather than as a proven causal driver.

In the current study, the data support a temporal and transcriptional link: an APIM2-associated glycolytic and ATP-metabolic program emerges within 24 h after neonatal LPS exposure and precedes the impaired establishment of the NDM-associated developmental program observed at P12. We have revised the Discussion to state explicitly that testing causality will require microglia-directed perturbation of candidate regulators such as HIF1α and LDHA (lines 545-549).

We have also expanded the Discussion to address whether the metabolic changes should be interpreted as exclusively intrinsic to parenchymal microglia. The revised text now notes that related metabolic–inflammatory coupling was also observed in BAM-related populations, indicating that acute immunometabolic remodeling after neonatal LPS exposure may involve multiple brain-resident macrophage compartments. However, the developmental interpretation differs by compartment. BAMs undergo inflammatory remodeling but do not normally establish an NDM-like neurodevelopmental program under physiological conditions. In contrast, parenchymal microglia are the compartment in which the NDM-associated developmental program normally emerges and is attenuated after neonatal inflammation.

Thus, we now frame early metabolic remodeling as a broader brain-resident macrophage response to neonatal inflammation, while linking the impaired establishment of the NDM-associated developmental program specifically to the parenchymal microglial compartment. These clarifications have been incorporated into the revised Discussion (lines 528-530 and 545-549).

Reviewer comment 9. *The bifurcation between the "chemokine/NF-κB" and "interferon/antiviral" APIM2 trajectories is transcriptionally defined, but the proportion of cells following each trajectory over time (P3–P12) is not quantified. Linking these trajectories to anatomical regions would strengthen the interpretation.*

Response: This comment helped us identify a point where the original wording could imply a stronger temporal or lineage interpretation than was intended. In the revised manuscript, we have clarified that the APIM2 analysis refers to transcriptional heterogeneity within the acute P3 response, rather than to two lineage-resolved trajectories followed from P3 to P12.

Specifically, the APIM2 subanalysis was performed on P3 LPS-associated microglia, where high-resolution subclustering and pseudotime ordering revealed a graded transcriptional continuum within APIM2. This continuum extended from cells with stronger metabolic and biosynthetic features toward cells with higher inflammatory, chemokine, and interferon/antiviral programs. We have therefore revised the text to describe APIM2 as an acute P3 metabolically activated state that diversifies along inflammatory axes, rather than as a set of quantified P3–P12 trajectories (lines 439-444).

We did not assign P7 or P12 cells to discrete APIM2-derived chemokine/NF-κB or interferon/antiviral branches, because doing so would require assumptions about state continuity that are not directly resolved by the current cross-sectional dataset. At later time points, the LPS-remodeled microglial compartment is dominated by RPIM states rather than a preserved APIM2 substructure. Accordingly, the revised manuscript avoids implying that the P3 APIM2 continuum represents stable branches that can be quantitatively tracked across P3–P12.

We also clarified the spatial limitation of this analysis. The scRNA-seq data were generated from dissociated whole-brain CD45$^+$ cells and therefore do not retain anatomical region-of-origin information. Thus, the current dataset cannot determine whether the APIM2 inflammatory programs are regionally patterned. Resolving this question would require a spatially resolved design, such as regional sorting followed by scRNA-seq, multiplexed in situ hybridization, or spatial transcriptomics.

Thus, in the revised manuscript, we present the APIM2 analysis as evidence of acute transcriptional diversification within P3 inflammation-remodeled microglia, not as a quantified P3–P12 lineage trajectory or anatomically resolved branch structure.

Reviewer comment 10*. The emergence of Inf_BAM cells is intriguing, but their potential role in cortical inflammation remains unclear. Anatomical localization (meningeal vs. perivascular) using Mrc1/CD206 immunostaining would help clarify their potential influence on cortical microglia.*

Response: The reviewer raises an important point regarding the anatomical context of Inf_BAM cells and their potential relationship to cortical inflammation. To explore this issue, we performed CD206 immunostaining at P3 in NS- and LPS-treated brains. This exploratory analysis detected CD206$^+$ cells predominantly along the brain surface/meningeal region in both conditions, without an obvious qualitative redistribution after neonatal LPS exposure (Response Fig. 3). These images are provided in the response letter as tissue-level assessment of CD206$^+$ BAM-related cells after neonatal inflammation.

We interpret these data with appropriate caution. CD206/MRC1 immunostaining alone cannot rigorously distinguish meningeal BAMs from perivascular BAMs, nor can it assign the scRNA-seq-defined Inf_BAM state to a specific anatomical niche. Definitive spatial localization would require quantitative region-resolved analysis with

additional compartment markers and higher-resolution anatomical annotation.

Accordingly, we do not use the CD206 staining to infer that Inf_BAM cells occupy a specific meningeal or perivascular compartment, or to claim a direct functional influence of Inf_BAM cells on cortical microglia. In the revised manuscript, Inf_BAM is described as an inflammation-associated BAM-like transcriptional state identified by scRNA-seq. Consistent with the central focus of the study, BAM-focused analyses have been moved from the main Results to the Supplementary Information, where they serve as contextual comparison data rather than a primary mechanistic axis.

Future spatially resolved analyses, such as multiplexed immunostaining or spatial transcriptomics incorporating additional BAM compartment markers, will be required to define the anatomical localization and functional contribution of Inf_BAM cells after neonatal inflammation.

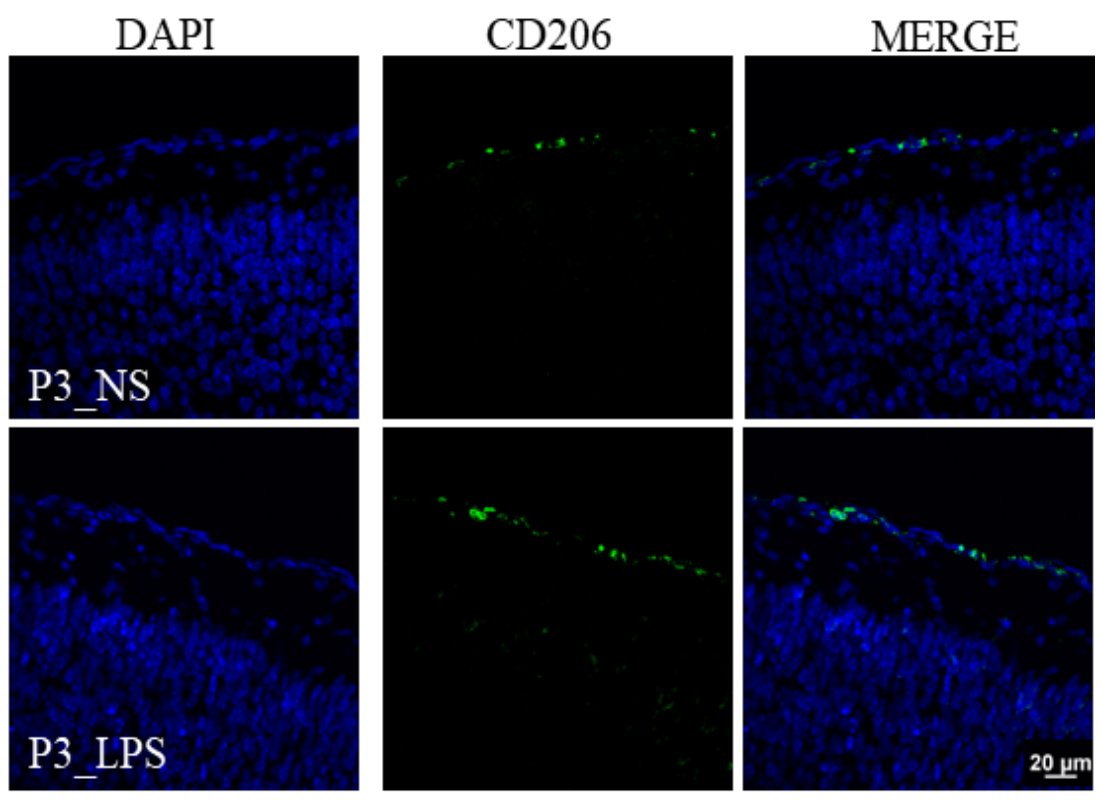

Response Fig. 3. Exploratory CD206 immunostaining after neonatal LPS exposure. Representative cortical sections from NS- and LPS-treated brains at P3 stained for CD206 and DAPI. $CD206^{+}$ cells were detected predominantly along the brain surface/meningeal region in both conditions. This qualitative assessment did not reveal an obvious redistribution after neonatal LPS exposure. Scale bar, 20 μm.

(4) Additional points

Reviewer comment 11*. The number of biological replicates per condition and per time point should be clearly stated.*

Response: The relevant figure legends and Methods have been updated to clearly state the number of biological replicates for each condition and time point where applicable. We have also clarified the corresponding animal numbers and independent litter information in the revised manuscript to improve transparency and

reproducibility of the experimental design.

Reviewer comment 12. *Given that neurodevelopmental disorders linked to early life inflammation often show sex-specific outcomes, the authors should clearly indicate the sex of animals used in each experiment, especially in the PLX and LPS models, and specify whether any sex-dependent differences were observed.*

Response: We thank the reviewer for raising this important point. Sex-specific effects are highly relevant to neurodevelopmental outcomes after early-life inflammation, and we agree that the sex of animals used in the PLX5622 and LPS experiments should be clearly reported. In the revised manuscript, we have updated the relevant statistical plots to indicate the sex of each individual animal directly in the data displays for the PLX5622 and LPS experiments, including Figs. 2–7 and the corresponding supplementary figures. We have also clarified the sex composition of the experimental cohorts in the figure legends and Methods.

With individual data points now annotated by sex, we did not observe an obvious sex-dependent separation across the measured outcomes in these experiments. However, the study was not designed or statistically powered to conduct formal sex-stratified analyses. We therefore do not make sex-specific claims or infer sex-dependent effects from these datasets. The revised presentation allows readers to assess the distribution of male and female animals directly, while maintaining an interpretation consistent with the experimental design.

We have explicitly stated this limitation in the revised Discussion (lines 549-554) and updated the relevant figure legends and Methods accordingly.

Reviewer comment 13*. The authors should include representative images of microglia from both control and LPS conditions, indicating brain regions (Figure 5K).*

Response: Representative images of IBA-1 and P2RY12 staining from both NS/control and LPS-treated brains have been added, with the corresponding brain regions clearly indicated. To maintain the focus and readability of the main figures, these histological images and quantifications are now presented in Fig. S4A–C.

These panels provide orthogonal tissue-level support for LPS-associated changes in IBA-1^{+} and P2RY12^{+} microglial features across P3–P12, while the main figures remain focused on scRNA-seq-defined microglial state remodeling after neonatal inflammation.

Reviewer comment 14. *Some figure citations appear missing throughout the text and should be added to guide readers through the data.*

Response: The revised manuscript has been systematically reviewed, and missing figure citations have been added throughout the main text where appropriate. These revisions ensure that each result is clearly linked to the corresponding data panel and improve the readability of the manuscript.

Reviewer #4 (Remarks to the Author):
I co-reviewed this manuscript with one of the reviewers who provided the listed reports. This is part of the Nature Communications initiative to facilitate training in peer review and to provide appropriate recognition for Early Career Researchers who co-review manuscripts.

Point-by-point Response to the Reviewers' Comments

Reviewer #3 (Remarks to the Author):

The authors have done a good job addressing the reviewers comments. Overall, I think they have responded well to the concerns raised. The manuscript has been strengthened by the addition of new experimental data, and several conclusions have been revised. In particular, the spatiotemporal dynamics is now clearer and more convincing.

My remaining concern relates to the nomenclature. In my view, the term "neurodevelopment-associated microglia" remains too broad, as many microglial populations present during development could reasonably be considered neurodevelopment-associated. The proposed acronym therefore does not sufficiently capture the specific features that distinguish this population from other developmental microglial states described in the literature. Given the already large number of acronyms used to define developmental and context-specific microglial populations, a more specific terminology would help avoid confusion and facilitate comparison across studies.

In addition, I found the distinction between the microglial "state" and its molecular signature somewhat unclear throughout the revised manuscript, including in the title. The authors define and discuss a cellular state, but at several points the terminology appears to shift toward describing a molecular or transcriptional program. While these concepts are related, they are not equivalent. A cellular state may be characterized by a particular molecular signature, but the signature itself should not necessarily be interpreted as the state. In this context, I would suggest that the authors consider aligning the terminology more consistently, for example by referring to a microglial state defined by (or associated with) a specific transcriptional/molecular program, rather than using "program". In this context, the previous wording ("state") seemed conceptually more precise and better aligned with the way the results are presented throughout the manuscript.

Apart from these points, I believe the authors have addressed the major concerns raised during the review process, and the manuscript has been substantially improved.

Response: We sincerely thank the reviewer for this thoughtful comment and are pleased that the revisions have strengthened the manuscript. We appreciate the reviewer's suggestions regarding both the nomenclature and the distinction between the cellular state and its associated molecular features, and have revised the manuscript accordingly.

First, we agree that the term "neurodevelopment-associated microglia (NDM)" was overly broad and could be interpreted as encompassing multiple developmental microglial populations described in the literature. To improve specificity and align our

terminology with current recommendations for microglial nomenclature (Paolicelli et al., 2022), we have removed the acronym “NDM” throughout the manuscript and no longer introduce a new acronym. Instead, we now refer to the identified cells as the “postnatal *Numb*-enriched microglial state”, a descriptive, marker-based designation that indicates both a prominent molecular feature and the relevant developmental context, without implying a distinct lineage or fixed functional identity.

Numb was selected because it was consistently enriched in this state in our single-cell transcriptomic analyses and independently validated by spatiotemporal RNAscope, supporting its use as a robust marker across both transcriptomic and tissue-based analyses. The descriptor "postnatal" further distinguishes this state from other embryonic or later developmental microglial states.

Second, we agree that our previous wording did not always clearly distinguish the cellular state from the molecular features. Following the reviewer's suggestion, we now consistently reserve "postnatal Numb-enriched microglial state" to denote the cellular state, whereas "transcriptional signature," "gene module," and "transcriptional program" are used only to describe its associated molecular features. Accordingly, we now describe this state as being characterized by neurodevelopment-related and metabolic transcriptional programs, rather than referring to the program itself as the cellular state. These changes have been applied consistently throughout the title, Abstract, Results, Discussion, and figure legends.

For space-limited figure annotations, we use “$Numb^{+}$Mg” as a graphical label and define it in each corresponding figure legend as representing the postnatal Numb-enriched microglial state. This abbreviated label is used solely for graphical presentation and is not introduced as a formal acronym or independent nomenclature.

Overall, we believe these revisions provide a more specific and transparent nomenclature while maintaining a clear conceptual distinction between the cellular state and its associated transcriptional signatures and programs. The revised terminology is consistent with current recommendations for microglial nomenclature (Paolicelli et al., 2022) and follows the established practice of describing developmental or context-dependent microglial states using characteristic molecular markers

(Wlodarczyk et al., 2017; Hammond et al., 2019; Nomaki et al., 2024). All corresponding revisions have been highlighted in yellow in the revised manuscript.

References：

1. Paolicelli, Rosa C et al. "Microglia states and nomenclature: A field at its crossroads." Neuron vol. 110,21 (2022): 3458-3483.
2. Wlodarczyk, Agnieszka et al. "A novel microglial subset plays a key role in myelinogenesis in developing brain." *The EMBO Journal* vol. 36,22 (2017): 3292-3308.
3. Hammond, Timothy R et al. "Single-Cell RNA Sequencing of Microglia throughout the Mouse Lifespan and in the Injured Brain Reveals Complex Cell-State Changes." *Immunity* vol. 50,1 (2019): 253-271.e6.
4. Nomaki, Kohei et al. "Spatiotemporal dynamics of the CD11c+microglial population in the mouse brain and spinal cord from developmental to adult stages." *Molecular Brain* vol. 17,1 24. 18 May. 2024.
