# Supplementary material for: Neonatal inflammation disrupts a temporally restricted postnatal Numb-enriched microglial state in mice
Source: Nat Commun. 2026 Aug 4;17:9385. doi: 10.1038/s41467-026-76341-6 (PMC13534487; doi:10.1038/s41467-026-76341-6)
Supplement: Supplementary file 3 [file 41467_2026_76341_MOESM1_ESM.pdf]

A

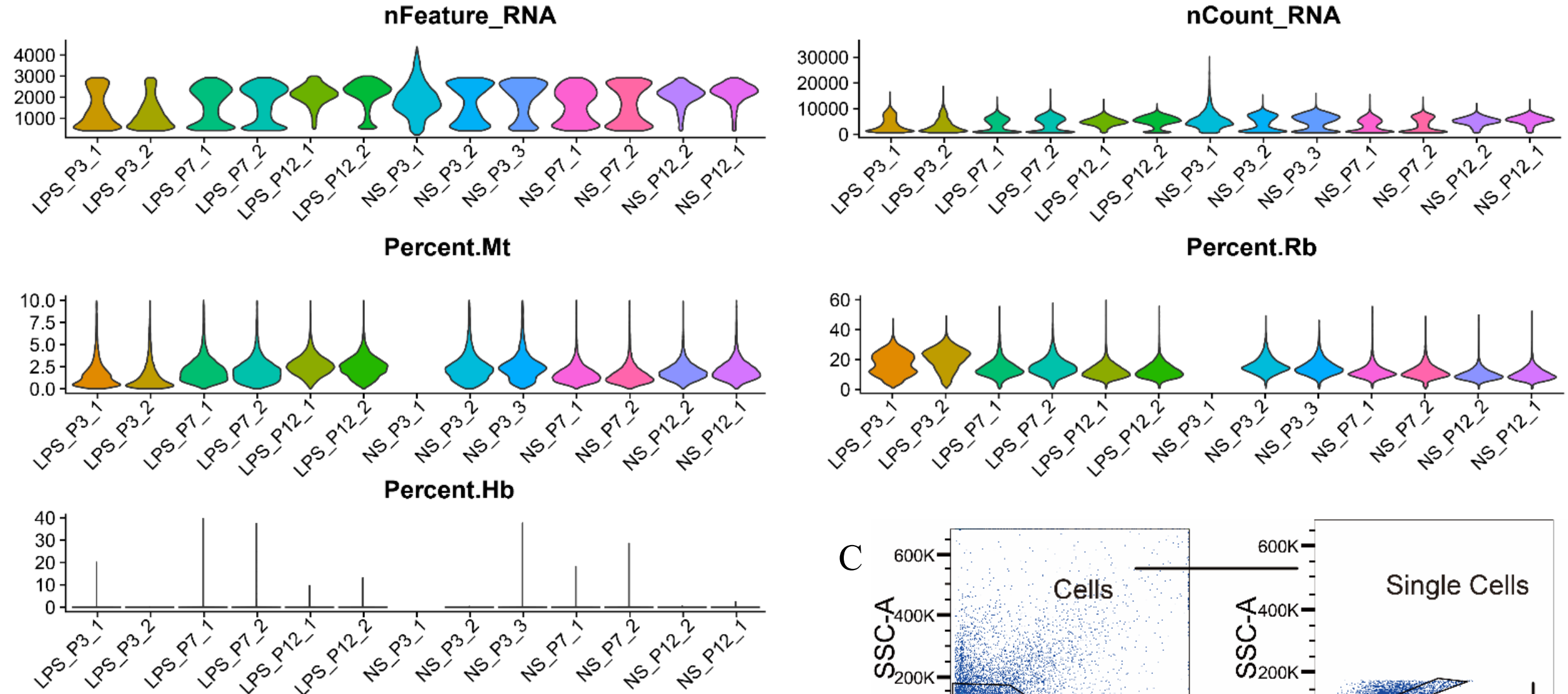

B

Group	nCells	Group	nCells
NS_P3	15570	LPS_P3	**16215**
NS_P7	10975	LPS_P7	**16403**
NS_P12	18441	LPS_P12	**15392**

C

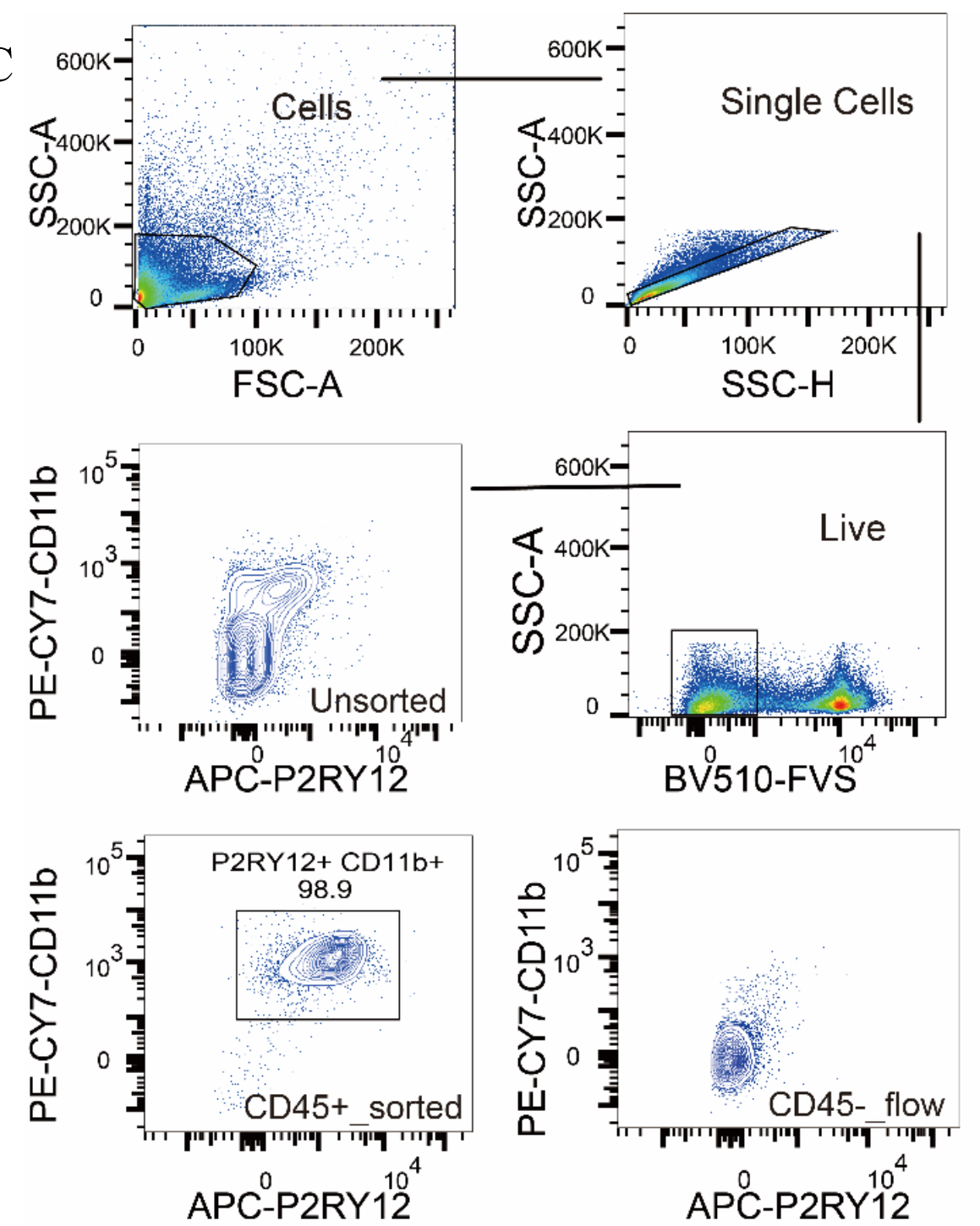

D

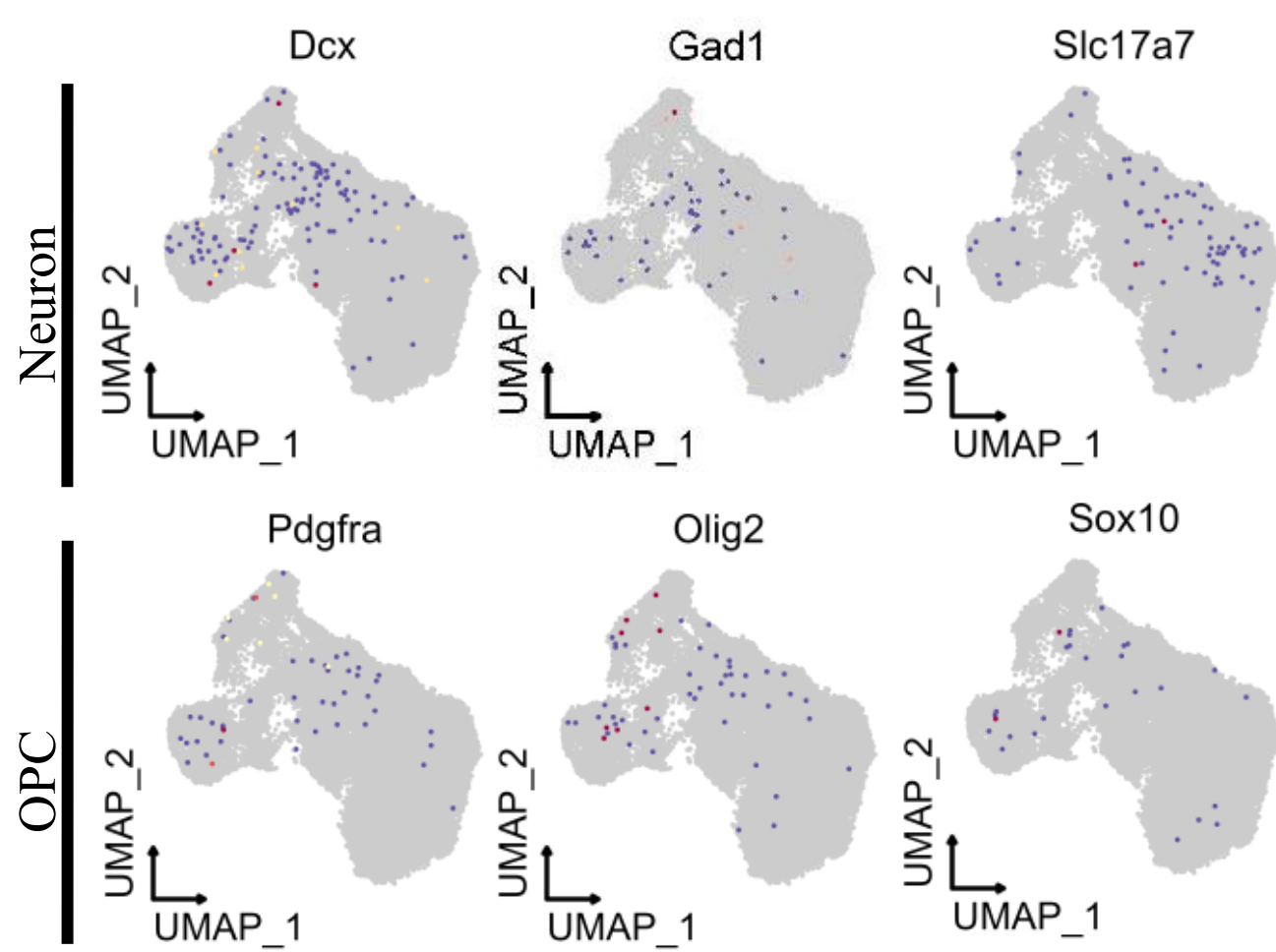

Fig. S1 | Quality control and validation of brain immune cells used for scRNA-seq analysis. **A** Violin plots showing the distributions of quality-control metrics across individual samples, including the number of detected genes per cell (nFeature_RNA), total unique molecular identifier counts per cell (nCount_RNA), and the percentages of mitochondrial (Percent.Mt), ribosomal (Percent.Rb) and haemoglobin transcripts (Percent.Hb). Each violin represents one biologically independent sample derived from one male mouse. **B** Numbers of cells retained in each group after quality-control filtering and included in downstream analyses.
C Representative flow-cytometry gating strategy. Cells were first gated based on forward- and side-scatter properties, followed by singlet and live-cell gating. CD11b and P2RY12 expression was then assessed in unsorted cells, the $CD45^{+}$-sorted fraction and the $CD45^{-}$ flow-through fraction. **D** UMAP feature plots of canonical non-myeloid lineage markers used to assess potential contamination. Neuronal markers include *Dcx*, *Gad1* and *Slc17a7*; oligodendrocyte-lineage markers include *Pdgfra*, *Olig2* and *Sox10*. Colour intensity indicates normalized expression. UMI, unique molecular identifier; OPC, oligodendrocyte precursor cell; UMAP, uniform manifold approximation and projection. Source data are provided as a Source Data file.

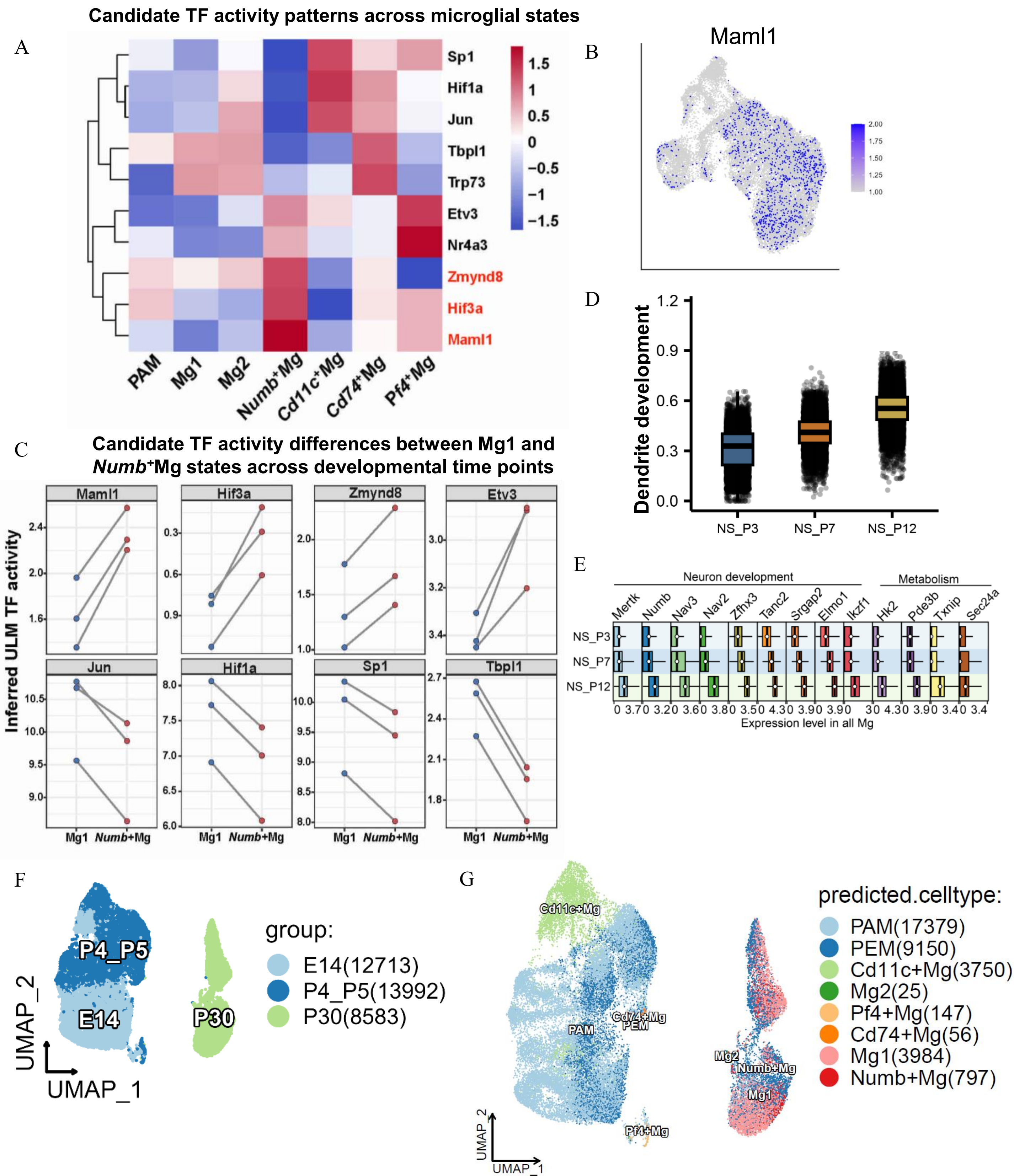

Candidate TF activity patterns across microglial states
A
Sp1
Hif1a
Jun
Tbpl1
Trp73
Etv3
Nr4a3
Zmynd8
Hif3a
Maml1
1.5
1
0.5
0
−0.5
−1
−1.5
PAM
Mg1
Mg2
Numb⁺Mg
Cd11c⁺Mg
Cd74⁺Mg
Pf4⁺Mg
B
Maml1
2.00
1.75
1.50
1.25
1.00
C
Candidate TF activity differences between Mg1 and Numb⁺Mg states across developmental time points
Inferred ULM TF activity
Maml1
Hif3a
Zmynd8
Etv3
Jun
Hif1a
Sp1
Tbpl1
Mg1
Numb+Mg
D
Dendrite development
1.2
0.9
0.6
0.3
0.0
NS_P3
NS_P7
NS_P12
E
Neuron development
Metabolism
Mertk
Numb
Nav3
Nav2
Zfhx3
Tanc2
Srgap2
Elmo1
Ikzf1
Hk2
Pde3b
Txnip
Sec24a
NS_P3
NS_P7
NS_P12
Expression level in all Mg
F
group:
E14(12713)
P4_P5(13992)
P30(8583)
P4_P5
E14
P30
UMAP_2
UMAP_1
G
predicted.celltype:
PAM(17379)
PEM(9150)
Cd11c+Mg(3750)
Mg2(25)
Pf4+Mg(147)
Cd74+Mg(56)
Mg1(3984)
Numb+Mg(797)
Cd11c+Mg
PAM
Cd74+Mg
PEM
Pf4+Mg
Mg2
Numb+Mg
Mg1
UMAP_2
UMAP_1

Fig. S2 | Supplementary analyses of regulatory features and the temporal restriction of the postnatal *Numb*-enriched microglial state. **A** Heatmap of inferred transcription-factor (TF) activities across physiological microglial states. Colours indicate scaled relative activity. Red labels indicate candidate TFs selected for further analysis. **B** Feature plot showing log-normalized *Maml1* expression across physiological microglia. **C** ULM-inferred activities of candidate TFs in Mg1 and the Numb-enriched microglial state across developmental stages. Each line represents one developmental stage and connects the stage-level activity estimates for the two states; blue and red points indicate Mg1 and the Numb-enriched microglial state, respectively. **D** UCell-derived dendrite-development scores across normal-saline microglia at P3, P7 and P12. **E** Cell-level distributions of log-normalized expression of representative neurodevelopmental and metabolic genes across normal-saline microglia at P3, P7 and P12. Colours distinguish individual genes and do not encode an additional quantitative variable. For **D** and **E**, box plots show the median as the centre line, the 25th and 75th percentiles as the box limits, and whiskers extending to the most extreme values within 1.5 times the interquartile range. A total of 8,549, 7,557 and 14,864 cells were analysed at P3, P7 and P12, respectively. Cells were derived from three, two and two biologically independent male mice at P3, P7 and P12, respectively. **F** UMAP representation of the external developmental microglial atlas GSE121654, comprising 12,713 E14. cells, 13,992 P4/P5 cells and 8,583 P30 cells, coloured by developmental stage. **G** Microglial-state annotations transferred to the external atlas after mapping to the reference dataset generated in the present study. *Numb*-enriched Mg, postnatal *Numb*-enriched microglial state; Mg, microglia; NS, normal saline; TF, transcription factor; ULM, univariate linear model; UMAP, uniform manifold approximation and projection.

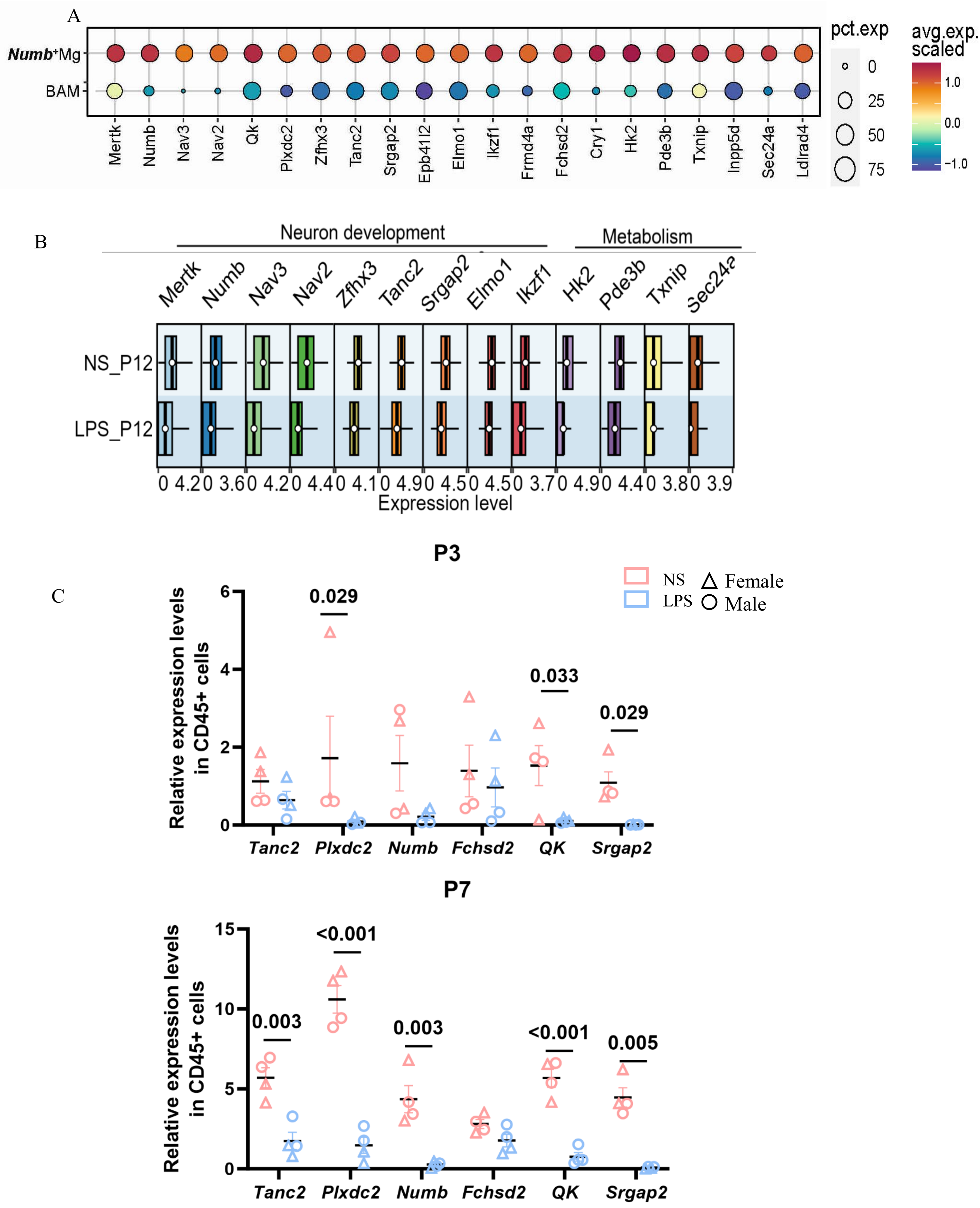
A
Numb+Mg
BAM
Mertk
Numb
Nav3
Nav2
Qk
Plxdc2
Zfhx3
Tanc2
Srgap2
Epb41l2
Elmo1
Ikzf1
Frmd4a
Fchsd2
Cry1
Hk2
Pde3b
Txnip
Inpp5d
Sec24a
Ldlrad4
pct.exp
0
25
50
75
avg.exp.scaled
1.0
0.0
−1.0
B
Neuron development
Metabolism
Mertk
Numb
Nav3
Nav2
Zfhx3
Tanc2
Srgap2
Elmo1
Ikzf1
Hk2
Pde3b
Txnip
Sec24a
NS_P12
LPS_P12
0 4.20 3.60 4.20 4.40 4.10 4.90 4.50 4.50 3.70 4.90 4.40 3.80 3.9
Expression level
C
P3
NS
LPS
Female
Male
Relative expression levels in CD45+ cells
0.029
0.033
0.029
Tanc2
Plxdc2
Numb
Fchsd2
QK
Srgap2
P7
Relative expression levels in CD45+ cells
0.003
<0.001
0.003
<0.001
0.005
Tanc2
Plxdc2
Numb
Fchsd2
QK
Srgap2

Fig. S3 | Neonatal LPS suppresses gene programs associated with the postnatal *Numb*-enriched microglial state during early postnatal development. A Dot plot comparing representative genes associated with the *Numb*-enriched microglial state between *Numb*$^+$microglia and border-associated macrophages (BAMs). Dot size indicates the percentage of cells expressing each gene, and colour indicates scaled average expression. Cells were derived from two biologically independent male mice. **B** Cell-level distributions of log-normalized expression of representative neurodevelopmental and metabolic genes in normal-saline (NS)- and LPS-treated microglia at P12. Box plots show the median (centre line), the 25th and 75th percentiles (box limits), and whiskers extending to the most extreme values within 1.5 times the interquartile range. White dots indicate the mean. A total of 21,353 NS-P12 cells and 16,110 LPS-P12 cells were analysed, with each group comprising two biologically independent male mice. **C** RT–qPCR validation of *Numb*$^+$ Mg-associated genes in CD45$^+$ brain immune cells from NS- and LPS-treated mice at P3 and P7. n = 4 mice per group at each time point. Data are presented as mean ± SEM. At P3, *Tanc2, Fchsd2* and *Qk* were analysed using two-sided unpaired Student's t-tests, *Numb* using a two-sided Welch's t-test, and *Plxdc2* and *Srgap2* using two-sided Wilcoxon rank-sum tests. At P7, *Tanc2, Plxdc2, Numb, Fchsd2* and *Qk* were analysed using two-sided unpaired Student's t-tests, and Srgap2 using a two-sided Wilcoxon rank-sum test. Triangles and circles indicate female and male mice, respectively. *Numb*$^+$Mg, postnatal *Numb*-enriched microglial state; BAM, border associated macrophage; NS, normal saline; LPS, lipopolysaccharide; SEM, standard error of the mean. Source data are provided as a Source Data file.

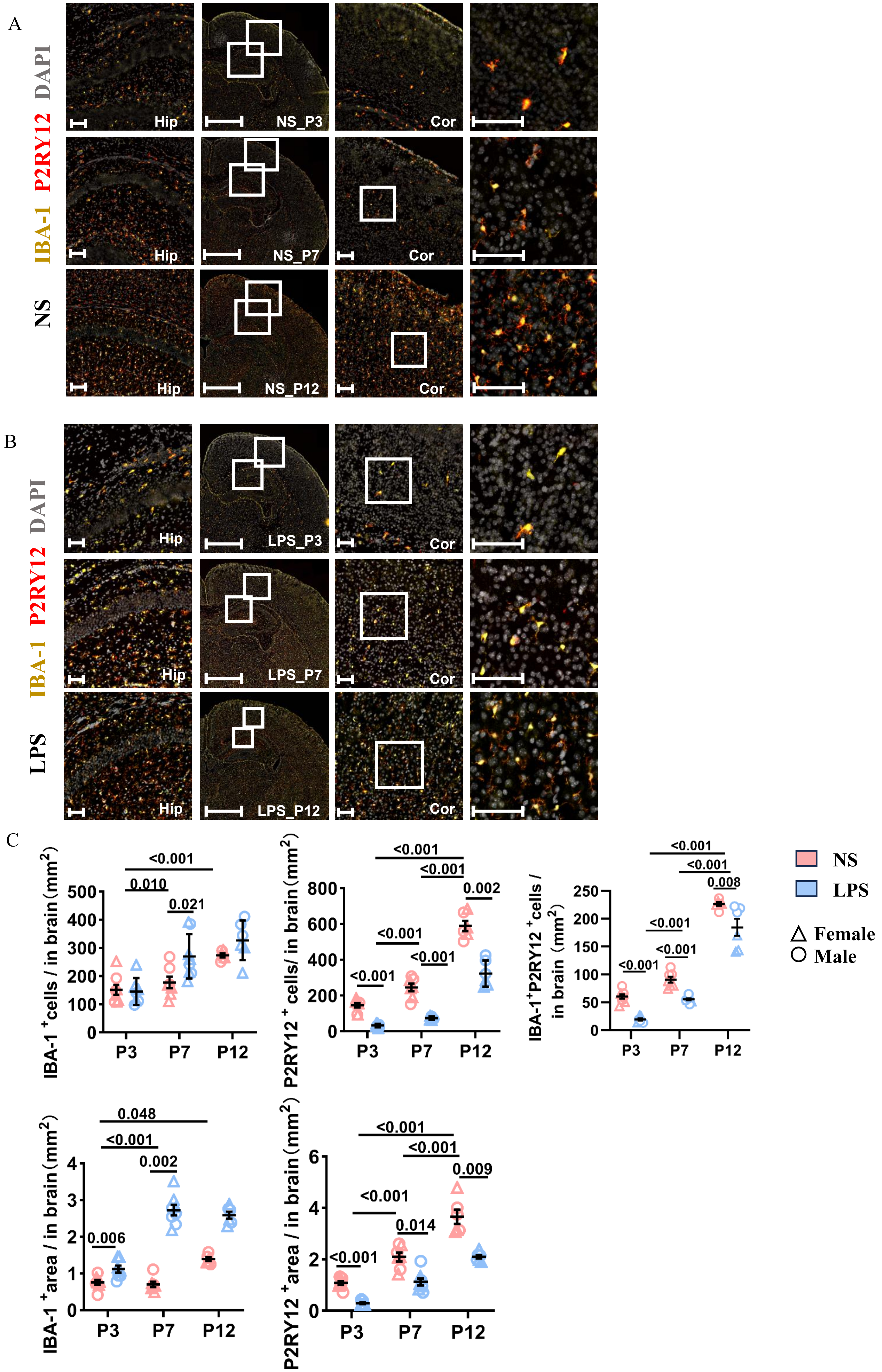
A
IBA-1 P2RY12 DAPI
NS
Hip
NS_P3
Cor
NS_P7
NS_P12
B
IBA-1 P2RY12 DAPI
LPS
Hip
LPS_P3
Cor
LPS_P7
LPS_P12
C
IBA-1 $^+$cells / in brain (mm^2)
P3
P7
P12
<0.001
0.010
0.021
P2RY12 $^+$ cells/ in brain (mm^2)
<0.001
<0.001
0.002
<0.001
<0.001
<0.001
IBA-1$^+$P2RY12 $^+$cells / in brain (mm^2)
<0.001
<0.001
0.008
<0.001
<0.001
<0.001
NS
LPS
Female
Male
IBA-1 $^+$area / in brain (mm^2)
0.048
<0.001
0.002
0.006
P2RY12 $^+$area / in brain (mm^2)
<0.001
<0.001
0.009
<0.001
0.014
<0.001

Fig. S4 | Temporal changes in IBA-1$^+$ and P2RY12$^+$ microglia after neonatal LPS exposure. A–B Representative immunofluorescence images of IBA-1 (yellow), P2RY12 (red) and DAPI (grey) in brain sections from NS-treated (**A**) and LPS-treated (**B**) mice at P3, P7 and P12. Hippocampal (Hip) and cortical (Cor) regions are shown, with boxed areas indicating the magnified fields. Scale bars, 1 mm for whole-section images in the second column and 100 μm for all other images. **C** Quantification of IBA-1$^+$ cell density, P2RY12$^+$ cell density, IBA-1$^+$P2RY12$^+$ cell density, IBA-1$^+$ area and P2RY12$^+$ area across P3, P7 and P12. P3-NS, n = 8 mice; P3-LPS, n = 7 mice; P7-NS, n = 7 mice; P7-LPS, n = 8 mice; P12-NS, n = 7 mice; P12-LPS, n = 6 mice. Data are presented as mean ± SEM. Overall effects were assessed using Scheirer–Ray–Hare tests. NS and LPS groups were compared at each time point using two-sided Wilcoxon rank-sum tests. Temporal differences within the LPS group were assessed using Kruskal–Wallis tests followed by Dunn's post hoc tests. Triangles and circles indicate female and male mice, respectively. NS, normal saline; LPS, lipopolysaccharide; SEM, standard error of the mean. Source data are provided as a Source Data file.

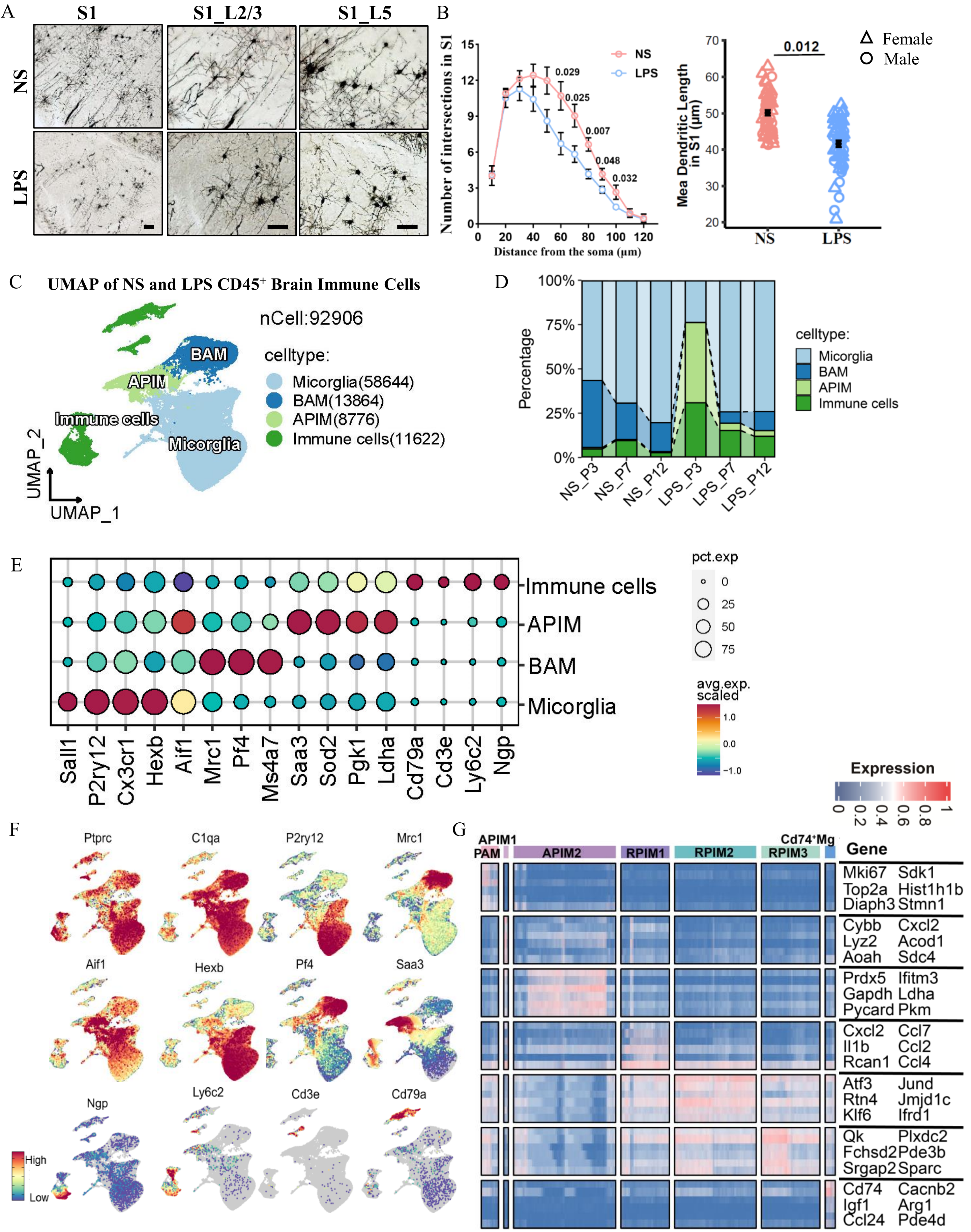

A
S1
S1_L2/3
S1_L5
NS
LPS
B
Number of intersections in S1
Distance from the soma (μm)
NS
LPS
0.029
0.025
0.007
0.048
0.032
Mea Dendritic Length in S1 (μm)
0.012
Female
Male
C
UMAP of NS and LPS CD45+ Brain Immune Cells
nCell:92906
celltype:
Micorglia(58644)
BAM(13864)
APIM(8776)
Immune cells(11622)
UMAP_1
UMAP_2
D
Percentage
NS_P3
NS_P7
NS_P12
LPS_P3
LPS_P7
LPS_P12
celltype:
Micorglia
BAM
APIM
Immune cells
E
Immune cells
APIM
BAM
Micorglia
Sall1
P2ry12
Cx3cr1
Hexb
Aif1
Mrc1
Pf4
Ms4a7
Saa3
Sod2
Pgk1
Ldha
Cd79a
Cd3e
Ly6c2
Ngp
pct.exp
avg.exp.scaled
F
Ptprc
C1qa
P2ry12
Mrc1
Aif1
Hexb
Pf4
Saa3
Ngp
Ly6c2
Cd3e
Cd79a
High
Low
G
Expression
APIM1
PAM
APIM2
RPIM1
RPIM2
RPIM3
Cd74+Mg
Gene
Mki67 Sdk1
Top2a Hist1h1b
Diaph3 Stmn1
Cybb Cxcl2
Lyz2 Acod1
Aoah Sdc4
Prdx5 Ifitm3
Gapdh Ldha
Pycard Pkm
Cxcl2 Ccl7
Il1b Ccl2
Rcan1 Ccl4
Atf3 Jund
Rtn4 Jmjd1c
Klf6 Ifrd1
Qk Plxdc2
Fchsd2 Pde3b
Srgap2 Sparc
Cd74 Cacnb2
Igf1 Arg1
Ccl24 Pde4d

Fig. S5 | Neonatal LPS exposure is associated with altered S1 dendritic morphology and remodelling of CD45⁺ brain immune cells. A Representative Golgi–Cox images of neurons in the primary somatosensory cortex (S1) from NS- and LPS-treated mice at P21. S1 overview images and representative neurons from S1 layers 2/3 and 5 are shown. Scale bars, 100 µm. **B** Sholl analysis and mean dendritic length of S1 neurons. n = 5 mice per group. Measurements from multiple neurons were averaged within each mouse. Data are presented as mean ± SEM of mouse-level averages, which were used for statistical analysis. Individual neuron measurements are shown in the dendritic-length plot for visualization. At radial distances of 10 and 20 µm, comparisons were performed using two-sided unpaired Welch's t-tests; at 30–90 µm, two-sided unpaired Student's t-tests were used; and at 100–120 µm, two-sided Wilcoxon rank-sum tests were used. Total dendritic length was compared using a two-sided Wilcoxon rank-sum test. Triangles and circles indicate female and male mice, respectively. **C** UMAP of 92,906 $CD45^+$ brain immune cells from 13 independent male biological samples across the NS and LPS groups at P3, P7 and P12, colored by major cell compartment. **D** Relative abundance of major immune-cell compartments across experimental groups and developmental stages. **E** Dot plot of representative markers distinguishing parenchymal microglia, APIM, BAMs and peripheral immune cells. Dot size indicates the percentage of cells expressing each gene, and color indicates scaled average expression. **F** Feature plots of representative identity and lineage markers; color indicates normalized expression. **G** Heatmap of selected marker genes across LPS-associated microglial states and related clusters. Columns represent cells and rows represent genes; expression was min–max normalized per gene to 0–1. APIM, acute post-inflammatory microglia; RPIM, recovery-phase inflammatory microglia; BAM, border-associated macrophages; NS, normal saline; LPS, lipopolysaccharide; SEM, standard error of the mean; UMAP, uniform manifold approximation and projection. Source data are provided as a Source Data file.

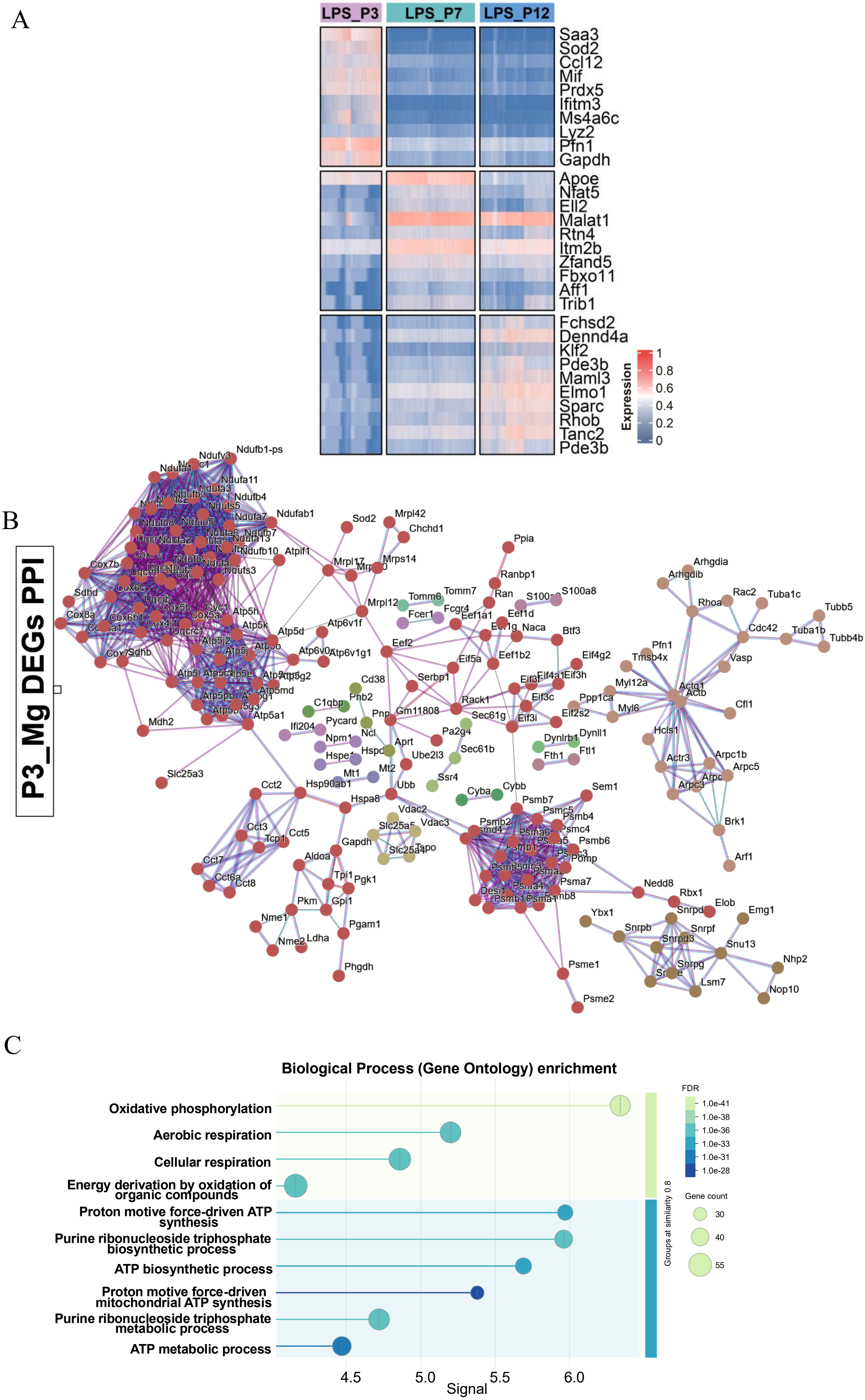
A
LPS_P3
LPS_P7
LPS_P12
Saa3
Sod2
Ccl12
Mif
Prdx5
Ifitm3
Ms4a6c
Lyz2
Pfn1
Gapdh
Apoe
Nfat5
Ell2
Malat1
Rtn4
Itm2b
Zfand5
Fbxo11
Aff1
Trib1
Fchsd2
Dennd4a
Klf2
Pde3b
Maml3
Elmo1
Sparc
Rhob
Tanc2
Pde3b
Expression
1
0.8
0.6
0.4
0.2
0
B
P3_Mg DEGs PPI
C
Biological Process (Gene Ontology) enrichment
Oxidative phosphorylation
Aerobic respiration
Cellular respiration
Energy derivation by oxidation of organic compounds
Proton motive force-driven ATP synthesis
Purine ribonucleoside triphosphate biosynthetic process
ATP biosynthetic process
Proton motive force-driven mitochondrial ATP synthesis
Purine ribonucleoside triphosphate metabolic process
ATP metabolic process
4.5
5.0
5.5
6.0
Signal
FDR
1.0e-41
1.0e-38
1.0e-36
1.0e-33
1.0e-31
1.0e-28
Groups at similarity 0.8
Gene count
30
40
55

Fig. S6 | Temporal transcriptional dynamics and functional signatures of LPS-associated microglia. A Heatmap showing the top 10 stage-enriched genes in LPS-associated microglia at P3, P7 and P12. Rows represent genes and columns represent cells grouped by developmental stage. Expression values were min–max normalized per gene to 0–1. Cells were derived from two biologically independent male mice at each stage. **B** Protein–protein interaction (PPI) network constructed from P3-enriched differentially expressed genes using STRING. Nodes represent the encoded proteins, edges indicate known or predicted protein–protein associations, and node colours indicate distinct interaction modules. **C** Gene Ontology biological process (GO-BP) enrichment analysis of proteins represented in the P3 microglial PPI network. Enrichment was assessed using a one-sided hypergeometric test, with P values adjusted using the Benjamini–Hochberg method. Dot size indicates gene count, colour indicates the false discovery rate (FDR), and the horizontal axis shows the STRING enrichment signal. PPI, protein–protein interaction; GO-BP, Gene Ontology biological process; FDR, false discovery rate.

A

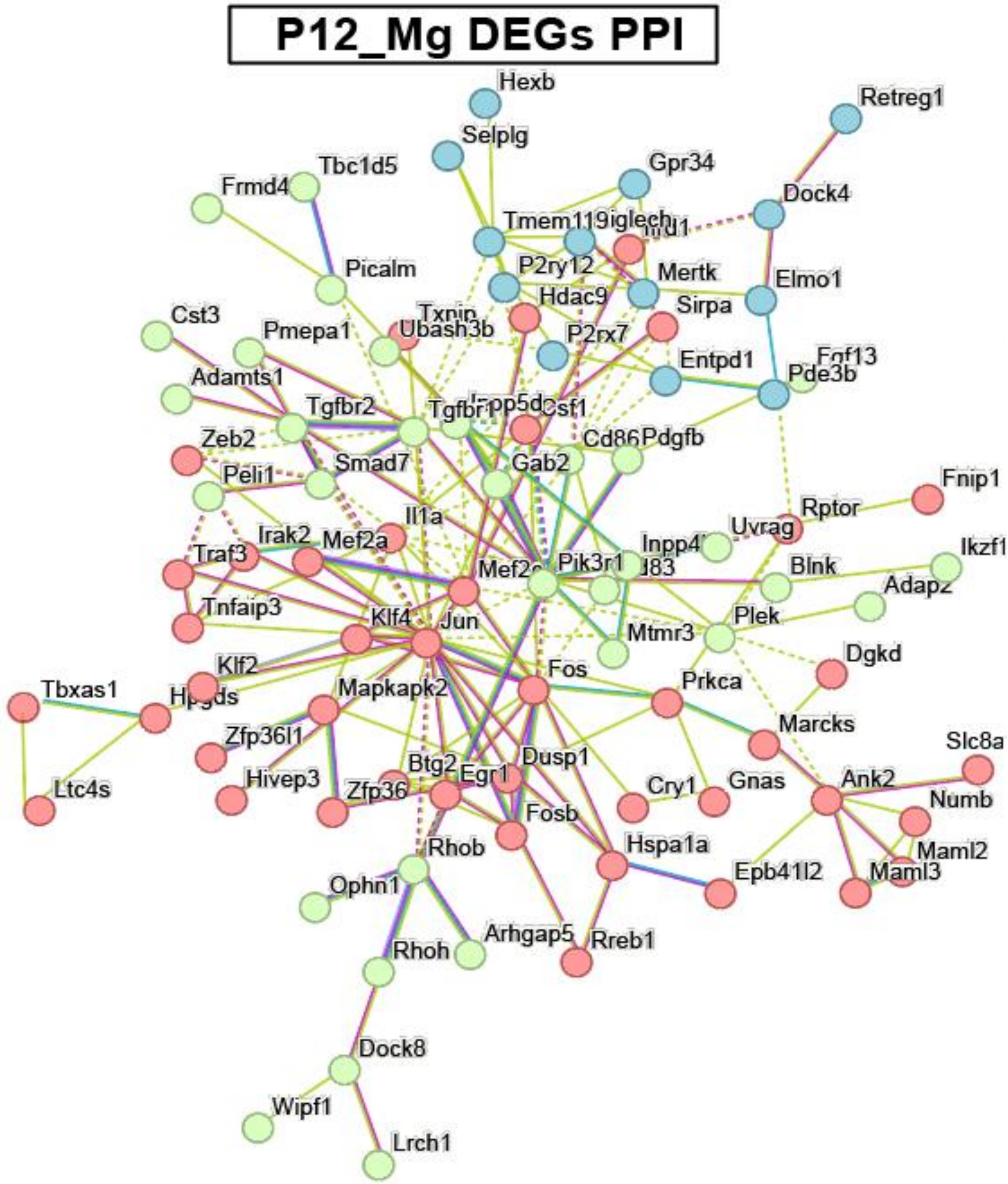
P12_Mg DEGs PPI
Hexb
Retreg1
Selplg
Gpr34
Tbc1d5
Frmd4
Dock4
Tmem119
P2ry12
Mertk
Elmo1
Picalm
Hdac9
Sirpa
Cst3
Txnip
Ubash3b
P2rx7
Pmepa1
Entpd1
Pde3b
Adamts1
Tgfbr2
Csf1
Zeb2
Cd86
Pdgfb
Smad7
Gab2
Peli1
Fnip1
Rptor
Il1a
Uvrag
Irak2
Mef2a
Ikzf1
Traf3
Pik3r1
Blnk
Adap2
Tnfaip3
Klf4
Jun
Mtmr3
Plek
Klf2
Dgkd
Fos
Tbxas1
Prkca
Mapkapk2
Marcks
Zfp36l1
Slc8a
Btg2
Dusp1
Hivep3
Egr1
Cry1
Gnas
Ank2
Ltc4s
Zfp36
Numb
Fosb
Maml2
Hspa1a
Maml3
Rhob
Epb41l2
Ophn1
Arhgap5
Rreb1
Rhoh
Dock8
Wipf1
Lrch1

B

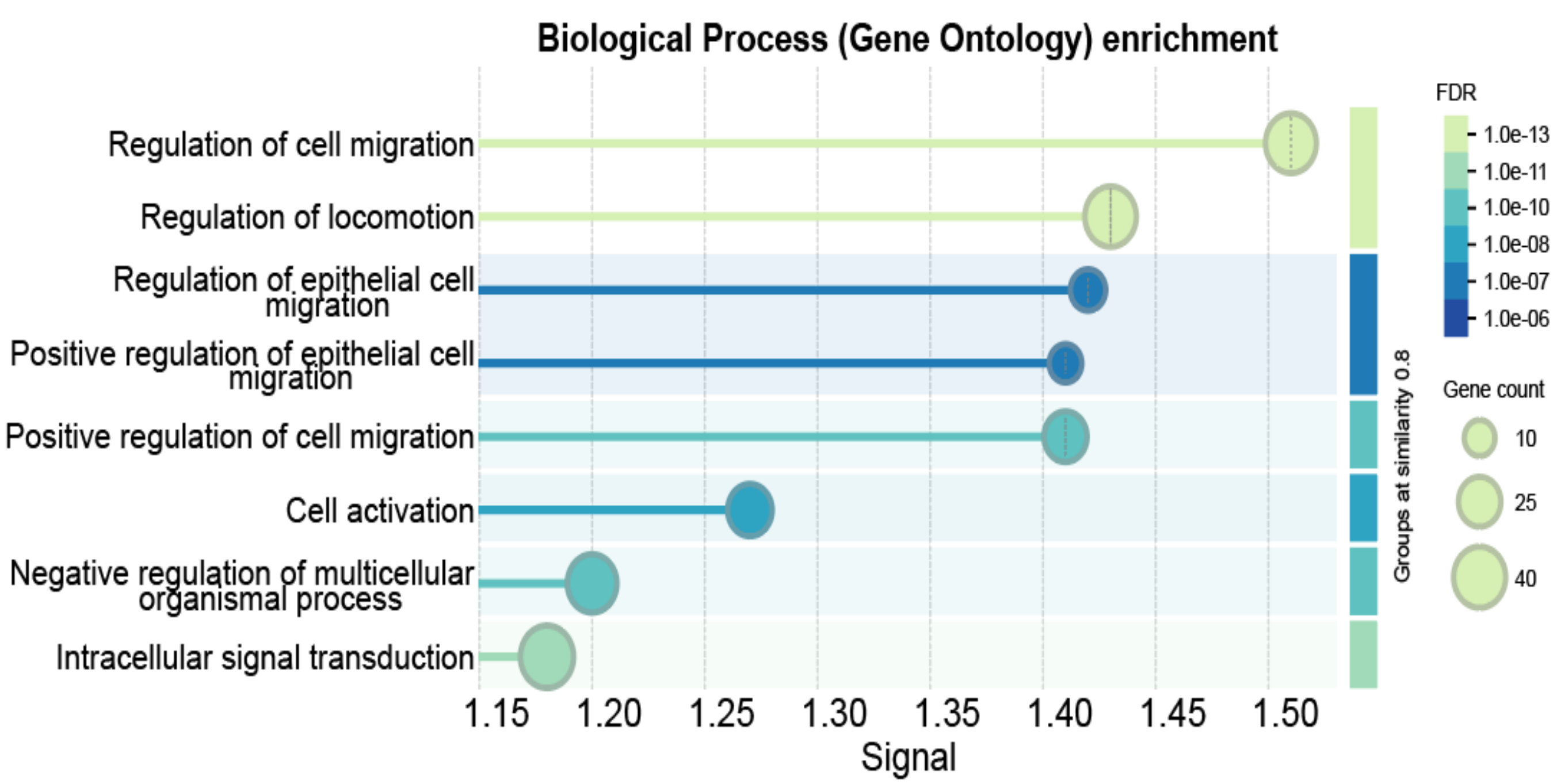
Biological Process (Gene Ontology) enrichment
Regulation of cell migration
Regulation of locomotion
Regulation of epithelial cell migration
Positive regulation of epithelial cell migration
Positive regulation of cell migration
Cell activation
Negative regulation of multicellular organismal process
Intracellular signal transduction
1.15
1.20
1.25
1.30
1.35
1.40
1.45
1.50
Signal
Groups at similarity 0.8
FDR
1.0e-13
1.0e-11
1.0e-10
1.0e-08
1.0e-07
1.0e-06
Gene count
10
25
40

Fig. S7. Functional interaction network and GO enrichment of differentially expressed genes in P12 LPS-associated microglia. **A** Protein–protein interaction (PPI) network of differentially expressed genes (DEGs) in P12 LPS-associated microglia, generated using STRING. Nodes represent proteins encoded by DEGs, edges indicate known or predicted protein–protein associations, and node colors indicate distinct interaction modules. **B** GO-BP enrichment analysis of proteins in the P12 microglial PPI network using STRING. Enrichment was assessed using a one-sided hypergeometric test, with *P* values adjusted using the Benjamini–Hochberg method. Dot size indicates gene count, color indicates false discovery rate (FDR), and the horizontal axis shows the STRING enrichment signal. GO-BP, Gene Ontology biological process.

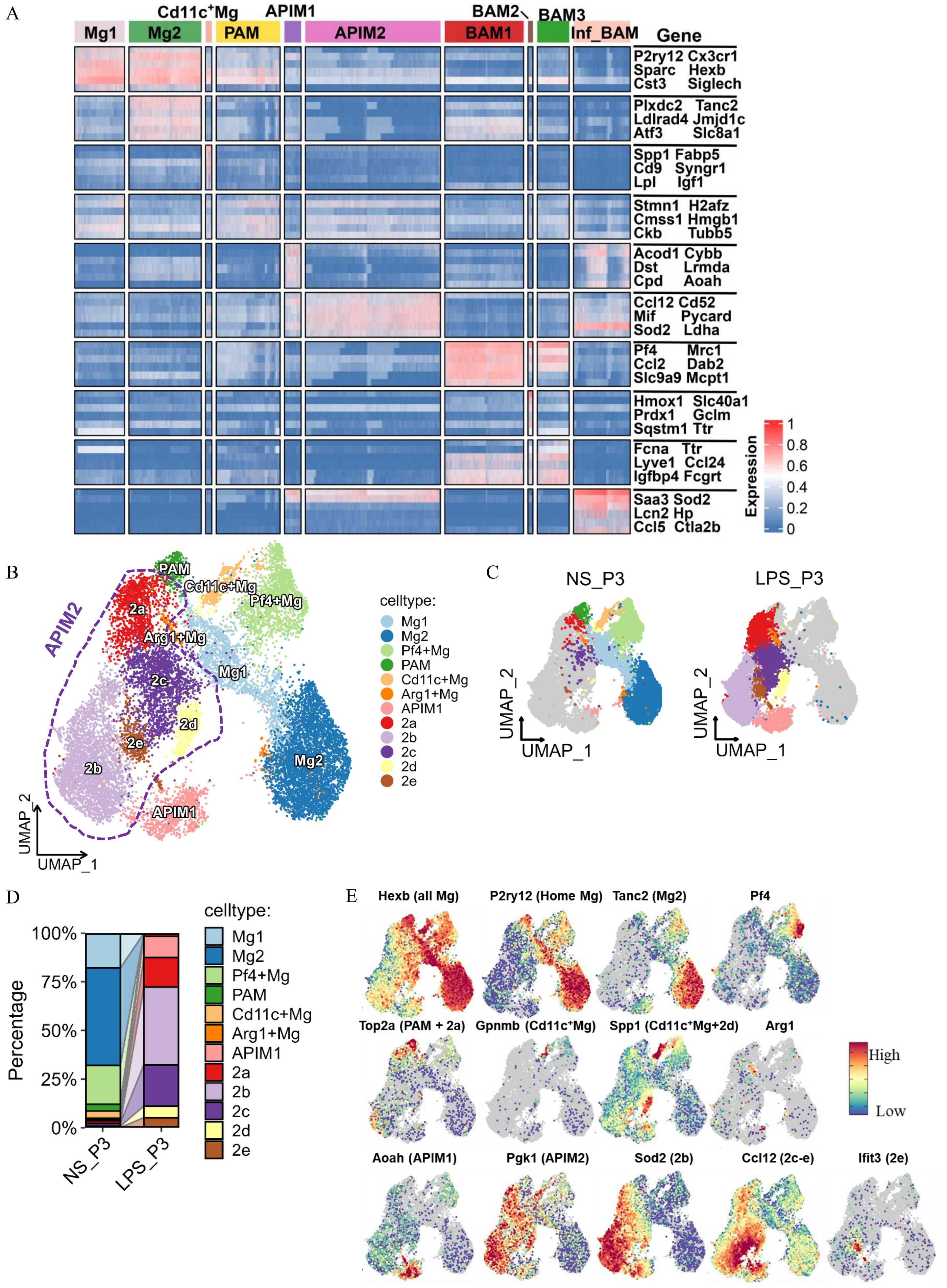

A
Cd11c+Mg
APIM1
BAM2
BAM3
Mg1
Mg2
PAM
APIM2
BAM1
Inf_BAM
Gene
P2ry12 Cx3cr1
Sparc Hexb
Cst3 Siglech
Plxdc2 Tanc2
Ldlrad4 Jmjd1c
Atf3 Slc8a1
Spp1 Fabp5
Cd9 Syngr1
Lpl Igf1
Stmn1 H2afz
Cmss1 Hmgb1
Ckb Tubb5
Acod1 Cybb
Dst Lrmda
Cpd Aoah
Ccl12 Cd52
Mif Pycard
Sod2 Ldha
Pf4 Mrc1
Ccl2 Dab2
Slc9a9 Mcpt1
Hmox1 Slc40a1
Prdx1 Gclm
Sqstm1 Ttr
Fcna Ttr
Lyve1 Ccl24
Igfbp4 Fcgrt
Saa3 Sod2
Lcn2 Hp
Ccl5 Ctla2b
Expression
1
0.8
0.6
0.4
0.2
0
B
APIM2
PAM
Cd11c+Mg
Pf4+Mg
2a
Arg1+Mg
Mg1
2c
2d
2e
2b
Mg2
APIM1
UMAP_2
UMAP_1
celltype:
Mg1
Mg2
Pf4+Mg
PAM
Cd11c+Mg
Arg1+Mg
APIM1
2a
2b
2c
2d
2e
C
NS_P3
LPS_P3
UMAP_2
UMAP_1
D
Percentage
100%
75%
50%
25%
0%
NS_P3
LPS_P3
celltype:
Mg1
Mg2
Pf4+Mg
PAM
Cd11c+Mg
Arg1+Mg
APIM1
2a
2b
2c
2d
2e
E
Hexb (all Mg)
P2ry12 (Home Mg)
Tanc2 (Mg2)
Pf4
Top2a (PAM + 2a)
Gpnmb (Cd11c+Mg)
Spp1 (Cd11c+Mg+2d)
Arg1
High
Low
Aoah (APIM1)
Pgk1 (APIM2)
Sod2 (2b)
Ccl12 (2c-e)
Ifit3 (2e)

Fig. S8 | High-resolution subclustering resolves APIM2 subclusters at P3 after neonatal LPS exposure. A Heatmap showing selected marker-gene expression across physiological microglial states, APIM populations and BAM-related populations in the integrated P3 myeloid dataset. Columns represent cells and rows represent genes; expression was min–max normalized per gene to 0–1. **B** UMAP of the integrated P3 myeloid dataset after high-resolution subclustering of APIM2, resolving subclusters 2a–2e. The purple dashed outline indicates the original APIM2 compartment. **C** UMAPs highlighting NS- and LPS-derived cells at P3. Cells from the indicated condition are coloured by annotated population, whereas cells from the other condition are shown in grey. **D** Relative abundance of annotated populations in NS- and LPS-treated brains at P3 after APIM2 subclustering. **E** Feature plots of representative markers distinguishing physiological microglial states, APIM2 subclusters and BAM-related populations; colour indicates normalized expression. The integrated dataset comprised three biologically independent NS male mice and two biologically independent LPS male mice. Mg, microglia; PAM, proliferative-region-associated microglia; APIM, acute post-inflammatory microglia; BAM, border-associated macrophage; Inf_BAM, inflammation-associated BAM; NS, normal saline; LPS, lipopolysaccharide; UMAP, uniform manifold approximation and projection.

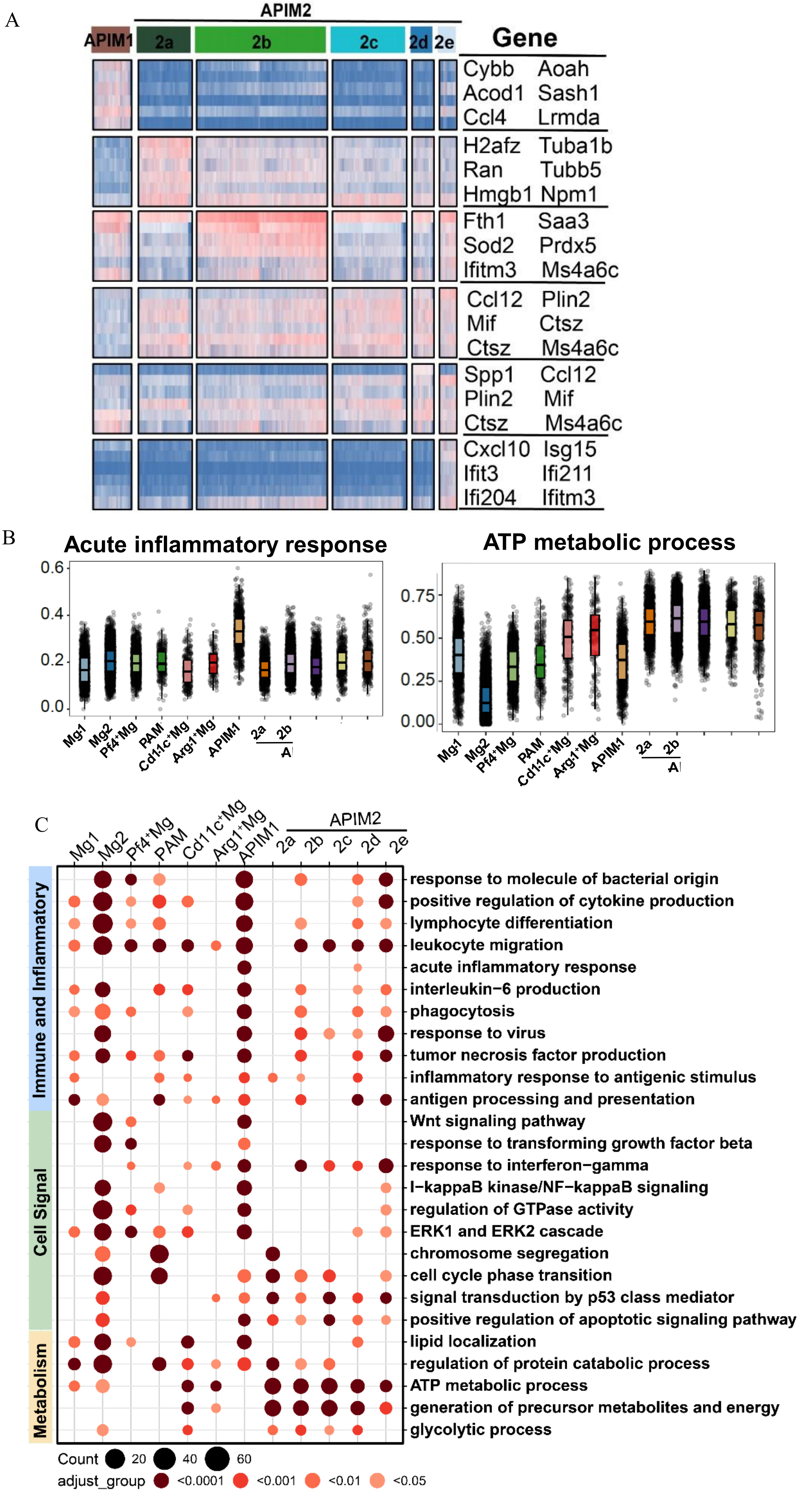

A
APIM2
APIM1
2a
2b
2c
2d
2e
Gene
Cybb Aoah
Acod1 Sash1
Ccl4 Lrmda
H2afz Tuba1b
Ran Tubb5
Hmgb1 Npm1
Fth1 Saa3
Sod2 Prdx5
Ifitm3 Ms4a6c
Ccl12 Plin2
Mif Ctsz
Ctsz Ms4a6c
Spp1 Ccl12
Plin2 Mif
Ctsz Ms4a6c
Cxcl10 Isg15
Ifit3 Ifi211
Ifi204 Ifitm3
B
Acute inflammatory response
0.6
0.4
0.2
0.0
Mg1
Mg2
Pf4+Mg
PAM
Cd11c+Mg
Arg1+Mg
APIM1
2a
2b
ATP metabolic process
0.75
0.50
0.25
0.00
C
APIM2
Mg1
Mg2
Pf4+Mg
PAM
Cd11c+Mg
Arg1+Mg
APIM1
2a
2b
2c
2d
2e
Immune and Inflammatory
Cell Signal
Metabolism
response to molecule of bacterial origin
positive regulation of cytokine production
lymphocyte differentiation
leukocyte migration
acute inflammatory response
interleukin−6 production
phagocytosis
response to virus
tumor necrosis factor production
inflammatory response to antigenic stimulus
antigen processing and presentation
Wnt signaling pathway
response to transforming growth factor beta
response to interferon−gamma
I−kappaB kinase/NF−kappaB signaling
regulation of GTPase activity
ERK1 and ERK2 cascade
chromosome segregation
cell cycle phase transition
signal transduction by p53 class mediator
positive regulation of apoptotic signaling pathway
lipid localization
regulation of protein catabolic process
ATP metabolic process
generation of precursor metabolites and energy
glycolytic process
Count 20 40 60
adjust_group <0.0001 <0.001 <0.01 <0.05

Fig. S9 | Transcriptional and functional heterogeneity of APIM2 subclusters at P3 after neonatal LPS exposure. **A** Heatmap showing selected marker-gene expression across APIM1 and APIM2 subclusters. Columns represent cells and rows represent genes; expression was min–max normalized per gene to 0–1. **B** UCell-derived acute-inflammatory-response and ATP-metabolic-process scores across annotated microglial populations and APIM2 subclusters. Box plots show the median (centre line), the 25th and 75th percentiles (box limits), and whiskers extending to the most extreme values within 1.5 times the interquartile range. Grey points represent individual cells. Cell numbers were 1,553, 4,314, 1,726, 307, 314, 144, 957, 3,257, 1,338, 1,852, 542 and 432 for Mg1, Mg2, $Pf4^{+}$ Mg, PAM, $CD11c^{+}$ Mg, $Arg1^{+}$ Mg, APIM1, 2a, 2b, 2c, 2d and 2e, respectively. **C** Selected GO-BP terms enriched among marker genes for annotated microglial populations and APIM2 subclusters. Dot size indicates gene count and colour indicates the Benjamini–Hochberg-adjusted P value. Enrichment was assessed using a one-sided hypergeometric test. Cells were derived from three NS and two LPS biologically independent male mice. Mg, microglia; PAM, proliferative-region-associated microglia; APIM, acute post-inflammatory microglia; GO-BP, Gene Ontology biological process.

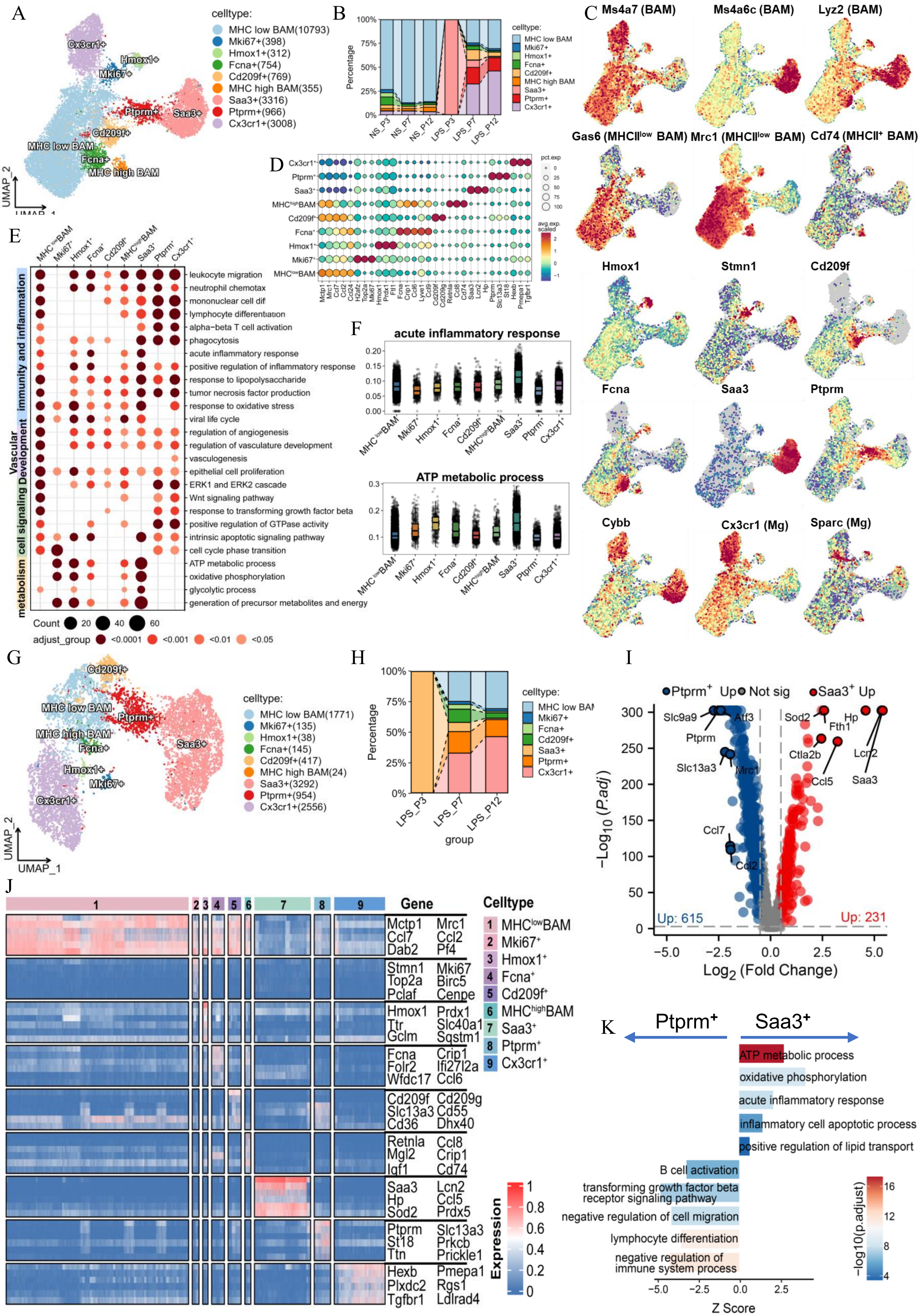

A
celltype:
MHC low BAM(10793)
Mki67+(398)
Hmox1+(312)
Fcna+(754)
Cd209f+(769)
MHC high BAM(355)
Saa3+(3316)
Ptprm+(966)
Cx3cr1+(3008)
UMAP_2
UMAP_1
B
Percentage
celltype
NS_P3
NS_P7
NS_P12
LPS_P3
LPS_P7
LPS_P12
C
Ms4a7 (BAM)
Ms4a6c (BAM)
Lyz2 (BAM)
Gas6 (MHCIIlow BAM)
Mrc1 (MHCIIlow BAM)
Cd74 (MHCII+ BAM)
Hmox1
Stmn1
Cd209f
Fcna
Saa3
Ptprm
Cybb
Cx3cr1 (Mg)
Sparc (Mg)
D
pct.exp
avg.exp.scaled
E
immunity and inflammation
Vascular Development
cell signaling
metabolism
leukocyte migration
neutrophil chemotax
mononuclear cell dif
lymphocyte differentiation
alpha−beta T cell activation
phagocytosis
acute inflammatory response
positive regulation of inflammatory response
response to lipopolysaccharide
tumor necrosis factor production
response to oxidative stress
viral life cycle
regulation of angiogenesis
regulation of vasculature development
vasculogenesis
epithelial cell proliferation
ERK1 and ERK2 cascade
Wnt signaling pathway
response to transforming growth factor beta
positive regulation of GTPase activity
intrinsic apoptotic signaling pathway
cell cycle phase transition
ATP metabolic process
oxidative phosphorylation
glycolytic process
generation of precursor metabolites and energy
Count 20 40 60
adjust_group <0.0001 <0.001 <0.01 <0.05
F
acute inflammatory response
ATP metabolic process
G
celltype:
MHC low BAM(1771)
Mki67+(135)
Hmox1+(38)
Fcna+(145)
Cd209f+(417)
MHC high BAM(24)
Saa3+(3292)
Ptprm+(954)
Cx3cr1+(2556)
UMAP_2
UMAP_1
H
Percentage
group
LPS_P3
LPS_P7
LPS_P12
I
Ptprm+ Up
Not sig
Saa3+ Up
Up: 615
Up: 231
−Log10 (P.adj)
Log2 (Fold Change)
J
Gene
Celltype
1 MHClowBAM
2 Mki67+
3 Hmox1+
4 Fcna+
5 Cd209f+
6 MHChighBAM
7 Saa3+
8 Ptprm+
9 Cx3cr1+
Expression
K
Ptprm+
Saa3+
ATP metabolic process
oxidative phosphorylation
acute inflammatory response
inflammatory cell apoptotic process
positive regulation of lipid transport
B cell activation
transforming growth factor beta receptor signaling pathway
negative regulation of cell migration
lymphocyte differentiation
negative regulation of immune system process
Z Score
−log10(p.adjust)

Fig. S10 | LPS drives transcriptional reprogramming and metabolic changes in border-associated macrophages during postnatal development. **A** UMAP of 20,671 BAMs from 13 biologically independent male mice across NS and LPS groups at P3, P7 and P12, coloured by BAM population. NS-P3, n = 3 mice; all other groups, n = 2 mice. **B** Relative abundance of BAM populations across groups and stages. **C** Feature plots of representative markers; colour indicates normalized expression. **D** Dot plot of selected markers. Dot size indicates the percentage of expressing cells and colour indicates scaled average expression. **E** Selected GO-BP terms enriched among BAM marker genes. Dot size indicates gene count and colour indicates Benjamini–Hochberg-adjusted P value. Enrichment was assessed using a one-sided hypergeometric test. **F** UCell-derived acute-inflammatory-response and ATP-metabolic-process scores. Box plots show the median, 25th and 75th percentiles, and whiskers extending to 1.5 times the interquartile range. Grey points represent cells. Cell numbers were 10,793, 398, 312, 754, 769, 355, 3,316, 966 and 3,008 for MHC-low, *Mki67*$^+$, *Hmox1*$^+$, *Fcna*$^+$, *Cd209f*$^+$, MHC-high, *Saa3*$^+$, *Ptprm*$^+$ and *Cx3cr1*$^+$ BAMs, respectively. **G** UMAP of 9,332 LPS-derived BAMs across P3, P7 and P12, coloured by population. **H** Relative abundance of BAM populations across stages after LPS exposure. **I** Differential expression between *Ptprm*$^+$ and *Saa3*$^+$ BAMs. Two-sided Wilcoxon rank-sum tests with Bonferroni correction were used; blue and red indicate genes upregulated in *Ptprm*$^+$ and *Saa3*$^+$ BAMs, respectively (adjusted $P < 0.01$; $|\log_2 \text{fold change}| > 0.3$). **J** Heatmap of selected marker genes. Columns represent cells and rows represent genes; expression was min–max normalized per gene to 0–1. **K** GO-BP enrichment of genes upregulated in *Ptprm*$^+$ and *Saa3*$^+$ BAMs. Enrichment was assessed using a one-sided hypergeometric test with Benjamini–Hochberg adjustment. Bar length indicates enrichment Z score and colour indicates $-\log_{10}$(adjusted P value); negative and positive values indicate *Ptprm*$^+$ and *Saa3*$^+$ directions, respectively. BAM, border-associated macrophage; GO-BP, Gene Ontology biological process; MHC, major histocompatibility complex; NS, normal saline; LPS, lipopolysaccharide; UMAP, uniform manifold approximation and projection.

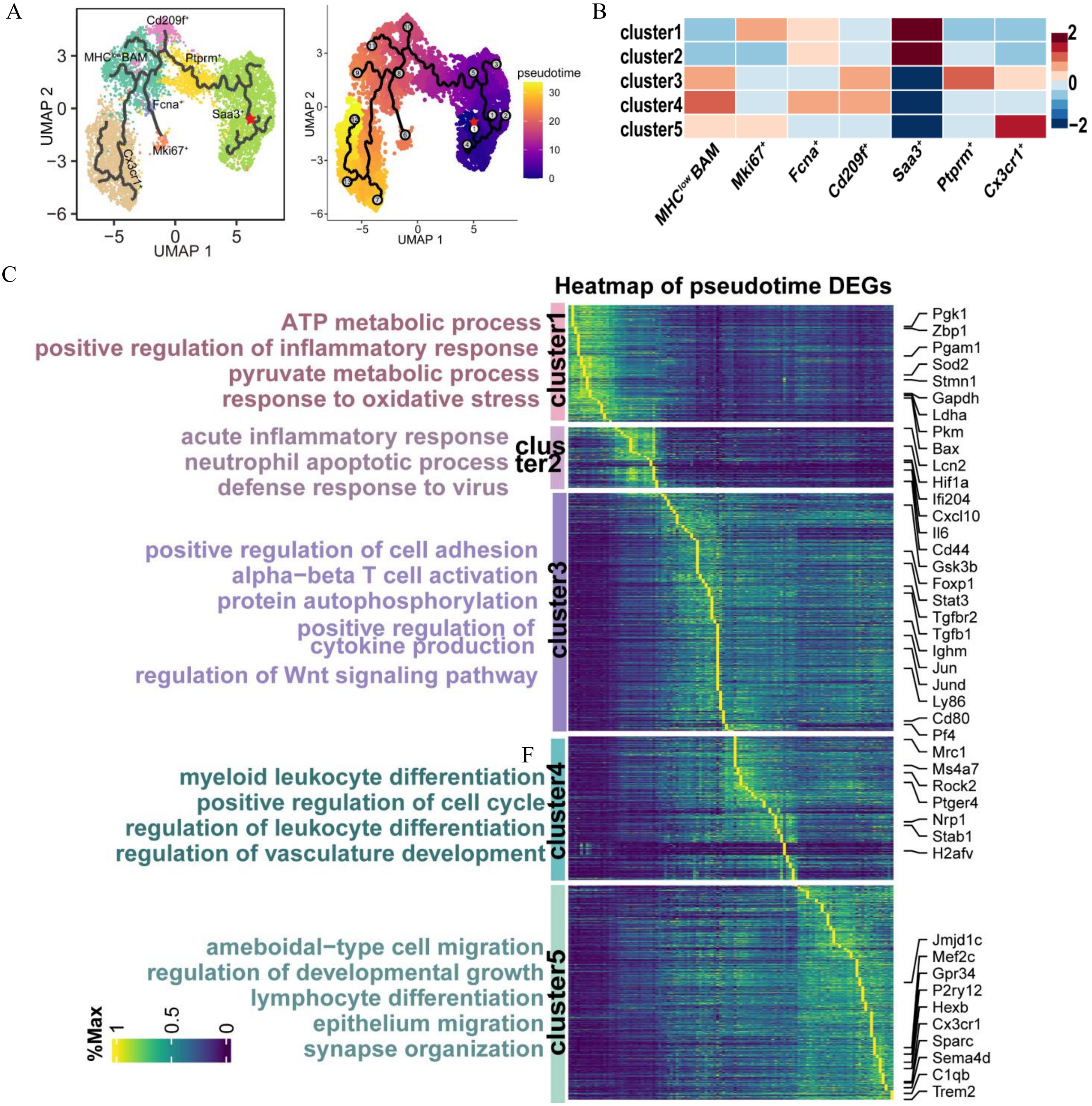

A
Cd209f+
MHClow BAM
Ptprm+
Fcna+
Saa3+
Mki67+
Cx3cr1+
UMAP 1
UMAP 2
pseudotime
B
cluster1
cluster2
cluster3
cluster4
cluster5
MHClow BAM
Mki67+
Fcna+
Cd209f+
Saa3+
Ptprm+
Cx3cr1+
C
Heatmap of pseudotime DEGs
ATP metabolic process
positive regulation of inflammatory response
pyruvate metabolic process
response to oxidative stress
acute inflammatory response
neutrophil apoptotic process
defense response to virus
positive regulation of cell adhesion
alpha−beta T cell activation
protein autophosphorylation
positive regulation of cytokine production
regulation of Wnt signaling pathway
F
myeloid leukocyte differentiation
positive regulation of cell cycle
regulation of leukocyte differentiation
regulation of vasculature development
ameboidal−type cell migration
regulation of developmental growth
lymphocyte differentiation
epithelium migration
synapse organization
%Max
Pgk1
Zbp1
Pgam1
Sod2
Stmn1
Gapdh
Ldha
Pkm
Bax
Lcn2
Hif1a
Ifi204
Cxcl10
Il6
Cd44
Gsk3b
Foxp1
Stat3
Tgfbr2
Tgfb1
Ighm
Jun
Jund
Ly86
Cd80
Pf4
Mrc1
Ms4a7
Rock2
Ptger4
Nrp1
Stab1
H2afv
Jmjd1c
Mef2c
Gpr34
P2ry12
Hexb
Cx3cr1
Sparc
Sema4d
C1qb
Trem2

Fig. S11 | Pseudotime trajectory and gene-program modules of BAM reprogramming from P3 to P12 after neonatal LPS exposure. A Pseudotime trajectory of BAMs from LPS-treated brains at P3, P7 and P12. UMAP plots show cells coloured by BAM population (left) or pseudotime (right; purple, early; yellow, late). Black lines indicate the inferred principal graph, and the red star indicates the root node assigned within the *Saa3*$^{+}$ BAM population. Cells were derived from two biologically independent male mice at each stage. **B** Heatmap showing the mean scaled expression of pseudotime-associated gene modules across BAM populations. Expression was Z-score scaled per module; colour indicates relative expression. **C** Heatmap showing representative pseudotime-varying genes ordered along the inferred trajectory (right) and enriched GO-BP terms for each co-expression module (left). Colour indicates scaled gene expression. BAM, border-associated macrophage; GO-BP, Gene Ontology biological process; UMAP, uniform manifold approximation and projection.

Supplementary Table 1 Primer information

Gene	Primer Direction	Sequence (5'→3')
Tanc2	Forward	CTTCTTTGTCCATCAGGGCAAT
Tanc2	Reverse	ACAAGCAGGGTCGTACTCC
Plxdc2	Forward	TTCATTCCAAGGAAAAGCGTTTG
Plxdc2	Reverse	GCCGCAGCAGGAGTTATGTTA
Numb	Forward	GTTTTCTCGTCCACAACTCTGAG
Numb	Reverse	AAAGCAGTGAAGGCCGTTCT
Fchsd2	Forward	CAAGTAAGTCACATTCTGCCTGA
Fchsd2	Reverse	CCGCCGAGGAAGGTGAAAG
Qk	Forward	ACTGCCATTTAACGTGTCATTGT
Qk	Reverse	CTGGACGAAGAAATTAGCAGAGT
Srgap2	Forward	GCTTGGCTTGTAGCTTTGTGATG
Srgap2	Reverse	CTAAGAGGAGAGCGAACCAGC
