# Supplementary material for: Neonatal inflammation disrupts a temporally restricted postnatal Numb-enriched microglial state in mice
Source: Nat Commun. 2026 Aug 4;17:9385. doi: 10.1038/s41467-026-76341-6 (PMC13534487; doi:10.1038/s41467-026-76341-6)
Supplement: Supplementary file 4 [file 41467_2026_76341_MOESM2_ESM.pdf]

Description of Additional Supplementary Files

Supplementary Data 1. Marker genes for all microglial states in the normal saline dataset.

Supplementary Data 2. Gene sets used for UCell scoring.

Supplementary Data 3. Marker genes for cell populations at postnatal day 12.

Supplementary Data 4. Marker genes for all cell clusters.

Supplementary Data 5. Marker genes for all microglial states in the LPS dataset.

Supplementary Data 6. Marker_Genes_for_ALL_LPS_Mg_group compare

Supplementary Data 7. Marker genes for cell populations at postnatal day 3.

Supplementary Data 8. Marker genes for microglial subclusters at postnatal day 3.

Supplementary Data 9. Marker genes for border-associated macrophages (BAMs).
